# A near telomere-to-telomere genome assembly of the Jinhua pig: enabling more accurate genetic research

**DOI:** 10.1093/gigascience/giaf048

**Published:** 2025-05-15

**Authors:** Caiyun Cao, Jian Miao, Qinqin Xie, Jiabao Sun, Hong Cheng, Zhenyang Zhang, Fen Wu, Shuang Liu, Xiaowei Ye, Huanfa Gong, Zhe Zhang, Qishan Wang, Yuchun Pan, Zhen Wang

**Affiliations:** College of Animal Sciences, Zhejiang University, Hangzhou, Zhejiang 310058, China; Hainan Institute of Zhejiang University, Building 11, Yongyou Industrial Park, Yazhou Bay Science and Technology City, Yazhou District, Sanya 572025 Hainan, China; College of Animal Sciences, Zhejiang University, Hangzhou, Zhejiang 310058, China; College of Animal Sciences, Zhejiang University, Hangzhou, Zhejiang 310058, China; College of Animal Sciences, Zhejiang University, Hangzhou, Zhejiang 310058, China; College of Animal Sciences, Zhejiang University, Hangzhou, Zhejiang 310058, China; College of Animal Sciences, Zhejiang University, Hangzhou, Zhejiang 310058, China; College of Animal Sciences, Zhejiang University, Hangzhou, Zhejiang 310058, China; College of Animal Sciences, Zhejiang University, Hangzhou, Zhejiang 310058, China; College of Animal Sciences, Zhejiang University, Hangzhou, Zhejiang 310058, China; College of Animal Sciences, Zhejiang University, Hangzhou, Zhejiang 310058, China; College of Animal Sciences, Zhejiang University, Hangzhou, Zhejiang 310058, China; College of Animal Sciences, Zhejiang University, Hangzhou, Zhejiang 310058, China; Hainan Institute of Zhejiang University, Building 11, Yongyou Industrial Park, Yazhou Bay Science and Technology City, Yazhou District, Sanya 572025 Hainan, China; College of Animal Sciences, Zhejiang University, Hangzhou, Zhejiang 310058, China; Hainan Institute of Zhejiang University, Building 11, Yongyou Industrial Park, Yazhou Bay Science and Technology City, Yazhou District, Sanya 572025 Hainan, China; College of Animal Sciences, Zhejiang University, Hangzhou, Zhejiang 310058, China

**Keywords:** pig genome assembly, HiFi and ONT sequencing, gapless reference genome

## Abstract

**Background:**

Pigs are crucial sources of meat and protein, valuable animal models, and potential donors for xenotransplantation. However, the existing reference genome for pigs is incomplete, with thousands of segments and centromeres and telomeres missing, which limits our understanding of the important traits in these genomic regions.

**Findings:**

We present a near-complete genome assembly for the Jinhua pig (JH-T2T) and provide a set of diploid Jinhua reference genomes, constructed using PacBio HiFi, ONT long reads, and Hi-C reads. This assembly includes all 18 autosomes and the X and Y sex chromosomes, with only 6 gaps. It features annotations of 46.90% repetitive sequences, 33 telomeres, 17 centromeres, and 23,924 high-confident genes. Compared to the Sscrofa11.1, JH-T2T closes nearly all gaps, extends sequences by 177 Mb, predicts more intact telomeres and centromeres, and gains 799 more genes and loses 114 genes. Moreover, it enhances the mapping rate for both Western and Chinese local pigs, outperforming Sscrofa11.1 as a reference genome. Additionally, this comprehensive genome assembly will facilitate large-scale variant detection.

**Conclusions:**

This study produced a near-gapless assembly of the pig genome and provides a set of haploid Jinhua reference genomes. Our findings represent a significant advance in pig genomics, providing a robust resource that enhances genetic research, breeding programs, and biomedical applications.

## Data description

### Context

The pig (*Sus scrofa*) is not only economically important due to its role as a food source but also serves as a medical model and potential xenotransplantation donor because of its anatomical and physiological similarities to humans [1, 2]. Understanding the genome and gene content of candidate species, including pigs, is crucial for selecting the best animal model species for pharmacological or toxicological studies. High-quality, fully annotated genome sequences are essential for gene editing, producing improved animal models for research, or providing cells and tissues for xenotransplantation, as well as enhancing productivity [3, 4].

Despite the availability of several high-quality pig reference genomes, including those of the European Duroc [5], Ninxiang [6], Meishan [7], and Jinhua [8, 9] pig genomes, these assemblies remain incomplete in genomic regions of repetitive sequences, centromeres, and telomeres [5–9]. A gap-free genome is the ultimate goal of genome assembly, crucial for improving the accuracy of read mapping and variant calling for individuals sequenced with short and long reads [10], and offers new opportunities for identifying unique genes and structural variations (SVs) [11, 12]. However, to date, a gapless pig reference genome has not yet been reported.

Advancements in new sequencing technologies and computational algorithms have ushered in the era of telomere-to-telomere (T2T) assemblies [13]. Specifically, third-generation sequencing technologies, which generate long reads enabling whole-genome assembly, have improved both experimental methods and algorithms. For example, Pacific Biosciences (PacBio) methods can generate ∼10 kb long HiFi reads with 99% accuracy, while Oxford Nanopore Technologies (ONT) recently developed an ultra-long read method producing reads with an average length of ∼50 kb, extending up to ∼100 kb, with the longest reads reaching hundreds of kb [14–16]. HiFi reads can assist in assembling complex genomic regions [17], while the ONT ultra-long reads can help assemble genomic regions with tandem duplications [18]. The application of third-generation sequencing and assembly technologies to high-fidelity long reads will contribute to the creation of gap-free genome assemblies across hundreds of species [19].

Therefore, we assembled a nearly gap-free T2T genome of the Jinhua pig—one of China’s four renowned indigenous breeds, famous for its superior meat quality and high-quality Jinhua ham [20]—using PacBio HiFi and ONT long reads. This T2T genome assembly marks a significant advance in pig genomics. It offers enhanced resources for research in pig genetics, genomics, and biomedical applications. This assembly overcomes the limitations of previous incomplete assemblies, serving as a robust platform for various downstream comparative genomic analyses and providing new insights into the complex traits of pigs.

## Methods

### Sample collection

Fresh blood was collected from a healthy male Jinhua pig at the National Jinhua Pig Conservation Farm in Zhejiang, China, in 2022 (Supplementary Fig. S2H). Ear tissue samples were collected from its parents.

### DNA extraction, library construction, and sequencing

#### DNA extraction

High-molecular-weight DNA was extracted using the cetyltrimethylammonium bromide (CTAB) method and purified with a QIAGEN Genomic Kit (catalog no. 13343, QIAGEN, Hilden, Germany). Ultra-long DNA was extracted using the sodium dodecyl sulfate (SDS) method [21], omitting the purification step to maintain DNA length. DNA purity was assessed using a NanoDrop One UV-Vis spectrophotometer (Thermo Fisher Scientific). DNA degradation and contamination were monitored on 1% agarose gels. DNA concentration was measured with a Qubit 4.0 fluorometer (Thermo Fisher Scientific).

#### PacBio library preparation and sequencing

SMRTbell target-size libraries were prepared according to PacBio’s standard protocol (Pacific Biosciences, CA, USA) using 15–18 kb preparation solutions. The main steps included: (1) DNA shearing: high-quality DNA samples (primary band >30 kb) were selected and randomly fragmented into 15–18 kb pieces using a g-TUBE (Covaris, MA, USA); (2) DNA damage repair, end repair, and A-tailing; (3) blunt-end ligation: hairpin adapters from an SMRTbell Express Template Prep Kit 2.0 (Pacific Biosciences) were ligated; (4) template purification: imperfect SMRTbell templates were removed with EXOⅢ (from 3′-hydroxyl termini and nicks) and EXOVII (from 5′-termini) treatment; (5) size selection: performed using the bluePippin system. Next, AMPure PB beads were used to concentrate and purify the templates. Then, the sequencing was performed on a PacBio Sequel II (RRID:SCR_017990) instrument with Sequencing Primer V2 and a Sequel II Binding Kit 2.0 at Novogene Co., Ltd (Beijing, China).

#### ONT library preparation and sequencing

Libraries were prepared using an SQK-LSK110 ligation kit following the standard protocol. The purified library was loaded onto primed R9.4 Spot-On Flow Cells and sequenced using a PromethION sequencer (Oxford Nanopore Technologies, Oxford, UK) with 48 h runs at Wuhan Benagen Technology Co., Ltd (Wuhan, China). Base calling of raw data was performed using Oxford Nanopore GUPPY software (v. 0.3.0) (RRID:SCR_003756).

#### Hi-C library preparation and sequencing

For Hi-C sequencing, purified DNA was digested with 100 U DpnII and incubated with Biotin-14-dATP. The ligated DNA was sheared into fragments of 300–600 bp, blunt-end repaired, and A-tailed, followed by purification through biotin–streptavidin-mediated pulldown. The Hi-C libraries were quantified and sequenced using an Illumina NovaSeq (RRID:SCR_016387).

#### Whole-genome resequencing

For whole-genome resequencing, total genomic DNA was isolated from fresh blood using the CTAB method. A 150 bp paired-end library with insert sizes of 350 bp was constructed for each individual following standard Illumina library preparation protocols (Illumina). Meanwhile, PCR-free libraries were prepared with an Illumina TruSeq DNA PCR-free library prep kit (Illumina) according to the manufacturer’s instructions. The qualified libraries were then sequenced using an Illumina Hi Seq X Ten platform (RRID:SCR_016387) to produce 150 bp paired-end reads.

### RNA extraction, library construction, and sequencing

For RNA-seq, 19 samples were collected from 19 different tissues (hypothalamus, midbrain, hypophysis, cerebellar cortex, cerebellar medulla, amygdala, pineal, occipital, hippocampus, striatum, parietal, frontal, temporal, muscle, jejunum, ileum, caecum, colon, and duodenum) in one Jinhua pig. Total RNA was isolated using an RNAprep Pure Plant Kit (TIANGEN, Beijing, China). The total RNA of all tissues was prepared for mRNA sequencing by using TRizol reagent. RNA integrity and yield were assessed with an RNA Nano 6000 Assay Kit used in a Bioanalyzer 2100 system (Agilent Technologies, Santa Clara, CA, USA) and a NanoPhotometer spectrophotometer (IMPLEN, Westlake Village, CA, USA). For each sample, 3 μg of RNA was used to create sequencing libraries using an NEBNext Ultra RNA Library Prep Kit for Illumina (NEB, Ipswich, MA, USA) following the manufacturer’s instructions. Index numbers were added to identify each sample’s sequences. Finally, the clustered libraries were sequenced on an Illumina HiSeq platform (RRID:SCR_016387), generating 150 bp paired-end reads.

### Genome size estimation

To estimate the pig genome size and address potential issues such as sister chromatid merging and repetitive sequences, we used *k*-mer analysis with jellyfish software, v. 2.2.10 (RRID:SCR_005491) [22]. The command “Jellyfish count -G 2 -m 17 -C” and “histo kmercount” were used to calculate the *k*-mer count and generate histograms, respectively.

### Genome assembly

The main goal of this study was to create a high-quality, gapless assembly of the Jinhua pig, comprising 18 autosomes and 2 sex chromosomes (X and Y) and assemble the autosomes of the haplotype-resolved genomes (JH.mat and JH.pat). The assembly process followed the Vertebrate Genomes Project (VGP) assembly pipeline [19] with modifications (Supplementary Fig. S1). First, the initial assembly was constructed using PacBio HiFi reads and ONT ultra-long reads. For the PacBio assemblies, consensus reads (HiFi reads) were generated using CCS software (RRID:SCR_024379) with the default parameters. HiFi reads were then assembled using Hifiasm v. 0.16.1-r375 (RRID:SCR_021069) with default parameters [15, 23]. ONT reads were assembled using NextDenovo v/ 2.5.0 (RRID:SCR_025033) [24] with default parameters genome_size = 2660.49 M. Second, an auxiliary assembly was performed using Allhic (RRID:SCR_022750) [25] and juicebox (RRID:SCR_021172) [26] to improve the Hifiasm output assembly with the help of Hi-C reads. Allhic was utilized to assign the assembled contigs/scaffolds to near-chromosome level. The chromosome interaction intensity, based on juicebox (RRID:SCR_021172), was used for manual correction. NextPolish2 (RRID:SCR_025232) [27] was used to polish the assembly with the default parameter. The initial assembly for the autosomes of the haplotype-resolved genomes was performed using hifiasm v. 0.16.1 (RRID:SCR_021069) [15] and verkko v. 1.1 [28] based on the trio mode with HiFi reads, ultra-long ONT reads, and the parents’ short reads.

### Gap filling

To fill the gaps in the genome assembly, we used winnowmap v. 1.11 (RRID:SCR_025349) software with parameters *k* = 15, −MD [24]. This process involved comparing the hole-filling data (error-corrected ONT genome versions, and HiFi or ONT reads) with the genomic gap intervals. The priority for gap-filling steps was given first to error-corrected genome versions, followed by ONT and HiFi reads. Using this approach, we reduced the number of gaps from 63 to 14. The remaining 14 gap regions could not be adequately covered by the assembly/ONT/HiFi data due to a lack of good reads. We then mapped these gap regions with Hi-C data, generated Hi-C interactions, and imported them into juicebox (RRID:SCR_021172) [26]. After re-examining the Hi-C interaction map, we removed 3 upstream contigs on chr3 and several downstream contigs on chr13, resulting in 2 gaps and 0 gaps on chr3 and chr13, respectively. The final JH-T2T genome has only 6 gaps.

### Datasets and their sources

Genotypes from 938 individuals were collected from the PHARP database [29] (Supplementary Table S7). Additionally, 92 RNA-seq data from 10 pig populations, covering 11 different tissues (brain, heart, liver, spleen, lungs, kidneys, fat, muscle, ovaries, testicles, and intestinal segments) were downloaded from the NCBI database (Supplementary Table S8).

### Genome assembly quality assessment

To systematically evaluate the quality of the genome assembly, we conducted the following assessment. (1) Gene completion—gene completion of the assembly was evaluated using BUSCO (RRID:SCR_015008) (v. 5.4.3) [30] with the mammalia_odb10 dataset [24]. (2) Genome continuity—genome continuity was assessed by calculating contig N50 length using QUAST (RRID:SCR_001228) v. 5.0.2 [31]. (3) Quality value (QV)—Merqury (RRID:SCR_022964) [32] was used to calculate QV, combining Illumina reads. (4) Reads mapping rate and coverage—we mapped the WGS (*n* = 153), and HiFi (*n* = 1) and ONT (*n* = 1) reads to the assembly using BWA-MEM2 (RRID:SCR_010910) and minimap2 (RRID:SCR_018550) [33], respectively. We then calculated their mapping rates and coverages.

### Identification of telomeres and centromeres

In vertebrates, telomeres consist of conserved repetitive sequences as described in the Telomere Database [34]. Here, we also used the vertebrate telomeric repeat (6-mer TTAGGG/CCCTAA) to identify telomeres using the Tidk v. 0.2.0 tool [35] and the Seqtk v. 1.4 [36] telo module. Tidk detected telomeric repeat sequences throughout all the sequences; the final telomere identification results are based on the Seqtk telo findings. Centromics (RRID:SCR_025253) [37] software was used to pinpoint centromere regions. This tool utilizes characteristics such as a high density of short tandem repeats and a low density of genes, which are typical of centromere regions, to identify centromeres in the JH-T2T genome.

### Repeat annotation

The homologous repeat annotation library for the JH-T2T genome was constructed by extracting mammalian repeat sequences from a combined library comprising Repbase (release 20181026) (RRID:SCR_021169) and Dfam v. 3.2 [38, 39]. RepeatModeler v. 2.0.3 (RRID:SCR_015027) [40] was then used to analyze and predict repeat sequences based on this library. Finally, Repeatmasker v. 4.1.2 (RRID:SCR_012954) [41] was utilized to annotate the transposable elements (TEs) in the JH-T2T genome using the custom non-redundant set of repeats.

### Gene annotation

To annotate the protein-coding genes in the JH-T2T genome, we used a combination of ab initio, homology-based, and transcriptome-based prediction methods. For the ab initio gene prediction, MAKER (RRID:SCR_005309) [42] was applied to predict gene structures in the masked JH-T2T genome. High-quality protein sequences from Ensembl release 106 were used for gene annotation, including pigs and 11 other mammals (*Homo sapiens, Equus caballus, Canis lupus, Bos taurus, Capra hircus, Ovis aries, Camelus dromedaries, Delphinapterus leucas, Balaenoptera musculus, Physeter catodon*, and *Tursiops truncates*). Additionally, transcripts from 111 samples (Supplementary Table S8) generated from our RNA-seq data and publically available data were processed using HISAT2 v. 2.2.1 (RRID:SCR_015530) and StringTie v. 2.1.4 (RRID:SCR_016323) [43, 44]. The initial round of gene annotation utilized protein sequences and transcripts. BLASTN (RRID:SCR_011822) [45] with an e-value cutoff of 1 × 10^−10^ was used to map these homologous protein sequences to the JH-T2T genome. Only protein sequences with the highest-scoring alignments, having a minimum identity score greater than 80%, were retained to predict putative gene models using Exonerate v,2.4.0 (RRID:SCR_008417) [46]. The second-round transcript-based gene prediction involved training SNAP v. 2006-07-28 [47] and AUGUSTUS v3. .4.0 (RRID:SCR_008417) [48] with predicted gene models to predict genes.

### Functional annotation of protein-coding genes

We used three methods to annotate functions of protein-coding genes. First, protein sequence similarities were searched against the NCBI non-redundant protein database and the Swiss-Prot database (RRID:SCR_021164) [49, 50] using BlastP software (RRID:SCR_005891) [45]. Second, protein domain and Gene Ontology term annotations were performed using InterProScan (RRID:SCR_005829) [51]. Third, KEGG annotation was performed with kofam_scan (RRID:SCR_012773) [52]. These methods provide complementary approaches, combining sequence similarity, domain analysis, and pathway information to gain insights into the potential functions of these genes in the JH-T2T genome. Additionally, the expression of these genes was also examined using the RNA-seq data. We first used fastp (RRID:SCR_016962) [53] to remove the low-quality reads and adapters in the raw RNA-seq reads, and mapped the remaining reads to the transcripts of high-quality predicted genes by Hisat2 (RRID:SCR_015530) [43]. We then used StringTie v. 2.1.7 (RRID:SCR_016323) [44] to assemble and quantify transcripts guided by the JH-T2T genome. The transcripts were evaluated based on transcripts per million (TPM) values. A TPM > 0 indicated the presence of a transcript in a sample. If a transcript occurred in at least one sample, it was considered as validated, indicating the expression of the predicted gene.

### Global comparison of the Sscrofa11.1 and JH-T2T genome

To assess variation in chromosome-scale synteny, we compared the JH-T2T and Sscrofa11.1 [5] assemblies. We began by aligning the two genomes using NUCmer (RRID:SCR_018171) [54] with parameters -l 100 -c 1000, refining the results with Delta-filter using parameters -i 95 -l 100 -1. Additionally, Minimap2 (RRID:SCR_018550) [33] with parameters -cx asm5 -t8 -cs was used to align Sscrofa11.1 to JH-T2T. The optimal alignments were used for SNPs and indels calling with paftools.js (RRID:SCR_018550) [33]. To detect structural variants (SVs), we used Minimap2 (RRID:SCR_018550) with parameters -a -x asm5 -cs -r2k to to get the best alignments, followed by SV calling with svim-asm using the haploid parameter [55]. To explore the functional implications of deleterious variants, we selected genes with such variants for enrichment analysis using KOBAS v. 3.0 (RRID:SCR_006350) [56]. Next, we used Liftoff v. 1.6.2 [57] to map genes between Sscrofa11.1 and JH-T2T, assessing their consistency. Because Sscrofa 11.1 is an assembly of a Duroc pig, we aligned WGS clean reads including JH and Duroc pigs to both assemblies using the BWA-MEM tool (RRID:SCR_010910) with default parameters [58] to examine the coverage and depth of detected SVs. These analyses allowed us to assess the variation in chromosome-scale synteny, identify genetic variants, investigate missing genes, and validate SVs in the JH-T2T and Sscrofa11.1.

### Selection signatures between JH and Duroc pigs in large SV regions

To examine selection signatures in large SV regions between Jinhua and Duroc pigs, we analyzed genotypes from 289 Jinhua and 616 Duroc pigs (Supplementary Table S7). We used three approaches to detect selection signals: fixation index (FST), nucleotide diversity ratio (θπ), and cross-population extended haplotype homozygosity (XP-EHH). The FST and θπ were calculated across the genome using 10 kb non-overlapping sliding windows with VCFtools v. 0.1.16 (RRID:SCR_001235) [59]. XP-EHH was determined with selscan v. 1.2.0 [60], averaging XP-EHH scores over 10 kb non-overlapping sliding windows. Genomic regions in the top 5% values for at least one selection signature were identified as selective sweeps. Genes in these selective sweep regions were considered candidate highly related genes.

## Results

### T2T assembly of JH pig genome

**Figure 1:**
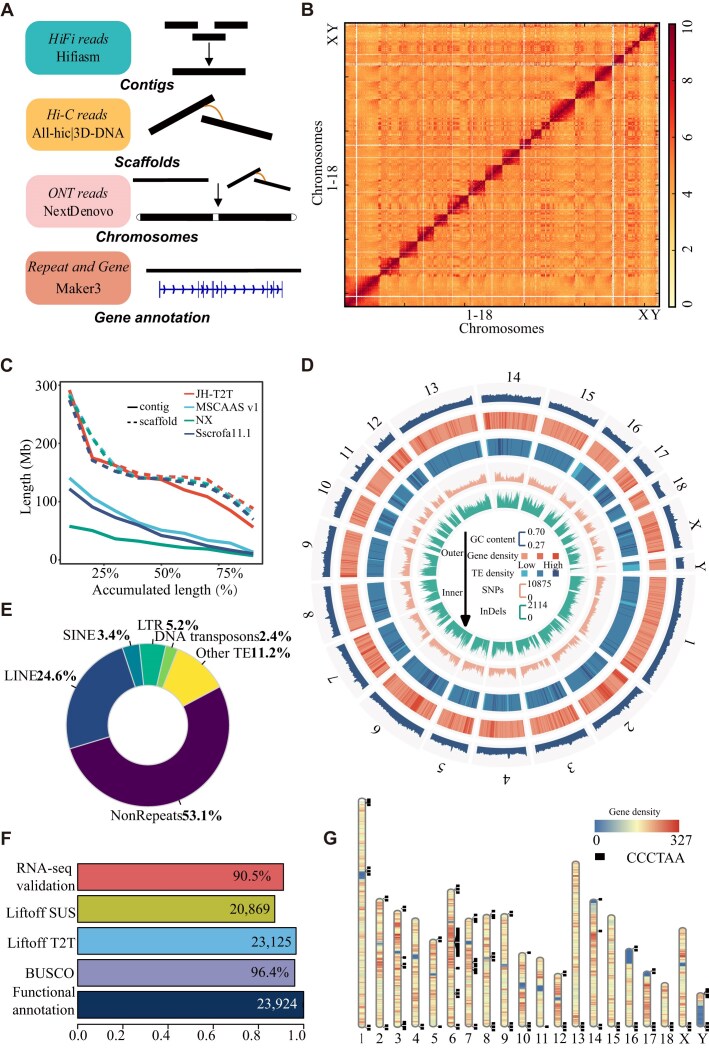
Summary of JH-T2T pig genome assembly. (A) Schematic diagram illustrating the pipeline for genome assembly and annotation. (B) Hi-C chromatin interactions of the assembled JH-T2T genome. (C) Comparison of the contiguity between released assemblies and JH-T2T assembly. (D) Landscape of the assembled JH-T2T genome, showing chromosomes, GC contents, gene, repeat and TE density, SNPs, and InDels in different tracks from outer to inner. (E) Composition ratio of repeat elements in JH-T2T. (F) Gene annotations of JH-T2T. (G) Genome-wide telomere portrait of JH-T2T. The black boxes indicate chromosomal loci of the tandemly repeated telomeric motif in the primary assembly. The heatmap shows the chromosome-wide gene density in non-overlapping 1 Mb windows.

We generated a total of 51.10× sequence coverage of raw PacBio HiFi data (135.95 Gb, read N50 18.32 Kb), 136.65× sequence coverage of ultralong ONT data (363.55 Gb, read N50 52.17 Kb), 94× sequence coverage of Hi-C data, and 50× sequence coverage of WGS data for assembling the JH pig genome (Supplementary Fig. S2A–D and Supplementary Table S1). Using the HiFi reads, we assembled the initial PacBio HiFi assembly, which had a total length of 2.72 Gb and consisted of 187 contigs (contig N50 84.83 Mb; Supplementary Fig. S2E). The initial ONT assembly had a total length of 2.28 Gb and consisted of 93 contigs (contig N50 64.34 Mb; Fig. 1A, Supplementary Fig. S2F, and Supplementary Table S2). A second ONT assembly using only the longest ONT reads (87.23 G, read N50 100 kb) had a total length of 2.31 Gb and consisted of 112 contigs (contig N50 72.01 Mb; Supplementary Fig. S2G), which were used to fill the gaps. Because the PacBio HiFi assembly showed a higher quality and contiguity compared with the ONT assembly, we selected it for the backbone of the genome assembly. We used Hi-C data to order and orient these PacBio HiFi contigs, resulting in 20 chromosomes (with 6 gaps, scaffold N50 142.74 Mb) representing chromosomes 1–18, X, Y, and 66 unplaced contigs containing an additional 62.32 Mb (Fig. 1A,B, Supplementary Fig. S2E, and Supplementary Table S3). The PacBio HiFi assembly was further iteratively polished by PacBio HiFi reads, ONT reads, Hi-C data (for scaffolding), and the second ONT assembly, resulting in a near-T2T assembly with a total length of 2.68 Gb (2.61 Gb mounted on the chromosome, mounting rate of 97.67%, scaffold N50 142.75 Mb) and only 6 gaps remaining in chromosomes 2, 3, 8, and 10 (Supplementary Fig. S2E and Supplementary Table S4).

The initial hifiasm haplotype assemblies had total lengths of 2.68 Gb (275 contigs and contig N50 106.25 Mb) and 2.33 Gb (137 contigs and contig N50 80.18 Mb), respectively. The initial verkko haplotype assemblies had total lengths of 2.36 Gb (276 contigs and contig N50 30.78 Mb) and 2.17 Gb (252 contigs and contig N50 25.26 Mb), respectively (Supplementary Table S2). The more continuous contigs of the two assemblies were selected to construct the final haploid assemblies. This results in a maternal assembly with 157 gaps and paternal assembly with 99 gaps. Subsequently, gap closing was performed using TGS-Gapcloser [61] with the verkko haplotype assemblies, resulting 116 and 42 gaps, respectively. The final diploid Jinhua reference genome has NG50 of 147.60 Mb and 143.06 Mb for maternal and paternal genomes, respectively.

### Quality assessment of the final JH-T2T assembly

We conducted a comprehensive assessment of the JH-T2T assembly’s quality and completeness in multiple ways. First, the estimated genome size was determined to be 2.69 Gb, with a heterozygosity rate of 0.38%, consistent with the Sscrofa11.1 genome size (Supplementary Fig. S4A and Supplementary Table S4). Second, 16 of the 20 chromosomes were each represented by a single contig (Supplementary Table S4), indicating superior sequence integrity compared with the current pig reference genome, Sscrofa11.1 (1,117 contigs), and other published pig genomes (Fig. 1C, Table 1, and Supplementary Table S5). Third, the JH-T2T assembly showed high overall base accuracy. The estimated QV score is 55, which corresponds to 99.997% accuracy. The QV scores ranged from 48 to 62 for each chromosome, with 5 chromosomes (chr4, chr9, chr11, chr15, and chr16) having high QV scores greater than 60 (Supplementary Fig. S4C,D and Supplementary Table S4). Fourth, compared to the other 3 genomes, BUSCO analysis revealed that the JH-T2T assembly exhibited the highest percentage of completeness, with approximately 96.4% of the core conserved mammalian genes being fully represented (Fig. 1F, Supplementary Fig. S4B, and Supplementary Table S6). This indicates a near-complete genome assembly. Fifth, the chromosomal interaction maps generated using Hi-C data provided further evidence of the accuracy and reliability of the JH-T2T assembly. Hi-C data revealed that all chromosomes displayed clear intrachromosomal diagonal signals, with no significant interchromosomal signals, confirming the correct order and orientation of all pseudomolecules (Fig. 1B). Sixth, the remapping rates for HiFi reads, ONT reads, and Illumina short reads on JH-T2T assembly were impressively high at 99.90%, 99.99%, and 99.99%, respectively.

**Table 1:** Summary information of JH-T2T, Sscrofa11.1, Ningxiang and MSCAAS v. 1 assemblies.

Terms	JH-T2T	Sscrofa11.1 [5]	Ningxiang [6]	MSCAAS v. 1 [7]
Contig N50 (Mb)	100.5	48.2	26.1	48.1
Contig number	26	1,117	305	152
Scaffold N50 (Mb)	142.7	88.2	139.0	139.0
Scaffold number	20	20	19	19
Gaps	6	103	286	133
Assembly size (Gb)	2.61	2.50	2.44	2.50
Average length of CDS (bp)	1,593	1,668	1,601	1,379
Protein-coding genes	23,924	20,661	20,914	22,855

**Figure 2:**
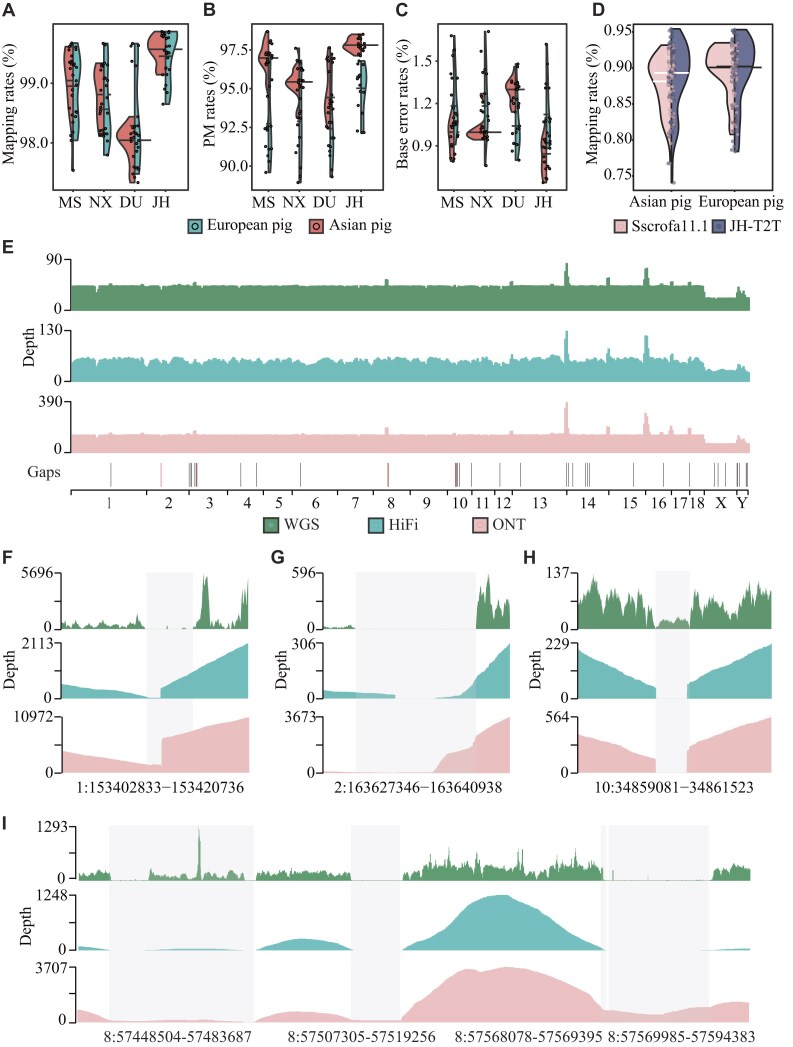
Sequencing coverage, mapping statistics, and gap filling in the JH-T2T assembly. (A–C) Comparison of DNA sequencing read mapping rates, PM rates, and base error rates when whole-genome resequencing reads from Asian (left) and European (right) pigs were mapped to MSCAAS v. 1, Ningxiang (NX), Sscrofa11.1, and JH-T2T genome assemblies, respectively. (D) Comparison of RNA sequencing read mapping rates for Asian (left) and European (right) pigs mapped to the Duroc (Sscrofa11.1) and the JH-T2T genome assembly, respectively. (E) Whole-genome sequence coverage of mapped WGS, HiFi, and ONT reads, showing the gap distribution across chromosomes in JH-T2T. Black indicates filled gaps; red indicates unfilled gaps. (F–I) Whole-genome sequence coverage of mapped WGS (green), HiFi (blue), and ONT (pink) reads specifically in gap regions (1:153402833−153420736, 2:163627346−163640938, 10:34859081−34861523, 8:57448504–57483687 8:57507305–57519256 8:57568078–57569395 8:57569985–57594383).

For alignment-based comparison with other reported genomes, we first utilized WGS data from 30 individuals (depth ranging from 10.00× to 27.14×, Supplementary Table S7), which were mapped to the Sscrofa11.1 (GCA_000003025.6) [5], MS (ASM1795798v1) [7], NX (ASM2056790v1) [6], and JH-T2T genomes. The JH-T2T showed significantly higher mapping rates (98.65–99.87%, Fig. 2A), properly paired mapped rates (92.16–98.51%, Fig. 2B), and lower base error rates (0.64–1.62%, Fig. 2C) compared with other genomes. The average mapping rate for Asian pigs was 99.53% on JH-T2T versus 97.98% on Sscrofa11.1, and for European pigs, 99.48% versus 98.44% (Fig. 2A). The average rate of properly mapped reads for Asian pigs was 97.84% on JH-T2T versus 94.68% on Sscrofa11.1, and for European pigs, 94.78% versus 93.04% (Fig. 2B). Next, mapping 111 RNA-seq data (Supplementary Table S8) from Asian (*n* = 61) and European (*n* = 50) pig breeds showed that JH-T2T was more suitable for analyzing RNA-seq data from Asian pig breeds, with higher mapping rates (88.70%) compared with Sscrofa11.1 (87.67%, Fig. 2D). The average mapping rate of European pigs on JH-T2T and Sscrofa11.1 was similar (89.45% versus 89.58%, Fig. 2D). The above results suggest that JH-T2T will be advantageous for both DNA and RNA sequencing data mapping analysis.

Additionally, only 6 gaps remain in our final JH assembly, with filled gaps ranging from 81 to 35,183 bp, totaling around 268 kb (Supplementary Fig. S5 and Supplementary Table S9). We remapped ONT and HiFi reads to the post-gap-filled genome to confirm the reliability for each filled gap. Most filled gaps were identifiable through ONT or HiFi alignments, and assembly errors in low-coverage regions (LCRs) were corrected via ONT alignments (Fig. 2E and Supplementary Fig. S6). Specifically, the largest two gaps (35 and 24 kb) on chromosome 8 were successfully confirmed by coverage with multiple ONT or HiFi reads (Fig. 2I and Supplementary Figs S5 and S6). The gaps on chromosomes 1, 2, and 10 were also successfully filled, evidenced by coverage with both ONT and HiFi reads (Fig. 2F–H and Supplementary Table S9). Although supported by some read data, the inconsistency of coverage across these gap-filled regions suggests that caution should be used when interpreting findings in these regions, and cross-referencing results with the gap positions (Supplementary Table S9) is advised.

The QVs of JH.mat and JH.pat are 56.73 and 60.07, respectively. The even coverage distribution of ONT and PacBio HiFi reads suggested 3 reliable and continuous assemblies (Supplementary Fig. S3A and B). Further, by comparing the linear genomes of two complete haplotypes, we detected ∼7.23 million single nucleotide variants (SNVs), 1,165,610 small insertions or deletions (indels) (<50 bp), and 26,701 SVs (≥50 bp).

Overall, our assembly quality metrics indicate a near-gapless assembly of the pig genome, and provide a set of diploid JH reference genomes. To the best of our knowledge, this assembly is the first T2T and the most complete pig genome assembly published.

### Genome annotation

The JH-T2T genome assembly provides a gapless T2T sequence for 16 out of 20 chromosomes, marking significant progress over previous incomplete pig genome assemblies [5–7]. About 46.90% of the JH-T2T genome consists of repetitive sequences elements: 24.63% LINEs (long interspersed nuclear elements), 3.40% SINEs (short interspersed nuclear elements), 5.23% LTR (long terminal repeat), 2.44% DNA transposons, 1.13% simple repeats, and 5.21% satellites (Supplemental Table 10 and Fig. 1E).

**Figure 3:**
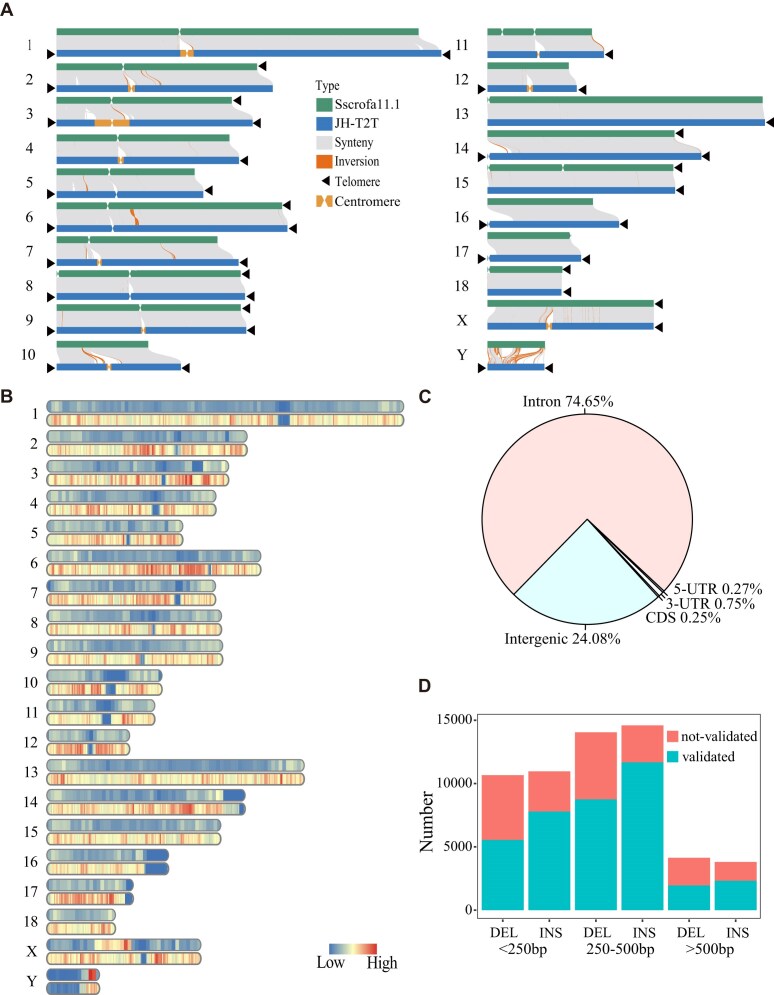
Global comparison of Sscrofa11.1 and JH-T2T genomes. (A) Collinearity between the JH-T2T genome and Sscrofa11.1 genome. Collinear regions are shown by gray lines. Black triangles indicate the presence of telomere sequence repeats. (B) Density distribution of SVs across the JH-T2T genome. (C) Proportions of SVs in 5′UTR, 3′UTR, CDS, introns, and intergenic regions. (D) Percentage of validated SVs categorized by length.

Using the 6-base telomere repeats (TTAGGG/CCCTAA) as a query, 33 out of the anticipated 40 telomeres were identified, with a single telomere detected on chromosomes 2, 4, 11, 13, 15, 18, and X. The average telomere length is 14.71 kb, with approximately 2,451 repeat copies per telomere. The longest telomere spanned 28.20 kb (Fig. 1G and Supplementary Table S4). Putative centromeres were identified in expected locations on chromosomes 1–12, 14, and 16–17 (Fig. 3A, Supplementary Fig. S7 and Supplementary Table S4). For the chromosome assemblies of chr9, 2 regions harbouring centromeric repeats were identified. The tandem repeat identified at the beginning of chr9 is unlikely to represent a second centromere, given the known metacentric karyotype of the pig. Instead, it may represent another form of tandem repeat, which warrants further investigation. We observed that a few chromosomes exhibit a high copy number of telomere repeats. These interstitial or pericentromeric telomeric sequences (ITS) have been evidenced as relics of genome rearrangements in some vertebrate species [62].

Gene annotation of the masked JH-T2T genome was performed using MAKER [42] with evidence from protein homologies and RNA-seq data. A total of 23,924 high-confidence protein-coding genes were predicted (Fig. 1F), which includes 799 newly anchored genes (Supplementary Table S11). To validate these predictions, RNA-seq data from 111 samples showed that 20,110 (90.50%) of the high-confidence genes were expressed in at least 1 sample (Fig. 1F). Gene and repeat distribution across chromosomes follow the typical pattern observed in vertebrate genomes, with higher gene concentrations in GC-rich regions and decreased gene density in repeat-rich distal regions (Supplementary Fig. S2K). Also, the density of genes at telomeres is lower (Fig. 1G).

### Global comparison between the Sscrofa11.1 and JH-T2T genomes

The JH-T2T genome assembly showcases greater completeness and accuracy compared with the Sscrofa11.1 assembly. First, the JH-T2T assembly added approximately 171 Mb (6.8%) to the Sscrofa11.1 assembly. Second, regarding the completeness measured by BUSCOs, the JH-T2T achieved 96.4% of 9,226 BUSCOs, surpassing Sscrofa11.1’s 94.1% (Supplementary Fig. S4B and Supplementary Table S6). Third, the JH-T2T assembly identified 33 telomeres (out of an expected 40, Supplementary Table S4), whereas Sscrofa11.1 captured telomere only at the proximal ends of Sscrofa11.1 chromosome assemblies of SSC2, SSC3, SSC6, SSC8, SSC9, SSC14, SSC15, SSC18, and SSCX. The JH-T2T assembly identified 17 centromeres on chromosomes 1–12, 14, and 16–17 (Supplementary Table S4). Putative centromeres were identified in the expected locations in the Sscrofa11.1 chromosome assemblies for SSC1–7, SSC9, SSC13, and SSC18. Two regions harboring centromeric repeats were identified in the chromosome assemblies of each of SSC8, SSC11, and SSC15. Compared with Sscrofa11.1, JH-T2T predicted a greater number of more intact telomeres and centromeres [5]. These enhancements highlight the superior quality and utility for genomic research of the JH-T2T assembly.

By comparing genes between JH-T2T and Sscrofa11.1, JH-T2T includes 799 newly anchored genes (Supplementary Table S11) involved in 96 KEGG entries, enriching two KEGG pathways and 19 GO terms (Supplementary Fig. S8B and Supplementary Table S12), notably in olfactory (e.g., olfactory transduction) and immunity-related pathways (e.g., cytokine-cytokine receptor interaction, Fc gamma R-mediated phagocytosis, and allergies and autoimmune disease pathways). Moreover, JH-T2T lost 114 genes (Supplementary Table S11), significantly enriching 5 KEGG pathways and 14 GO terms, including steroid hormone biosynthesis and linoleic acid metabolism (Supplementary Table S12).

A comprehensive comparison between JH-T2T and Sscrofa11.1 has identified 58,200 SVs (28,843 deletions and 29,357 insertions, total genome size of 41.5 Mb, rangubg from 50 to 144,010 bp) using Sscrofa11.1 as a reference, with 57,796 medium (50–10,000 bp) and 404 large SVs (≥10 kb) (Supplementary Fig. S8C and Supplementary Table S13). More structural variants were identified in Jinhua pigs than in Ningxiang and Meishan pigs (Supplementary Fig. S8D). SV distributions across chromosomes follow the pattern of higher SV concentrations in repeat-rich regions (Fig. 3B). The majority of the SVs (71.23%) are located in repeat regions, suggesting that repeat sequences are an important source of genetic diversity in pigs. These repeats effectively filled nearly all genome gaps, including the telomeres (Fig. 3A and Supplementary Fig. S7). The majority of these SVs are located in intergenic regions (24.08%) and introns (74.65%), with a minority located within coding sequences (CDS) regions (0.24%) (Fig. 3C). Moreover, 12,129 genes were overlapping with these SVs (Supplementary Table S13). Using the pig QTL database, we found SVs enriched in 65 QTLs associated with six economic traits, such as basophil number, drip loss, and head weight (*P* < 0.01, Supplementary Fig. S8A and Supplementary Table S14), suggesting that SVs potentially impact important economic traits.

Additionally, we simply validated the detected SVs by examining their sequence coverage using the WGS data from 5 JH pigs and 5 Duroc pigs. Using JH-T2T and Sscrofa11.1 as refence and applying a validation criterion that required SV mapping sequence coverage to be 1.00 in one sample and <0.90 in another sample, we confirmed a total of 38,021 SVs (approximately 65.32%), comprising 16,240 DELs and 21,781 INSs, which are associated with 13,967 genes (Supplementary Table 15). Among these, SVs with a length >500 bp were the least frequent (Fig. 3D), highlighting the limitations of SV detection through next-generation sequencing data.

### Large-scale genomic differences in the JH-T2T genome

In our study, we identified 386 large SVs (≥10 Kb) in the JH-T2T genome compared with Sscrofa11.1, including 236 DELs and 150 INSs (Supplementary Table S17). These SVs affected the presence or absence of 212 genes between the 2 genomes. Notably, 101 insertions in the Sscrofa11.1 genome contained an additional 100 genes (Supplementary Table S16). The majority of these genes are olfactory receptor genes, which are significantly enriched in olfactory transduction (including previously reported pig olfactory transduction genes such as *OR8S1* and novel genes such as *OR8B3, OR2V2*, and *OR7A17* (Supplementary Table S17)).

The large SVs also harbored genes related to important economic traits. For example, the *CYP2C18* gene, linked to elevated backfat skatole levels in commercial pig populations [63], was located in the largest SV (∼144.0 kb) on chromosome 14 of JH-T2T, which was located in the selective sweep (Supplementary Fig. S9A–C and Supplementary Table S17). Similarly, an insertion (∼22.2 kb) in the *GPAM* gene, a marker for intramuscular fat content (IMF) content in the musculus longissimus dorsi (MLD) [64], was observed (Supplementary Fig. S9A,B and Supplementary Table S18). The large SVs also contained genes related to immune response, such as *LY9, ITLN2*, and *CHIA* (Supplementary Table S17). The *LY9* gene region indicated positive selection in DU pigs (Supplementary Fig. S10B), associated with immune response regulation [65]. The *ITLN2* and *CHIA* genes have been reported to link to asthma susceptibility in humans [66]. Those findings may be linked to the asthma susceptibility of the JH pig. An insertion (∼15.03 kb) in the *SLA-DOB* gene (Supplementary Table S16) serves the immune system’s response and is relevant to transplant rejection [67].

## Discussion

In this study, we built the first T2T pig genome assembly, marking a significant milestone in pig genomics. Our JH-T2T genome assembly demonstrated remarkable improvements over existing assemblies [5–8], both in terms of completeness and quality. Notably, this T2T genome assembly left only six gaps in chromosomes 2, 3, 8, and 10, exceeding the minimum quality standards set by the Vertebrate Genomes Project (VGP) consortium [19].

The high quality of the JH-T2T assembly is evident in its ability to capture complex genomic regions, including repetitive sequences, and telomeres, which were previously inaccessible. This comprehensive coverage addresses the limitations of earlier reference genomes, such as Sscrofa11.1, which contained 544 gaps and lacked repetitive regions, centromeres, and telomeres. By incorporating these regions, the JH-T2T genome provides a more complete and accurate pig reference genome, essential for detailed genetic studies and breeding programs. Similarly, in human, the use of the T2T-CHM13 genome assembly yields a more comprehensive view of SVs genome-wide, with a greatly improved balance of insertions and deletions [10].

Advancements in sequencing technology, especially the ONT ultra-long sequencing method, have greatly facilitated the complete assembly of genomes. The ONT data played a crucial role in filling gaps, particularly in difficult genomic regions such as repetitive regions, centromeres, and telomeres. Many reference genomes have been successively assembled using ONT reads in farm animals, such as cattle [68], chicken [69], and sheep [70]. In our JH-T2T assembly, the number of gaps was reduced from 63 to 14 using ONT contigs.

A key advantage of the T2T genome is its superior performance in improving reference genome mapping. The JH-T2T assembly outperforms Sscrofa11.1 in mapping reads from both Western and Chinese pig populations, minimizing gaps and enhancing read alignment accuracy for both DNA and RNA sequencing data. This improvement is crucial for large-scale variant calling from second- and third-generation sequencing data and functional genomics studies, enabling more precise identification of genetic variants and their associated traits. For example, in human, the T2T-CHM13 assembly was shown to improve the analysis of global genetic diversity based on 3,202 short read-length samples from the 1KGP dataset [10].

Compared with Sscrofa11.1, the JH-T2T genome captures a more comprehensive set of genetic elements. This includes the identification of 799 newly anchored genes not present in Sscrofa11.1, as well as the recognition of 114 genes that were lost in the JH-T2T. This comprehensive capture is made possible by the JH-T2T genome’s ability to fill in gaps and cover repetitive regions, centromeres, and telomeres, which were previously inaccessible. The identification of these novel and lost genes has significant implications for understanding key biological functions, particularly in olfactory function, metabolism, and immune response. Olfactory genes play a critical role in the sensory perception of smell, which is important for behaviors related to feeding, mating, and environmental interaction [71, 72].

Comprehensive comparison between the JH-T2T and Sscrofa11.1 genomes has identified 58,200 SVs. Considering that some of the SVs may be due to incomplete genome assembly of Sscrofa11.1, we validated them with WGS data. SVs with lengths >500 bp were the least frequent (approximately 65.32%) of validated SVs, which may be due to the limited sample size of the WGS data, the validation methodology, or variations in assembly integrity. The JH-T2T genome assembly enables more precise characterization of SVs. This precision is crucial because incomplete assemblies or technological limitations can result in incorrect assemblies or omissions of important SVs.

One of the most critical improvements offered by the T2T assembly is its superior ability to capture SVs that affect important genes. This enhanced coverage enables the accurate identification and characterization of SVs, which are crucial for understanding genetic variation and its influence on phenotypic traits. For example, among the SVs accurately captured by the T2T genome, we identified notable examples such as the largest SV located in the left telomere region of chromosome 14, which includes important genes such as *CYP2C42* and *CYP2C18*. In this study, we systematically characterized large SVs between 2 pig genome assemblies, identifying 204 large SVs with gene–model differences. Most of these large SVs overlapped with candidate regions for selection signatures, underscoring the importance of these SVs for pig population differentiation. Some genes, such as *LGALS12, GPAM*, and *CACNB2*, are implicated in essential metabolic pathways and can influence important economically traits [63]. The large SVs also contained genes related to immune response, such as *LY9, ITLN2*, and *CHIA*, which may be linked to asthma susceptibility in the JH pig [65, 66]. The insertion found in *SLA-DOB*, a gene involved in enhancing the immune system’s response to infection, might be relevant in relation to transplant rejection [73].

## Conclusions

The JH-T2T genome assembly represents a major leap forward in pig genomics. Its high quality and near-complete coverage significantly enhance our ability to capture and characterize SVs, particularly those harboring important genes. This improvement not only refines the reference genome but also serves as a powerful tool for genetic studies and breeding strategies aimed at improving livestock traits.

## Supplementary Material

giaf048_Supplemental_Files

giaf048_Authors_Response_To_Reviewer_Comments_Original_Submission

giaf048_Authors_Response_To_Reviewer_Comments_Revision_1

giaf048_Authors_Response_To_Reviewer_Comments_Revision_2

giaf048_Authors_Response_To_Reviewer_Comments_Revision_3

giaf048_GIGA-D-24-00462_Original_Submission

giaf048_GIGA-D-24-00462_Revision_1

giaf048_GIGA-D-24-00462_Revision_2

giaf048_GIGA-D-24-00462_Revision_3

giaf048_GIGA-D-24-00462_Revision_4

giaf048_Reviewer_1_Report_Original_Submission Martien Groenen, Ph.D. -- 11/20/2024

giaf048_Reviewer_1_Report_Revision_1 Martien Groenen, Ph.D -- 2/5/2025

giaf048_Reviewer_2_Report_Original_Submission Benjamin D Rosen -- 11//27/2024

giaf048_Reviewer_2_Report_Revision_1 Benjamin D Rosen -- 2/27/2025

giaf048_Reviewer_2_Report_Revision_2 Benjamin D Rosen -- 3/12/2025

giaf048_Reviewer_3_Report_Original_Submission Alan L Archibald -- 12/5/2024

## Data Availability

The genomic and transcriptomic sequence data (genome assembly, ONT, PacBio HiFi, Illumina, Hi-C and Transcripts) generated in this study are available under the NCBI BioProject accession PRJNA1238047. The genotype datasets generated and/or analyzed during the current study are available at PHARP [29] and at NCBI under project PRJEB9922, PRJNA343658, PRJNA398176, PRJNA550237, PRJNA531381, PRJNA260763, PRJCA000169, PRJCA016012, PRJEB58030, PRJEB9115, PRJNA186497, PRJNA239399, PRJNA309108, PRJNA322309, PRJNA378496, PRJNA41185, PRJNA487172, PRJNA488327, PRJNA507853, PRJNA530874, PRJNA626370, PRJNA842867, PRJNA843521. The RNA-seq dataset are available at NCBI under BioProject accession number PRJNA311523. All additional supporting data are available in the GigaScience repository, GigaDB [74]. The scripts used to process our datasets have been upload in github [75].

## Additional files

**Supplementary Table S1:** Summary of the sequence data used for JH-T2T assembly.

**Supplementary Table S2:** Statistics of draft assembly.

**Supplementary Table S3:** Statistics on the number and length of clusters of individual chromosomes and genomic mount rate.

**Supplementary Table S4:** Genome statistics, predicted telomeres, and centromeres.

**Supplementary Table S5:** Scaffold and contig length of 4 assemblies.

**Supplementary Table S6:** BUSCOs analysis of JH-T2T, Sscrofa11.1, MS, and NX.

**Supplementary Table S7:** Information for the 939 WGS pigs.

**Supplementary Table S8:** Information for the 111 RNA pigs.

**Supplementary Table S9:** Gap positions of JH-T2T.

**Supplementary Table S10:** Summary of repeat content of JH-T2T.

**Supplementary Table S11:** Genes lost and gained between JH-T2T and Sscrofa11.1.

**Supplementary Table S12:** Genes lost and gained in the enriched KEGG PATHWAY and GO.

**Supplementary Table S13:** List of SVs between JH-T2T and Sscrofa11.1.

**Supplementary Table S14:** Statistics of SV-related QTL.

**Supplementary Table S15:** SV with WGS validation.

**Supplementary Table S16:** Large SVs localized on Sscrofa11.1 reference and overlapping gene ID on large SVs.

**Supplementary Table S17:** Selected large SVs localized on Sscrofa11.1 reference and overlapping gene ID on large SVs.

**Supplementary Table S18:** Large-SV-gene-enriched KEGG PATHWAY and GO.

**Supplementary Figure S1:** Overview of the data-processing pipeline used for assembly and genomic analysis of JH-T2T genome.

**Supplementary Figure S2:** Summary of data used for JH-T2T assembly.

**Supplementary Figure S3:** Haplotype assemblies of paternal and maternal.

**Supplementary Figure S4:** Evaluation of the JH-T2T assembly.

**Supplementary Figure S5:** Replaced gap regions.

**Supplementary Figure S6:** Coverage of WGS, HiFi, and ONT read for the region of 47 filled gaps of the JH-T2T assembly.

**Supplementary Figure S7:** Locations of the predicted centromeres in the JH-T2T assembly.

**Supplementary Figure S8:** SVs between the Sscrofa11.1 and JH-T2T.

**Supplementary Figure S9:** Selective regions on large SVs between the Sscrofa11.1 and JH-T2T genome assembly.

**Supplementary Figure S10:** Selective regions on large SVs between the Sscrofa11.1 and JH-T2T genome assembly.

## Abbreviations

HiFi: High Fidelity; ONT: Oxford Nanopore Technologies; Hi-C: high-throughput chromosome conformation capture; T2T: telomere-to-telomere; CTAB: cetyltrimethylammonium bromide; SDS: sodium dodecyl sulfate; CCS: Circular consensus sequencing; TEs: transposable elements; QV: quality value; BUSCO: Benchmarking Universal Single-Copy Orthologs; NCBI: National Center for Biotechnology Information; VGP: Vertebrate Genomes Project; KEGG: Kyoto Encyclopedia of Genes and Genomes; GO: Gene Ontology; TPM: transcripts per million; SV: structure variation; WGS: whole genome sequencing; LCRs: low-coverage regions; LINEs: long interspersed nuclear elements; SINEs: short interspersed nuclear elements; LTR: long terminal repeat; CDS: coding sequences; QTL: quantitative trait locus; IMF: intramuscular fat content; MLD: musculus longissimus dorsi; DEL: deletion; INS: insertion.

## Declarations

The pigs used in this study were raised in standard commercial breeding facilities that adhered to routine animal husbandry practices and welfare guidelines. All procedures were conducted in accordance with local animal welfare regulations. No additional ethics approval was required as data collection involved routine farm management practices without invasive procedures.
