# Supplementary material for: A near telomere-to-telomere genome assembly of the Jinhua pig: enabling more accurate genetic research
Source: Gigascience. 2025 May 15;14:giaf048. doi: 10.1093/gigascience/giaf048 (PMC12080228; doi:10.1093/gigascience/giaf048)
Supplement: giaf048_GIGA-D-24-00462_Revision_1 [file giaf048_giga-d-24-00462_revision_1.pdf]

GigaScience

The first near-complete genome assembly of pig: enabling more accurate genetic research

--Manuscript Draft--

**Manuscript Number:**	GIGA-D-24-00462R1
**Full Title:**	The first near-complete genome assembly of pig: enabling more accurate genetic research
**Article Type:**	Data Note

Funding Information:

Funding	Recipient
National Key Research and Development Program of China (2021YFD1200802)	Dr Yuchun Pan
National Key Research and Development Program of China (2022YFF1000500)	Dr Zhen Wang
National Key Research and Development Program of China (2023YFD1300404)	Dr Qishan Wang
National Natural Science Foundation of China (32372831)	Dr Yuchun Pan
National Natural Science Foundation of China (32172691)	Dr Zhen Wang
Key Research and Development Program of Zhejiang Province (2021C02068-2)	Dr Zhen Wang
Key Research and Development Program of Zhejiang Province (2021C02068-1)	Dr Yuchun Pan
the Young Scientists Fund of the National Natural Science Foundation of China (32402713)	Dr Huanfa Gong

Abstract:

Background
Pigs are crucial sources of meat and protein, valuable animal models, and potential donors for xenotransplantation. However, the existing reference genome for pigs is incomplete, with thousands of segments and centromeres and telomeres missing, which limits our understanding of the important traits in these genomic regions.
Findings
We present a near complete genome assembly for the Jinhua pig (JH-T2T) and provides a set of diploid JH reference genome, constructed using PacBio HiFi, ONT long reads and Hi-C reads. This assembly includes all 18 autosomes and the X and Y sex chromosomes, with only six gaps. It features annotations of 46.90% repetitive sequences, 33 telomeres, 17 centromeres, and 23,924 high-confident genes. Compared to the Sscrofa11.1, JH-T2T closes nearly all gaps, extends sequences by 177 Mb, predicts more intact telomeres and centromeres, and gains 799 more genes and loses 114 genes. Moreover, it enhances the mapping rate for both Western and Chinese local pigs, outperforming Sscrofa11.1 as a reference genome. Additionally, this comprehensive genome assembly will facilitate large-scale variant detection.
Conclusions
This study produced a gapless and near-gapless assembly of the pig genome, and provides a set of diploid JH reference genome. Our findings represent a significant advancement in pig genomics, providing a robust resource that enhances genetic research, breeding programs, and biomedical applications.
Background
Pigs are crucial sources of meat and protein, valuable animal models, and potential donors for xenotransplantation. However, the existing reference genome for pigs is incomplete, with thousands of segments and centromeres and telomeres missing, which limits our understanding of the important traits in these genomic regions.
Findings
We present a near complete genome assembly for the Jinhua pig (JH-T2T) and provides a set of diploid JH reference genome, constructed using PacBio HiFi, ONT

Powered by Editorial Manager® and ProduXion Manager® from Aries Systems Corporation

	long reads and Hi-C reads. This assembly includes all 18 autosomes and the X and Y sex chromosomes, with only six gaps. It features annotations of 46.90% repetitive sequences, 33 telomeres, 17 centromeres, and 23,924 high-confident genes. Compared to the Sscrofa11.1, JH-T2T closes nearly all gaps, extends sequences by 177 Mb, predicts more intact telomeres and centromeres, and gains 799 more genes and loses 114 genes. Moreover, it enhances the mapping rate for both Western and Chinese local pigs, outperforming Sscrofa11.1 as a reference genome. Additionally, this comprehensive genome assembly will facilitate large-scale variant detection.<br>Conclusions<br>This study produced a gapless and near-gapless assembly of the pig genome, and provides a set of diploid JH reference genome. Our findings represent a significant advancement in pig genomics, providing a robust resource that enhances genetic research, breeding programs, and biomedical applications.
**Corresponding Author:**	ZHEN WANG<br>Zhejiang University College of Animal Sciences<br>Hangzhou, Zhejiang CHINA
**Corresponding Author Secondary Information:**	
**Corresponding Author's Institution:**	Zhejiang University College of Animal Sciences
**Corresponding Author's Secondary Institution:**	
**First Author:**	Caiyun Cao
**First Author Secondary Information:**	
**Order of Authors:**	Caiyun Cao
	Jian Miao
	Qinqin Xie
	Jiabao Sun
	Hong Cheng
	Zhenyang Zhang
	Fen Wu
	Shuang Liu
	Xiaowei Ye
	Huanfa Gong
	Zhe Zhang
	Qishan Wang
	Yuchun Pan
	Zhen Wang
**Order of Authors Secondary Information:**	
**Response to Reviewers:**	RESPONSE LETTER TO THE REVIEWERS<br>Response to Editor's Comments<br><br>Thank you for your email and for providing the reviewers’ comments on our manuscript, "The first near-complete genome assembly of pig: enabling more accurate genetic research" (GIGA-D-24-00462). We appreciate the time and effort the reviewers have taken to evaluate our work and provide constructive feedback. We are pleased to learn that the manuscript is of interest to GigaScience and are committed to addressing the reviewers’ concerns to improve the quality and impact of our study.<br>We have carefully considered all the comments and will address them in a revised version of the manuscript. Below, we provide a brief outline of our revisions:

Powered by Editorial Manager® and ProduXion Manager® from Aries Systems Corporation

Haplotype-resolved assemblies (Reviewers 1):
We agree with the reviewer that haplotype-resolved assemblies would add significant value to the manuscript. In our revision, we included more results of employing a trio-binning approach to generate haplotype resolved assemblies from the two sequenced parental genomes.

Methodological accuracy and result interpretation (All Reviewers):
In response to the other points raised by the reviewers, we are committed to providing a more accurate description of our methods and a careful interpretation of our results in our revision. We believe that addressing these concerns will significantly enhance the quality of our manuscript.

Adaptation to the "Data Note" format:
We acknowledge the editor’s suggestion to consider the manuscript for the Data Note section. We have revised the manuscript, ensuring that the structure and content align with the journal’s formatting guidelines for this article type.

We have submitted a point-by-point response to the reviewers’ comments along with the revised manuscript, detailing all changes made and providing a rebuttal for any points where we may have a differing perspective. We will also ensure that all data (http://alphaindex.zju.edu.cn/ALPHADB/download.html) and code (https://github.com/CCyeah/JH-T2T) are updated and publicly accessible as required.

Thank you again for the opportunity to improve our work. Please do not hesitate to contact us if you have any further questions or require additional information.

Response to Reviewer 1's Comments

Reviewer #1: The manuscript describes the T2T genome assembly for the Chinese pig breed Jinhua, which presents a vast improvement compared to the current reference genome of the Duroc pig TJTabasco (build11.1). The results and methodology use for the assembly are described clearly and the authors show the improvement of this assembly by a detailed comparison with the current reference 11.1. While clearly of interest to be published, several aspects of the manuscript should be improved. Most of these changes are minor modifications or inaccuracies in the presentation of the results. However, there are two major aspects that need further attention.

Response: Thank you for your valuable comments and insightful suggestions on our manuscript. We greatly appreciate your insights, particularly regarding the T2T genome assembly of the Jinhua pig and the identification of selective sweep regions. We have carefully revised the manuscript in line with your recommendations. Below, you will find point-by-point responses addressing the specific modifications made. Thank you for your detailed review and constructive feedback

1.The T2T assembly presented, represents a combination of the two haplotypes of the pig sequenced. I am surprised why the authors did not also develop two haplotype resolved assemblies of this genome. Haplotype resolved assemblies will be the assemblies of choice for future developments of a reference pan-genome for pigs. The authors describe that they have sequenced the two parents of the sequenced F1 individual, so why did they not use the trio-binning approach to also develop haplotype resolved assemblies. I, think adding these to the manuscript would be a vast improvement for this important resource.
Response: We appreciate your suggestion regarding the development of haplotype resolved assemblies. We agree that these assemblies would be invaluable for establishing a more comprehensive reference pan-genome for pigs. In our revision, we included more results of employing a trio-binning approach to generate haplotype resolved assemblies from the two sequenced parental genomes. The initial assembly for the autosomes of the haplotype-resolved genomes was performed by using hifiasm(v0.16.1)[1] and verkko(v1.1)[2] based on the trio mode with HiFi reads, ultra-long ONT reads and the parents’ short reads. The more continuous contigs were selected from the two assemblies to represent. This results in a maternal assembly with 157 gaps and paternal assembly with 99 gaps (Supplementary Table S2). Subsequently, gap closing was performed using TGS-Gapcloser [3] with the verkko assembly, resulting 116 and 42 gaps, respectively. Since our HiFi reads and ultra-long

Powered by Editorial Manager® and ProduXion Manager® from Aries Systems Corporation

ONT reads are limited, the final diploid JH reference genome has NG50 of 147.60 Mb and 143.06 Mb for maternal and paternal genomes, respectively. And we further evaluated JH.mat and JH.pat with quality values (QV) and coverage distribution of ONT and PacBio HiFi reads. By comparing the linear genomes of two complete haplotypes, we detected ~7.23 million single nucleotide variants (SNVs), 1,165,610 small insertions or deletions (indels) (< 50 bp), and 26,701 SVs (≥ 50 bp).We believe this addition will significantly enhance the value of our manuscript and provide a critical resource for future research in swine genomics.

1. Cheng H, Concepcion GT, Feng X, Zhang H, Li H. Haplotype-resolved de novo assembly using phased assembly graphs with hifiasm. Nat Methods. 2021; doi: 10.1038/s41592-020-01056-5.
2. Rautiainen M, Nurk S, Walenz BP, Logsdon GA, Porubsky D, Rhie A, et al.. Telomere-to-telomere assembly of diploid chromosomes with Verkko. Nat Biotechnol. 2023; doi: 10.1038/s41587-023-01662-6.
3. Xu M, Guo L, Gu S, Wang O, Zhang R, Peters BA, et al.. TGS-GapCloser: A fast and accurate gap closer for large genomes with low coverage of error-prone long reads. GigaScience. 2020; doi: 10.1093/gigascience/giaa094.

2.The results described for the identification of selective sweep regions is not very convincing. This analysis shows differences in the genomes of two breeds: Duroc and Jinhua. However, these breeds have a very different origin of domestication of wild boars that diverged 1 million years ago, followed by the development of a wide range of different breeds selected for different traits. Therefore, the comparison made by the authors cannot distinguish between differences in evolution of Chinese and European Wild Boar, more recent selection after breed formation and even drift. To be able to do so, these analyses would need the inclusion of additional breeds and wild boars from China and Europe. Alternatively, the authors can decide to tone down this part of the manuscript or even delete it altogether, as it does not add to the major message of the manuscript.

Response: Thank you for your insightful comments regarding the analysis of selective sweep regions. We acknowledge that the significant evolutionary divergence between the Duroc and Jinhua breeds, along with their distinct domestication histories, complicates the interpretation of our results. Alternatively, we will revise the manuscript to tone down the discussion on selective sweeps, ensuring it aligns more closely with the main message of our study. Your feedback is invaluable for enhancing the clarity and impact of our work.

Minor comments
Line 34: Change the sentence to: "with thousands of segments and centromeres and telomeres missing"
Response: Thank you for your suggestion. We have revised the sentence as suggested.

Line 37: Insert "and Hi-C" after "long reads"
Response: Thank you for your reminder. The phrase "and Hi-C" has been added as recommended.

Line 46: Delete " such as GPAM, CYP2C18, LY9, ITLN2, and CHIA"
Response: Thank you for your suggestion. We have removed the specific gene names from this sentence.

Line 54: Insert "potential" before "xenotransplantation"
Response: Thank you for your suggestion. The word "potential" has been added before "xenotransplantation".

Line 82: Delete "in response to the gap of a T2T-level pig genome" as this does not add anything and the use of "gap" in this context is confusing.
Response: Thank you for your suggestion. We have deleted the phrase "in response to the gap of a T2T-level pig genome" to improve clarity.

Line 93: Change "The fresh blood" to "Fresh blood"
Response: Thank you for your suggestion. The text has been changed to "Fresh blood"

Powered by Editorial Manager® and ProduXion Manager® from Aries Systems Corporation

as suggested.

Line 100: The authors need to provide a reference for the SDS method
Response: We have included a reference for the SDS method in the revised manuscript, which provides insights into library preparation methods. And the SDS method for Ultra-long DNA was extracted was performed at Wuhan Benagen Technology Co., Ltd for your information. If you need more specific information or additional references, please let me know.
1. Chen H, Rangasamy M, Tan SY, Wang H, Siegfried BD. Evaluation of Five Methods for Total DNA Extraction from Western Corn Rootworm Beetles. Lalueza-Fox C, editor. PLoS ONE. 2010; doi: 10.1371/journal.pone.0011963.

Lines 152-153, line 444, and table S6: This is confusing. The authors mention Genotypes from 939 individuals, but in the table it is shown that they have used WGS data. You need to describe how the WGS data was used to call the genotypes for these individuals. Furthermore, in line 444 you mention 289 JH pigs and 616 DU pigs which together is 905. What about the other 34 individuals shown in table S6?
Response: Thank you for your observations regarding Lines 152-153, line 444, and Table S6. We have revised the relevant tables and content to ensure consistency. There is a total of 925 individuals included. The genotype dataset for 905 individual pigs were extracted from the PHARP database as SNP annotations available on the download site. The remaining 20 individuals and 5 JH pigs and 5 DU pigs were used in the form of raw data rather than genotype data. The methods for utilizing this raw data are described in the "Genome Assembly Quality Assessment" section. Thank you for your question, which will help improve the clarity of our study.

Line 244: Replace "were" by "was" and delete "the" before "fastp"
Response: Thank you for your suggestion. We have changed "were" to "was" and removed "the" before "fastp". Your feedback is greatly appreciated and will help improve the clarity of our work.

Lines 287292: Here you use several times "length of xx Gb and yy contigs". This is not correct as the value for the contigs refers to a number and not a length. Rephase e.g. like "length of xx Gb and consisting of yy contigs"
Response: Thank you for your suggestion. We have rephrased this section (a total length of 2.72 Gb and consisting 187 contigs, a total length of 2.28 Gb and consisting 93 contigs) to accurately reflect the meaning of contig lengths and numbers. Your feedback is greatly appreciated and will help improve the clarity of our work.

Line 294: The use of "bone" sems strange. Either use "backbone" or "core"
Response: Thank you for your suggestion. This have been amended to read "selected it for the backbone of the genome assembly," clarifying the intended meaning. Your feedback is greatly appreciated and will help improve the clarity of our work.

Line 306: Replace "chromosome" by "genome"
Response: Thank you for your suggestion. We have replaced "chromosome" with "genome". Your feedback is greatly appreciated and will help improve the clarity of our work.

Lines 308-309: For the comment "Second, 16 of the 20 chromosomes were each represented by a single contig" you refer to figure 1D however from this figure it cannot be seen if the different chromosomes consist of a single or multiple contigs.
Response: Thank you for your comment regarding the representation of the chromosomes in our manuscript. We initially intended to illustrate the individual chromosomes; however, we mistakenly referred to Figure 1D in this context. We have decided to remove this reference to avoid any confusion, as the figure does not clearly indicate whether the chromosomes are represented by single or multiple contigs. We will ensure that the revised manuscript accurately reflects our findings and provides clearer visual representations where needed. Thank you for bringing this to our attention.

Line 346: Do you mean build 11.1 with "historical genome version". If so, please use that instead.
Response: Thank you for your observation regarding Line 346. We recognize that the

term could lead to misunderstandings regarding draft assemblies. To avoid any confusion, we will remove this phrase from the manuscript. Your feedback is greatly appreciated and will help improve the clarity of our work.

Line 349: "post-gap filled"
Response: Thank you for your suggestion. We have corrected this to "post-gap filled". Your feedback is greatly appreciated and will help improve the clarity of our work.

Line 353: The largest gap is 35 kb not 36 kb.
Response: Thank you for your observation regarding Line 353. The largest gap has been accurately reported as 35 kb. Your feedback is greatly appreciated and will help improve the clarity of our work.

Figures 2F-I should be better explained in the legends and the main text (lines 353-358).
Response: We appreciate your feedback regarding the clarity of Figures 2F-I. To enhance the understanding of these figures, we have revised the legends to provide more detailed descriptions of the data presented. Additionally, we have incorporated further explanations in the legends and main text to contextualize the findings and clarify their significance. We believe these changes will improve the overall comprehension of the figures. Thank you for your valuable suggestions.

Lines 378: For the 23,924 genes you refer to supp table S13. However, that table shows a list of SV enriched QTL not these genes. Furthermore, I checked all tables but a table with all the protein coding genes is missing.
Response: Thank you for pointing out the discrepancy regarding Line 378. We mistakenly referenced Supplementary Table S13, which actually contains a list of SV-enriched QTL rather than the 23,924 genes. We will correct this reference in the revised manuscript. Additionally, we plan to publicly share the annotation file that includes detailed information about all the protein-coding genes. Your feedback is greatly appreciated and will help us ensure the accuracy of our manuscript. And we have proceeded to upload the necessary files directly to our public server using the provided credentials so that reviewer can see it when they need (https://alphaindex.zju.edu.cn/ALPHADB/download.html).

Line 380: For the 799 newly anchored genes, refer to table S10. Now you refer to table S17 which shows genes enriched KEGG pathways.
Response: Thank you for your comment regarding Line 380. We apologize for the incorrect reference to Table S17. We meant to refer to Table S11 for the 799 newly anchored genes. We have corrected this reference to table S11 in the revised manuscript to ensure clarity and accuracy. Your feedback is invaluable in helping us refine our work.

Lines 383-386: For the higher gene density in GC rich regions, you refer to figure 1D, but it is impossible to see this correlation from figure 1D. For the density of genes and telomeres, you refer to figure 1G. However, that figure does not show gene densities only repeat densities.
Response: Thank you for your feedback regarding lines 383-386. I appreciate your observations:
We have added a picture (Figure S2) to support it. The green bars show the percentage of each partition and the red line shows the number of genes in the 1000kb partition. We can see that the density of genes in GC-rich regions is higher in 1000k bins, especially when the GC content is below 50%.

Thank you for your observation regarding figure 1G. The figure legend incorrectly refers to repeat densities when it actually displays gene densities, as mentioned in the main text. We will revise the figure legend to accurately represent the content of the figure. Your feedback is appreciated and will help improve the clarity of our manuscript. Thank you for bringing these issues to my attention. Your insights will help improve the clarity and accuracy of the manuscript.

Line 406-407. This should be table S11.
Response: Thank you for your observation regarding Lines 406-407. We have made the necessary correction and now correctly reference Table S11 in the revised

	manuscript. We appreciate your attention to detail, which helps improve the accuracy of our work.<br><br>Lines 409-412: For this result you refer to table S11. However, that table only shows data for the gained genes, not the lost genes.<br>Response: Thank you for highlighting this issue regarding our reference to Table S11. We acknowledge that the table currently only presents data for the gained genes and does not include information on the lost genes. We have referred to the New Table S11 to clarify this and ensure that we provide a comprehensive table that includes both gained and lost genes. Your feedback is greatly appreciated and will help enhance the completeness of our results.<br><br>Lines 419-420: You refer to table S12 and figure 3B, but the information is only shown in figure 3B and not in table S12.<br>Response: Thank you for pointing out the discrepancy regarding our reference to Table S12 and Figure 3B. We will correct the reference to ensure it accurately cites Figure 3B, and we will delete Table S12, as it does not provide relevant information. Your feedback is invaluable in helping us improve the clarity and accuracy of our manuscript.<br><br>Line 420: Replace "were" by "is"<br>Response: Thank you for your suggestion regarding Line 420. We have revised the text to replace "were" with "is" to ensure clarity and accuracy. We appreciate your attention to detail.<br><br>Line 422: Better to use "repeats" instead of "they"<br>Response: Thank you for your suggestion regarding Line 422. We have updated the text to replace "they" with "repeats" for improved clarity. Your feedback is appreciated.<br><br>Line 425: "Moreover, 12,129 genes located in these SVs". Unclear to what "these" refers to and I assume that you mean genes that (partially) overlap with SVs? Also, this is an incomplete sentence (verb missing). Likewise, this number is not very meaningful as many of these SVs are within introns. It is much more informative to mention for how many genes SVs affect the CDS.<br>Response: Thank you for your valuable feedback regarding Line 425. We recognize that the reference to "these" is vague and will clarify that it refers to genes that (partially) overlap with the SVs. We will also revise the sentence to include a verb for completeness.<br><br>Line 433 and table S14: This validation is not clear at all. What exactly are these numbers that are shown? You also mention "greater than 1.00" but the table does not contain any number that is greater than 1.00.<br>Response: Thank you for your comment regarding Line 433 and Table S14. We will clarify the validation process by specifying that we used JH-T2T and Sscrofa11.1 as references, applying a validation criterion that required SV mapping sequence coverage to be 1.00 in one sample and less than 0.90 in another sample. We will ensure that this explanation is clearly presented in the revised manuscript to eliminate any confusion regarding the numbers shown in the table. Your feedback is greatly appreciated.<br><br>Line 435: "Table" not "Tables"<br>Response: Thank you for your observation regarding Line 435. We have corrected "Tables" to "Table" in the revised manuscript. Your attention to detail is appreciated.<br><br>Line 436: Change to " SVs with a length larger than 500 bp"<br>Response: Thank you for your suggestion regarding Line 436. We have made the change to "SVs with a length larger than 500 bp". Your feedback is appreciated.<br><br>The term "invalidate" in figure 3D is rather awkward. Better to use "not-validated" and "validated" in this figure.<br>Response: Thank you for your suggestion regarding the terminology in Figure 3D. We have revised the terms to "not-validated" and "validated" to enhance clarity. Your feedback is appreciated.

	Line 449: This should be Table S16.<br>Response: Thank you for your comment regarding Line 449. We have corrected the reference. Your attention to detail is appreciated.<br><br>Line 452: There is not Table S18<br>Response: Thank you for your observation regarding Line 452. We have corrected the reference.Your feedback is appreciated.<br><br>Lines 484-486: Change to "Similarly, in human, the use of the T2T-CHM13 genome assembly yields a more comprehensive view of SVs genome-wide, with a greatly improved balance of insertions and deletions [61]."<br>Response: Thank you for your suggestion regarding Lines 484-486. We have rephrased the section to read: "Similarly, in human, the use of the T2T-CHM13 genome assembly yields a more comprehensive view of SVs genome-wide, with a greatly improved balance of insertions and deletions [66]." Your feedback has helped enhance the clarity and coherence of our manuscript.<br><br>Lines 500-501: Change to "For example, in human, the T2T-CHM13 assembly was shown to improve the analysis of global"<br>Response: We have updated Lines 500-501 to read: "For example, in humans, the T2T-CHM13 assembly was shown to improve the analysis of global genetic diversity based on 3,202 short read-length samples from the 1KGP dataset." Thank you for your suggestion, which has improved the clarity of this section.<br><br>Lines 517-528: This paragraph should be deleted as these genes have already been annotated and described in previous genome builds including 11.1. Why discuss these genes here? Following that line of thinking, almost every gene of the 20,000 can be discussed.<br>Response: Thank you for your feedback. We appreciate your perspective on the discussion of previously annotated genes. Our intention was to highlight specific genes that may have new insights or relevance in the context of our current findings. However, we understand the importance of maintaining focus and will revise the manuscript to minimize redundancy and ensure that we emphasize novel contributions. Thank you for helping us improve the clarity and relevance of our work.<br><br>Line 532: "%" instead of "%%" and insert "which" after "SVs"<br>Response: Thank you for your valuable feedback. We will make the necessary changes to replace "%%" with "%" and insert "which" after "SVs" in the relevant sections of the manuscript. Your suggestions help enhance the clarity of our work, and we appreciate your attention to detail.<br><br>Lines 537-542: These sentences should be deleted. It is common knowledge that second generation sequencing is not very sensitive to identify SVs. The authors also do not provide any results about dPCR.<br>Response: Thank you for your insightful comments. We acknowledge that the limitations of second-generation sequencing in detecting structural variants (SVs) are widely understood. We will remove the specified sentences to streamline the discussion. We apologize for the oversight regarding the lack of dPCR results. These sentences will be deleted to clarify this point and ensure that it accurately reflects the scope of our study. Your input helps us improve the clarity and integrity of our work. Thank you for your understanding.<br><br>Line 544: "affect" rather than "harbor"<br>Response: We will revise the text to replace "harbor" with "affect" in the relevant section. Your feedback helps enhance the clarity and accuracy of our manuscript, and we appreciate your attention to detail.<br><br>Lines 544-547: This is repetitive and has been stated multiple times so better to delete.<br>Response: Thank you for your comment. We appreciate your observation regarding the repetition in the text. We will review the relevant sections and remove any redundant statements to improve the clarity and conciseness of the manuscript. Your feedback is invaluable in refining our work.<br><br>Line 561: "which is serve to immune system's response and relevant to transplant

Powered by Editorial Manager® and ProduXion Manager® from Aries Systems Corporation

rejection" This is an incorrect sentence and should rephrased.
Response: Thank you for pointing out the error. A possible rephrasing could be: "which serves to enhance the immune system's response and is relevant to transplant rejection." We appreciate your attention to detail and will ensure this correction is made in the manuscript.

Lines 562-568: I don't agree with is statement and suggest to remove it from the discussion.
Response: Thank you for your feedback. We respect your perspective and will remove the statement from the discussion. Your insights are valuable in ensuring the clarity and focus of our manuscript.

Reviewer #2: The first near-complete genome assembly of pig: enabling more accurate genetic research

The authors describe the telomere-to-telomere assembly of a Jinhua breed pig. They sequenced genomic DNA from whole blood with PacBio HiFi and Oxford Nanopore (ONT) long-read technologies as well as Illumina for short reads. They generated HiC data for scaffolding from blood and extracted RNA from 19 tissues for short read RNAseq for gene annotation. A hifiasm assembly was generated with the HiFi data and scaffolded with HiC to chromosome level with 63 gaps. The scaffolded assembly was gap filled with contigs from a NextDenovo assembly of the ONT data bringing the gaps down to 14. Finally, the assembly was manually curated with juicebox somehow closing a further 8 gaps. This needs to be clarified. Standard assembly assessments were performed as well as genome annotation. The authors compared their assembly to the current reference, Sscrofa11.1, and called SVs between the assemblies. The SVs were validated with additional Jinhua and Duroc animals. They then identifiedsignatures of selection present in some of the largest SVs.

General comments:
The manuscript is mostly easy to read but would benefit from further editing for language throughout. The described assembly appears to be high quality and quite contiguous. Although the authors do mention obtaining parental samples and claim the assembly is fully phased, there is no mention of how this was done. There are many additional places where the methods could be described more fully including the addition of parameters used.
Response: We are glad to hear that you found the manuscript mostly easy to read and that the assembly appears to be of high quality. Thank you for your valuable comments and insightful suggestions.
We will ensure to perform a thorough language edit throughout the manuscript to enhance clarity. Regarding the phasing of the assembly, we will provide a clear explanation of the methodology used to achieve the fully phased assembly, including the specific techniques and strategies employed. And we have made sure the bioinformatics pipeline scripts uploaded to our GitHub repository at https://github.com/CCyeah/JH-T2T. Thank you for your detailed assessment of our manuscript. We appreciate your summary of our methods and findings.
We have rephrased the process of manual curation with Juicebox and how it contributed to closing the remaining gaps in our assembly. Additionally, we will ensure that all components of the assembly and validation processes are clearly described to enhance the reader's understanding. And we have made our code available through GitHub (https://github.com/CCyeah/JH-T2T). This repository includes detailed descriptions of the assembly process and validation methods, providing additional context for our research. Your feedback is crucial in improving the clarity and rigor of our work, and we are grateful for your insights.
In our revision, we also included more results of employing a trio-binning approach to generate haplotype resolved assemblies from the two sequenced parental genomes. The initial assembly for the autosomes of the haplotype-resolved genomes was performed by using hifiasm(v0.16.1) [1] and verkko(v1.1) [2] based on the trio mode with HiFi reads, ultra-long ONT reads and and the parents’ short reads. The more continuous contigs were selected from the two assemblies to represent. This results in a maternal assembly with 157 gaps and paternal assembly with 99 gaps (Supplementary Table S2). Subsequently, gap closing was performed using TGS-Gapcloser [3] with the verkko assembly, resulting 116 and 42 gaps, respectively. Since

our HiFi reads and ultra-long ONT reads are limited, the final diploid JH reference genome has NG50 of 147.60 Mb and 143.06 Mb for maternal and paternal genomes, respectively. And we further evaluated JH.mat and JH.pat with quality values (QV) and coverage distribution of ONT and PacBio HiFi reads. By comparing the linear genomes of two complete haplotypes, we detected ~7.23 million single nucleotide variants (SNVs), 1,165,610 small insertions or deletions (indels) (< 50 bp), and 26,701 SVs (≥ 50 bp).We believe this addition will significantly enhance the value of our manuscript and provide a critical resource for future research in swine genomics.

1. Cheng H, Concepcion GT, Feng X, Zhang H, Li H. Haplotype-resolved de novo assembly using phased assembly graphs with hifiasm. Nat Methods. 2021; doi: 10.1038/s41592-020-01056-5.
2. Rautiainen M, Nurk S, Walenz BP, Logsdon GA, Porubsky D, Rhie A, et al.. Telomere-to-telomere assembly of diploid chromosomes with Verkko. Nat Biotechnol. 2023; doi: 10.1038/s41587-023-01662-6.
3. Xu M, Guo L, Gu S, Wang O, Zhang R, Peters BA, et al.. TGS-GapCloser: A fast and accurate gap closer for large genomes with low coverage of error-prone long reads. GigaScience. 2020; doi: 10.1093/gigascience/giaa094.

Specific comments:
Line 39 - Figure 1 only displays 34 telomeres, not 35. Additionally, I was only able to detect 33 telomeres using seqtk telo. Seqtk only reports telomeres at the beginning and end of sequences, digging further, the telomere on chr2 is ~59kb from the end of the chromosome, perhaps indicating a misassembly.
Response: Thank you for your careful review and observations regarding Figure 1. We utilized the vertebrate telomeric repeat (6-mer TTAGGG/CCCTAA) to identify telomeres using the Tidk (v0.2.0) tool. This software detected telomeric repeat sequences throughout all the sequences, as displayed in Figure 1. In response to your suggestion, we have re-identified the telomeres using seqtk telo. We will correct the telomere counts to accurately reflect the presence of 33 telomeres in Table S4 and the relevant sections, particularly regarding the telomere on chromosome 2. Thank you for your valuable guidance on this matter.

Lines 79-81 - there are not hundreds of species with gap free genome assemblies and reference 19 does not claim that there are.
Response: We appreciate your insight and will modify the statement to accurately represent that “the application of third-generation sequencing and assembly technologies to high-fidelity long reads will be contributed to the creation of gap-free genome assemblies across hundreds of species”.

Line 82 - the assembly is not gap-free, replace with "nearly gap-free"
Response: We have revised the text to describe the assembly as "nearly gap-free" to better reflect its status.

Line 95 - were these parental tissue samples ever used?
Response: Thank you for your insightful comment regarding the parental tissue samples. We will clarify whether the parental tissue samples were utilized in the assembly process and detail how they were incorporated. In our revision, we also included more results of employing a trio-binning approach to generate haplotype resolved assemblies from the two sequenced parental genomes. The initial assembly for the autosomes of the haplotype-resolved genomes was performed by using hifiasm (v0.16.1)[1] and verkko(v1.1)[2] based on the trio mode with HiFi reads, ultra-long ONT reads and and the parents’ short reads. The more continuous contigs were selected from the two assemblies to represent. This results in a maternal assembly with 157 gaps and paternal assembly with 99 gaps (Supplementary Table S1). Subsequently, gap closing was performed using TGS-Gapcloser [3] with the verkko assembly, resulting 116 and 42 gaps, respectively. Since our HiFi reads and ultra-long ONT reads are limited, the final diploid JH reference genome has NG50 of 147.60 Mb and 143.06 Mb for maternal and paternal genomes, respectively. And we further evaluated JH.mat and JH.pat with quality values (QV) and coverage distribution of ONT and PacBio HiFi reads. By comparing the linear genomes of two complete haplotypes, we detected ~7.23 million single nucleotide variants (SNVs), 1,165,610 small insertions or deletions (indels) (< 50 bp), and 26,701 SVs (≥ 50 bp).We believe this addition will significantly enhance the value of our manuscript and provide a critical resource for future research

in swine genomics.
1. Cheng H, Concepcion GT, Feng X, Zhang H, Li H. Haplotype-resolved de novo assembly using phased assembly graphs with hifiasm. Nat Methods. 2021; doi: 10.1038/s41592-020-01056-5.
2. Rautiainen M, Nurk S, Walenz BP, Logsdon GA, Porubsky D, Rhie A, et al.. Telomere-to-telomere assembly of diploid chromosomes with Verkko. Nat Biotechnol. 2023; doi: 10.1038/s41587-023-01662-6.
3. Xu M, Guo L, Gu S, Wang O, Zhang R, Peters BA, et al.. TGS-GapCloser: A fast and accurate gap closer for large genomes with low coverage of error-prone long reads. GigaScience. 2020; doi: 10.1093/gigascience/giaa094.

Lines 151-156 - this section would be better located below the assembly methods. Please number supplementary tables in order of their appearance in the text.
Response: Thank you for your suggestion regarding Lines 151-156. We agree that this section would be better positioned below the assembly methods. Additionally, we will ensure that the supplementary tables are numbered in the order they appear in the text. Your feedback is greatly appreciated and will help improve the organization of our manuscript.

Line 171 - please provide parameters used here and for all analyses.
Response: Thank you for your request for additional details on the parameters used. We have added the relevant parameters used for all analyses in the manuscript as follows:
HiFi reads were assembled using Hifiasm (version 0.16.1-r375) with default parameters.
ONT reads were assembled using NextDenovo (version 2.5.0) with default parameters, including genome_size = 2660.49M.
Additionally, more detailed analysis processes and the associated code are publicly available through our GitHub repository: https://github.com/CCyeah/JH-T2T. Thank you for your feedback, which has helped improve the clarity of our manuscript.

Lines 187-188 - how did rearranging contigs decrease the gaps? Was the same gap filling procedure used after HiC manual adjustments?
Response: Thank you for your questions regarding the gap reduction process. Rearranging contigs helped decrease the gaps by optimizing the scaffold structure, allowing for better alignment and integration of contigs. This rearrangement often leads to more accurate placement of sequences, which can effectively close gaps. You can see the detailed process for this step in Figure S3. This figure illustrates how we reduced the gaps by rearranging contigs, we will ensure this is clearly explained in the revised manuscript.

Line 188 - Figure S3 - I don't understand the relationship between the panels nor what the authors are attempting to show. If panels A-C display chromosomes 2, 8, and 13, Why does D display chr3? Both panels C and E are labeled chr13 but they look nothing alike. Are D-E whole chromosomes or zoomed in views? Missing description of panel F.
Response: Thank you for your valuable feedback regarding Figure S3. We have provided a detailed explanation of the relationships between the panels as follows: (A-C) Zoomed-in views Hi-C map of Chromosomes 2, 8, and 13 before correction. Illustrates gaps in Chromosomes 2, 8, and 13 of the JH-T2T assembly that was identified through scaffolding without subsequent editing. (D-E) Hi-C map of Chromosome 3 before correction. Displays Chromosome 3 following the correction of misjoins, with the previously erroneous joins highlighted by red ellipses. Presents Chromosome13 before the misjoins have been rectified, with corrected areas indicated by red ellipses. (F) Hi-C map of Chromosome 13 before correction.

Lines 222-224 - why weren't pig proteins used? Ensembl rapid release has annotated protein datasets for 9 pig assemblies.
Response: Thank you for your question regarding Lines 222-224. We did utilize pig proteins along with those from eleven other closely related species; however, this detail was inadvertently omitted from the text. We will revise the manuscript to clarify our approach and include this important information. Your feedback is greatly appreciated.

Powered by Editorial Manager® and ProduXion Manager® from Aries Systems Corporation

Line 264 - although most will know this, make it clear that Sscrofa11.1 is an assembly of a Duroc pig.
Response: Thank you for your suggestion regarding Line 264. We have made it clear in our new main text that Sscrofa11.1 is an assembly derived from a Duroc pig to ensure clarity for all readers. Your feedback is appreciated.

Line 292 - how was polishing performed? This is missing from the methods.
Response: Thank you for your observation regarding Line 292. The term "polishing" has caused some confusion, and we have deleted it to enhance clarity. Your feedback is valuable for improving the completeness of our manuscript.

Line 294 - should this read "selected it for the backbone of the genome assembly."?
Response: Thank you for your suggestion regarding Line 294. This has been amended to read "selected it for the backbone of the genome assembly," which clarifies the intended meaning. Thank you for your suggestion.

Lines 298-299 - methods?
Response: Thank you for your inquiry regarding Lines 298-299. We have added it to methods that NextPolish2 [1] was used to polish the assembly with default parameters. This clarification will ensure a comprehensive understanding of our methods. Your feedback is appreciated and will help enhance the manuscript.
1. Hu J, Wang Z, Liang F, Liu S-L, Ye K, Wang D-P. NextPolish2: A Repeat-aware Polishing Tool for Genomes Assembled Using HiFi Long Reads. Zhao F, editor. Genomics, Proteomics & Bioinformatics. 2024; doi: 10.1093/gpbjnl/qzad009.

Line 314 - what is meant by "using mapped K-mers from trio Illumina PCR-free reads data"?
Response: In our revision, we included employing a trio-binning approach to generate haplotype resolved assemblies from the two sequenced parental genomes. And we used trio Illumina PCR-free reads data to evaluating assembly quality.

Line 331 - accession numbers for assemblies would be useful.
Response: Thank you for your suggestion regarding Line 331. We have included the accession numbers for the assemblies to enhance the clarity and accessibility of our data. Your feedback is appreciated.

Line 333 - what is "properly mapped rate"? Do you mean properly paired mapping rate?
Response: We will clarify what is meant by "properly mapped rate," using terminology such as "properly paired mapping rate" for precision.

Line 346 - what is the historical genome version?
Response: We recognize that the term could lead to misunderstandings regarding draft assemblies. To avoid any confusion, we will remove this phrase from the manuscript. Your feedback is greatly appreciated and will help improve the clarity of our work.

Line 349 - Supplemental Table S8 only has 55 entries including the 6 remaining gaps. Where are the other filled 8 gaps located?
Response: The other filled 8 gaps were resolved through adjustments made to the Hi-C map to correct misassembles. As a result, these gaps cannot be precisely located within the existing order of the assembly. Thank you for your insightful feedback.

Lines 350-358 - read depth displays wouldn't show the presence of clipped reads which would indicate an improperly closed gap. It would be more convincing to display IGV windows containing these alignments showing that there are no clipped reads.
Response: Thank you for your suggestion. We showed the IGV window containing the results of these comparisons using IGV, which showed some clipped reads, but still some reads in the same region that were not clipped, e.g. at 1:153402833-153420736. We understand that structural differences between individual genomes and references can lead to shear mapping [4]. We prefer not to provide IGV images for all gap regions as it is too much, but we provide the raw data that can be viewed in the corresponding window. Thank you for your suggestion.

Powered by Editorial Manager® and ProduXion Manager® from Aries Systems Corporation

	4. Yang C, Zhou Y, Song Y, Wu D, Zeng Y, Nie L, et al.. The complete and fully-phased diploid genome of a male Han Chinese. Cell Res. 2023; doi: 10.1038/s41422-023-00849-5.<br><br>Line 354 - Figure S5 needs a better legend. What is ref and what is own?<br>Response: Thank you for your feedback regarding Line 354. We will improve the legend for Figure S5 to clearly define "ref" as Sscrofa11.1 and "own" as JH-T2T, providing a more comprehensive explanation of the data presented. Your suggestion is appreciated and will enhance the clarity of the figure.<br><br>Line 359 - the assembly is near-gapless.<br>Response: Thank you for your suggestion. We have state that the assembly is "near-gapless" and provide data supporting the claim of phasing.<br><br>Line 359 - where is the data regarding assembly phasing? How was this determined to be fully phased?<br>Response: Thank you for your question regarding Line 359. We will include the specific data related to assembly phasing and clarify the methods used to determine that the assembly is fully phased. This information will be added to the revised manuscript to enhance transparency and understanding. Your feedback is greatly appreciated.<br><br>Line 363 - 16 of 20 chromosomes are gapless.<br>Response: We have addressed the finding that 16 out of 20 chromosomes are gapless. Thank you for your valuable feedback.<br><br>Line 370 - only 33 telomeres were found at the expected location (end of the chromosome), if you count the telomere on chr2 59kb from the end, then 34 telomeres were identified.<br>Response: Thank you for your observation regarding Line 370. We will clarify that only 33 telomeres were found at the expected locations at the ends of the chromosomes. This clarification will be included in the revised manuscript. Your feedback is appreciated.<br><br>Line 372 - chr13 also only has a single telomere. It does not have a telomere at the beginning.<br>Response: Thank you for your clarification regarding Line 372. We will correct the text to specify that chromosome 13 also has only a single telomere located at the end, and does not have a telomere at the beginning. This revision will ensure accurate representation in the manuscript. Your feedback is appreciated.<br><br>Line 372 - chr19 is chrX correct?<br>Response: Thank you for your question regarding Line 372. Yes, chromosome 19 refers to chromosome X in this context. We will clarify this in the revised manuscript to avoid any confusion. Your feedback is greatly appreciated.<br><br>Line 374 - Figure 1G - It would be nice to have the centromeres marked on this plot (or in Figure 3A). Are the long blocks of telomeric repeats internal to the chromosomes expected?<br>Response: Thank you for your suggestions regarding Line 374. We have marked the centromeres on Figure 1G and/or Figure 3A for better clarity. Additionally, we will address whether the long blocks of telomeric repeats found internally within the chromosomes are expected, providing an explanation in the revised manuscript. These interstitials or pericentromeric telomeric sequences (ITS) have been evidenced as relics of genome rearrangements in some vertebrate’s species. Your feedback is valuable and will help enhance the figures.<br><br>Line 423 - Figure 3A - there is no telomeric repeat at the beginning of chr4 or chrX<br>Response: Thank you for your observation regarding Line 423. We will update the manuscript to clarify that there is no telomeric repeat at the beginning of chromosome 4 or chromosome X as shown in Figure 3A. Your feedback is valuable and will help improve the accuracy of our work.<br><br>Line 431 - why were only 5 pigs of each breed used to validate SVs when 100's of WGS datasets from the two breeds had been aligned? How were these 5 selected?

Response: We provided the genotype dataset for the 905 individual pigs extracted from the PHARP database as SNP annotations on the download site. The remaining 5 pigs of each breed were used in the form of raw data, rather than genotype data. The selection of the 5 pigs from each breed for SV validation was based on this available data. Thank you for your question, which will help improve the clarity of our study.

Line 481 - Sscrofa11.1 only has 544 gaps.
Response: We will clarify that Sscrofa11.1 has 544 gaps in the context of our comparison. This detail will be included in the revised manuscript to ensure clarity. Thank you for your feedback.

Line 492 - ONT data was used to fill more than 6 gaps. Gaps in the assembly were reduced from 63 to 14 using ONT contigs.
Response: We have amended that ONT data was used to fill more than 6 gaps, reducing the total number of gaps in the assembly from 63 to 14 using ONT contigs. This information will be included in the revised manuscript for better clarity. Thank you for your suggestion.

Lines 588-589 - please make your code publicly available through zenodo, github, figshare, or something similar.
Response: We appreciate your suggestion for sharing our code publicly, and we have made our code publicly available through github(https://github.com/CCyeah/JH-T2T).

Line 815-824 - Figure 2 - legend description needs to be improved. Only A is mapping rates, B and C are PM rates and base error rates. The color switch from A-C having European pigs in blue to D having JH-T2T in blue might confuse readers.
Response: Thank you for your feedback regarding Lines 815-824 and the legend for Figure 2. We have improved the legend description to clearly indicate that only panel A represents mapping rates, while panels B and C show PM rates and base error rates, respectively. Additionally, we will address the color switch issue, ensuring consistency in color representation throughout the figure to avoid confusing readers. Your suggestions are greatly appreciated and will enhance the clarity of the figure.

Reviewer #3: A telomere-to-telomere or near gapless genome assembly of the domestic pig (Sus scrofa) will be a valuable resource for researchers working on pigs both in an agricultural context and as a biomedical model. The authors report the production of such a pig genome sequence assembly.
Response: Thank you for your thorough review and constructive feedback. We appreciate your insights and will address each of your comments in the revised manuscript.

Abstract
The abstract states that some of the genes highlighted in the study are associated with domestication. No such evidence is presented in the manuscript and nor is domestication mentioned in the results or discusssion section of the manuscript.
Response: We have revised the abstract to remove related section with domestication.

Introduction
The introductory text cites the relevant literature and sets out the need for, and potential benefits of, the new genome assembly.
Response: We appreciate your positive feedback regarding the literature cited. We will ensure clarity in the need for the new genome assembly.

Methods
The laboratory methods are described briefly, but in sufficient detail to facilitate replication.
Response: Thank you for your comment regarding the Methods section. We aimed to provide a brief overview of the laboratory methods while ensuring there is sufficient detail to facilitate replication of our work. We appreciate your feedback and will consider any specific areas for improvement you may suggest.

Lines 138-139: the tissues sampled need further clarification. For example, surcerebellum and incerebellum are not terms in common usage.

	Response: Thank you for your comment regarding Lines 138-139. We have clarified the terminology used for sampled tissues, specifically replacing "surcerebellum" and "incerebellum" with standard terms "cerebellar cortex" and "cerebellar medulla". Your feedback is valuable and will help improve the accuracy of our work.<br><br>Lines 249-251: thresholds of TPM > 0 and expression in one sample are very lax. In effect no filter has been applied to the expression data and the authors risk reporting expression artefacts.<br>Response: Thank you for your observation regarding Lines 249-251. We used the thresholds of TPM > 0 and expression in one sample to identify expressed genes, as referenced [5]. While we recognize that these thresholds may seem lax, they were chosen based on standard practices in the field. However, we appreciate your concern about the potential for reporting expression artefacts.<br>5. Miao J, Wei X, Cao C, Sun J, Xu Y, Zhang Z, et al.. Pig pangenome graph reveals functional features of non-reference sequences. J Anim Sci Biotechnol. 2024; doi: 10.1186/s40104-023-00984-4.<br><br><br>Results<br>line 293-4: rephrase "we selected it for the bone genome assembly" - this makes no sense in english.<br>Response: This has been amended to read " selected it for the backbone of the genome assembly," clarifying the intended meaning. Thank you for your suggestion.<br><br>Table S1: in the title "assembly" ra...
**Additional Information:**	
**Question**	**Response**
Are you submitting this manuscript to a special series or article collection?	No
**Experimental design and statistics**<br><br><br>Full details of the experimental design and statistical methods used should be given in the Methods section, as detailed in our Minimum Standards Reporting Checklist. Information essential to interpreting the data presented should be made available in the figure legends.<br><br><br>Have you included all the information requested in your manuscript?	Yes
**Resources**<br><br><br>A description of all resources used, including antibodies, cell lines, animals and software tools, with enough information to allow them to be uniquely identified, should be included in the Methods section. Authors are strongly encouraged to cite Research Resource Identifiers (RRIDs) for antibodies, model	Yes

organisms and tools, where possible.<br><br>Have you included the information requested as detailed in our Minimum Standards Reporting Checklist?	
**Availability of data and materials**<br><br>All datasets and code on which the conclusions of the paper rely must be either included in your submission or deposited in publicly available repositories (where available and ethically appropriate), referencing such data using a unique identifier in the references and in the “Availability of Data and Materials” section of your manuscript.<br><br>Have you have met the above requirement as detailed in our Minimum Standards Reporting Checklist?	Yes
GigaScience has policies and guidelines in place for the use of generative AI-writing tools such as ChatGPT. If you have used such writing tools to assist with writing the manuscript this must be declared and cited in the text. Authors should not list AI-writing tools and other AI-assisted technologies as an author or co-author and should acknowledge that they are fully responsible for text generated or refined by AI-writing tools.<p><br><br>A summary of use (particularly in the introduction or among methods) needs to be included at the end of the paper, and the outputs should also be included as a supplementary file hosted in GigaDB or other open repositories. Please read our guidelines for more information. <p><br><br>By submitting to GigaScience, you are aware of the journal's AI-writing tools policy, and if you have declared use of such tools below, you have acknowledged this where appropriate in your manuscript and have made a summary of use and outputs available. <p><br><b>AI-assisted writing tools have been	No

used in the preparation of this manuscript?	

Powered by Editorial Manager® and ProduXion Manager® from Aries Systems Corporation

Manuscript
Click here to access/download;Manuscript;Main text-v2.docx

The first near-complete genome assembly of pig: enabling more accurate genetic research

Caiyun Cao[1,2], Jian Miao[1], Qinqin Xie[1], Jiabao Sun[1], Hong Cheng[1], Zhenyang Zhang[1], Fen Wu[1], Shuang Liu[1], Xiaowei Ye[1], Huanfa Gong[1], Zhe Zhang[1], Qishan Wang[1,2], Yuchun Pan[1,2,*], Zhen Wang[1,*]

[1]College of Animal Sciences, Zhejiang University, Hangzhou, Zhejiang 310058, China.

[2]Hainan Institute of Zhejiang University, Building 11, Yongyou Industrial Park, Yazhou Bay Science and Technology City, Yazhou District, Sanya, 572025, Hainan, China.

*Corresponding authors:

Zhen Wang: wangzhen20@zju.edu.cn; Yuchun Pan: panyc@zju.edu.cn

E-mail addresses:

CC: ccyun@zju.edu.cn

JM: miaojian6363@163.com

QX: qinqin.xie@zju.edu.cn

JS: sunjiabao@zju.edu.cn

HC: chengh7619@163.com

ZZ: zhangzy1995@aliyun.com

FW: 18805815950@163.com

SL: liushuang9917@zju.edu.cn

XY: ye_xw@zju.edu.cn

HG: gonghuanfa@zju.edu.cn

QW: wangqishan@zju.edu.cn

ZZ: zhe_zhang@zju.edu.cn

ZW: wangzhen20@zju.edu.cn

YP: panyuchun1963@aliyun.com

Abstract

Background

Pigs are crucial sources of meat and protein, valuable animal models, and potential donors for xenotransplantation. However, the existing reference genome for pigs is incomplete, with thousands of segments and centromeres and telomeres missing, which limits our understanding of the important traits in these genomic regions.

Findings

We present a near complete genome assembly for the Jinhua pig (JH-T2T) and provides a set of diploid JH reference genome, constructed using PacBio HiFi, ONT long reads and Hi-C reads. This assembly includes all 18 autosomes and the X and Y sex chromosomes, with only six gaps. It features annotations of 46.90% repetitive sequences, 33 telomeres, 17 centromeres, and 23,924 high-confident genes. Compared to the Sscrofa11.1, JH-T2T closes nearly all gaps, extends sequences by 177 Mb, predicts more intact telomeres and centromeres, and gains 799 more genes and loses 114 genes. Moreover, it enhances the mapping rate for both Western and Chinese local pigs, outperforming Sscrofa11.1 as a reference genome. Additionally, this comprehensive genome assembly will facilitate large-scale variant detection.

Conclusions

This study produced a gapless and near-gapless assembly of the pig genome, and provides a set of diploid JH reference genome. Our findings represent a significant advancement in pig genomics, providing a robust resource that enhances genetic research, breeding programs, and biomedical applications.

Keywords: Pig genome assembly, HiFi and ONT sequencing, gapless reference genome

Data Description

Background information

Pig (*Sus scrofa*) is not only economically important due to its role as a food source but also serves as a medical model and potential xenotransplantation donor because of its anatomical and physiological similarities with humans [1,2]. Understanding the genome and gene content of candidate species, including pigs, is crucial for selecting the best animal model species for pharmacological or toxicological studies. High-quality, fully annotated genome sequences are essential for gene editing, producing improved animal models for research, or providing cells and tissues for xenotransplantation, as well as enhancing productivity [3,4].

Despite the availability of several high-quality pig reference genomes, including those of the European Duroc [5], Ninxiang [6], Meishan [7], and Jinhua [8,9] pig genomes, these assemblies remain incomplete in genomic regions of repetitive sequences, centromeres, and telomeres [5–9]. A gap-free genome is the ultimate goal of genome assembly, crucial for improving the accuracy of read mapping and variant calling for individuals sequenced with short and long reads [10], and offers new opportunities for identifying unique genes and structural variations (SVs) [11,12]. However, to date, a gapless pig reference genome has not yet been reported.

Advancements in new sequencing technologies and computational algorithms have ushered in the era of telomere-to-telomere (T2T) assemblies [13]. Specifically, third-generation sequencing technologies, which generate long reads enabling whole-

genome assembly, have improved both experimental methods and algorithms. For example, Pacific Biosciences (PacBio) methods can generate ~10 Kb long HiFi reads with 99% accuracy, while Oxford Nanopore Technologies (ONT) recently developed an ultra-long read method producing reads with an average length of ~50 Kb, extending up to ~100 Kb, with the longest reads reaching hundreds of Kb [14–16]. HiFi reads can assist in assembling complex genomic regions[17], while the ONT ultra-long reads can help assemble genomic regions with tandem duplications[18]. The application of third-generation sequencing and assembly technologies to high-fidelity long reads will contributed to the creation of gap-free genome assemblies across hundreds of species [19].

Therefore, we assembled a nearly gap-free T2T genome of the Jinhua pig -one of China's four renowned indigenous breeds, famous for its superior meat quality and high-quality Jinhua-ham [20] using PacBio HiFi and ONT long reads. This T2T genome assembly marks a significant advancement in pig genomics. It offers enhanced resources for research in pig genetics, genomics, and biomedical applications. This assembly overcomes the limitations of previous incomplete assemblies, serving as a robust platform for various downstream comparative genomic analyses and providing new insights into the complex traits of pigs.

Materials and methods

Sample collection

Fresh blood was collected from a healthy male Jinhua pig at the National Jinhua Pig Conservation Farm in Zhejiang, China, in 2022 **(Figure 1A and Figure S2H)**. Ear tissue samples were collected from its parents.

DNA extraction, library construction, and sequencing

DNA extraction. High-molecular weight DNA was extracted using the cetyltrimethylammonium bromide (CTAB) method and purified with the QIAGEN Genomic Kit (Catalog No. 13343, QIAGEN, Hilden, Germany). Ultra-long DNA was extracted using the sodium dodecyl sulfate (SDS) method [21], omitting the purification step to maintain DNA length. DNA purity was assessed using a NanoDrop One UV-Vis spectrophotometer (Thermo Fisher Scientific). DNA degradation and contamination were monitored on 1% agarose gels. DNA concentration was measured with a Qubit 4.0 fluorometer (Thermo Fisher Scientific).

PacBio library preparation and sequencing. SMRTbell target-size libraries were prepared according to PacBio's standard protocol (Pacific Biosciences, CA) using 15-18 Kb preparation solutions. The main steps included: (1) DNA shearing: high-quality DNA samples (primary band >30 Kb) were selected and randomly fragmented into15-18 Kb pieces using the g-TUBE (Covaris, MA); (2) DNA damage repair, end repair, and A-tailing; (3) Blunt-End ligation: hairpin adapters from SMRTbell Express Template Prep Kit 2.0 (Pacific Biosciences) were ligated; (4) Template purification: imperfect SMRTbell templates were removed with EXOIII (from 3'-hydroxyl termini and nicks) and VII (from 5'-termini) treatment; (5) Size selection: performed using the bluePippin system. Next, the AMPure PB beads were used to concentrate and purify the templates. Then, the sequencing was performed on a PacBio Sequel II instrument with Sequencing Primer V2 and Sequel II Binding Kit 2.0 at Novogene Co., Ltd (Beijing, China).

ONT library preparation and sequencing. Libraries were prepared using the SQK-LSK110 ligation kit following the standard protocol. The purified library was loaded onto primed R9.4 Spot-On Flow Cells and sequenced using a PromethION sequencer (Oxford Nanopore Technologies, Oxford, UK) with 48-h runs at Wuhan Benagen Technology Co., Ltd (Wuhan, China). Base calling of raw data was performed using the Oxford Nanopore GUPPY software (v0.3.0).

Hi-C library preparation and sequencing. For Hi-C sequencing, purified DNA was digested with 100 U DpnII and incubated with Biotin-14-dATP. The ligated DNA was sheared into fragments of 300–600 bp, blunt-end repaired, and A-tailed, followed by purification through biotin–streptavidin-mediated pulldown. The Hi-C libraries were quantified and sequenced using the Illumina NovaSeq/MGI-2000 platform.

*Whole-genome re-sequencing***.** For whole-genome re-sequencing, total genomic DNA was isolated from fresh blood using the CTAB method. A 150-bp paired-end library with insert sizes of 350 bp was constructed for each individual following standard Illumina library preparation protocols (Illumina). Meanwhile, PCR-free libraries were prepared with the Illumina TruSeq DNA PCR-free library prep kit (Illumina) according to the manufacturer's instructions. The qualified libraries were then sequenced using an Illumina Hi Seq X Ten platform to produce 150-bp paired-end reads. RNA extraction, library construction, and sequencing.

For RNA-seq, 19 samples collected form 19 different tissues (hypo, midbrain, hypophysis, cerebellar cortex, cerebellar medulla, amygdala, pineal, occipital, hippocampus, striatum, parietal, frontal, temporal, muscle, jejunum, ileum, caecum, colon and duodenum) in one JH pig. Total RNA was isolated using the RNAprep Pure

Plant Kit (TIANGEN, Beijing, China). All tissues' total RNA was prepared for mRNA sequencing by using the TRizol reagent. RNA integrity and yield were assessed by the RNA Nano 6000 Assay Kit of the Bioanalyzer 2100 system (Agilent Technologies, Santa Clara, CA, United States) and the NanoPhotometer spectrophotometer (IMPLEN, Westlake Village, CA, United States). For each sample, 3 μg of RNA was used to create sequencing libraries using the NEBNext Ultra TM RNA Library Prep Kit for Illumina (NEB, Ipswich, MA, United States) following the manufacturer's instructions. Index numbers were added to identify each sample's sequences. Finally, the clustered libraries were sequenced on an Illumina HiSeq platform, generating 150-bp paired-end reads.

Genome size estimation

To estimate the pig genome size and address potential issues such as sister chromatid merging and repetitive sequences, we used k-mer analysis with the jellyfish software (version 2.2.10) [22]. The command “Jellyfish count -G 2 -m 17 -C” and “histo kmercount” were used to calculate the k-mer count and generate histograms, respectively.

Genome assembly

The main goal of this study was to create a high-quality, gapless assembly of the Jinhua pig, comprising 18 autosomes and two sex chromosomes (X and Y) and assemble the autosomes of the haplotype-resolved genomes (JH.mat and JH.pat). The assembly process followed the Vertebrate Genomes Project (VGP) assembly pipeline [23] with modifications (Figure 1A and Figure S1). First, the initial assembly was

constructed using PacBio HiFi reads and ONT ultra-long reads. For the PacBio assemblies, consensus reads (HiFi reads) were generated using CCS software (https://github.com/PacificBiosciences/ccs) with the default parameter. HiFi reads were then assembled using Hifiasm (version 0.16.1-r375) with default parameters [15,24]. ONT reads were assembled using NextDenovo [25] (version 2.5.0) with default parameters genome_size = 2660.49 M. Second, an auxiliary assembly was performed using Allhic [26] and juicebox [27] to improve the Hifiasm output assembly with the help of Hi-C reads. Allhic was utilized to assign the assembled contigs/scaffolds to near-chromosome level. The chromosome interaction intensity, based on the juicebox software, was used for manual correction. NextPolish2 [28] was used to polish the assembly with with the default parameter. The initial assembly for the autosomes of the haplotype-resolved genomes was performed by using hifiasm(v0.16.1) [15] and verkko(v1.1) [29] based on the trio mode with HiFi reads, ultra-long ONT reads and and the parents' short reads.

Gap filling

To fill the gaps in the genome assembly, we used the winnowmap (v1.11) software with parameters (k=15, -MD) [25]. This process involved comparing the hole-filling data (error-corrected ONT genome versions, HiFi or ONT reads) with the genomic gap intervals. The priority for gap filling steps was given first to error-corrected genome versions, followed by ONT and HiFi reads. Using this approach, we reduced the number of gaps from 63 to 14. The remaining 14 gap regions could not be adequately covered by the assembly/ONT/HiFi data due to a lack of good reads. We then mapped these gap regions with Hi-C data, generated Hi-C interactions, and

imported them into juicebox. After identifying mapping errors, we made manual adjustments, resulting in a final JH-T2T genome with only six gaps **(Figure S3)**.

Datasets and their sources

Genotypes from 938 individuals were collected from PHARP database [30] **(Supplementary Table S7).** Additionally, 92 RNA-seq data from ten pig population, covering eleven different tissues (brain, heart, liver, spleen, lungs, kidneys, fat, muscle, ovaries, testicles, and intestinal segments) were downloaded from NCBI **(Supplementary Table S8)**.

Genome assembly quality assessment

To systematically evaluate the quality of the genome assembly, we conducted the following assessment: i) Gene completion. The gene completion of the assembly was evaluated using BUSCO (v5.4.3) with the mammalia_odb10 dataset [25]. ii) Genome continuity. The genome continuity was assessed by calculating contig N50 length using QUAST (v5.0.2) [32]. iii) Quality value (QV). Merqury [33] was used to calculate QV combining Illumina reads. iv) Reads mapping rate and coverage. We mapped the WGS (n=153), and HiFi (n=1) and ONT (n=1) reads to the assembly using BWA-MEM2 and minimap2 [34], respectively. We then calculated their mapping rates and coverages.

Identification of telomeres and centromeres

In vertebrates, telomeres consist of conserved repetitive sequences as described in the Telomere Database (http://telomerase.asu.edu/sequences_telomere.html). Here, we

also used the vertebrate telomeric repeat (6-mer TTAGGG/CCCTAA) to identify telomeres using the Tidk (v0.2.0) tool [35] and Seqtk (v1.4) telo module (https://github.com/lh3/seqtk). Tidk detected telomeric repeat sequences throughout all the sequences, the final telomere identification results are based on the Seqtk telo findings. Centromics software (https://github.com/ShuaiNIEgithub/Centromics) was used to pinpoint centromere regions. This tool utilizes characteristics such as a high density of short tandem repeats and a low density of genes, which are typical of centromere regions, to identify centromeres in the JH-T2T genome.

Repeat annotation

The homologous repeat annotation library for the JH-T2T genome was constructed by extracting mammalian repeat sequences from a combined library comprising Repbase (release 20181026) and Dfam (version 3.2)[36,37]. RepeatModeler (version 2.0.3)[38] was then used to analyze and predict repeat sequences based on this library. Finally, the Repeatmasker (version 4.1.2)[39] was employed to annotate the transposable elements (TEs) in the JH-T2T genome using the custom non-redundant set of repeats.

Gene annotation

To annotate the protein-coding genes in the JH-T2T genome, a combination of ab initio, homology-based, and transcriptome-based prediction methods were employed. For the ab initio gene prediction, the MAKER3 pipeline [40] was applied to predict gene structures in the masked JH-T2T genome. High-quality protein sequences from Ensembl release 106 were used for gene annotation, including pigs and eleven closely

related species (*Homo sapiens, Equus caballus, Canis lupus, Bos_taurus, Capra_hircus, Ovis_aries, Camelus dromedaries, Delphinapterus leucas, Balaenoptera musculus, Physeter catodon, and Tursiops truncates*). Additionally, transcripts from 111 samples (**Supplementary Table S8**) generated from our RNA-Seq data and public available data were processed using HISAT2 (v2.2.1) and StringTie (v2.1.4) [41,42]. The initial round of gene annotation utilized protein sequences and transcripts. BLASTN [43] with an e-value cutoff of 1e-10 was used to map these homologous protein sequences to the JH-T2T genome. Only the protein sequences with the highest-scoring alignments, having a minimum identity score greater than 80%, were retained to predict putative gene models using Exonerate (v2.4.0) [44]. The second round transcript-based gene prediction involved training SNAP (v2006-07-28) [45] and AUGUSTUS (v3.4.0) [46] with predicted gene models to predict genes.

Functional annotation of protein-coding genes

We employed three methods to annotate functions of protein-coding genes. First, protein sequences similarity were searched against the NCBI nonredundant protein database and the Swiss-Prot database [47,48] using BlastP software [43]. Second, protein domain and gene ontology term annotations were performed using InterProScan [49]. Third, KEGG annotation was performed with the kofam_scan [50]. These methods provide complementary approaches, combining sequence similarity, domain analysis, and pathway information to gain insights into the potential functions of these genes in the JH-T2T genome. Additionally, the expression of these genes was also examined using the RNA-seq data. We first used fastp [51] to remove the low-

quality reads and adapters in the raw RNA-seq reads, and mapped the remaining reads to the transcripts of high-quality predicted genes by Hisat2 [41]. We then used StringTie (v.2.1.7) [42] to assemble and quantify transcripts guided by the JH-T2T genome. The transcripts were evaluated based on transcripts per million (TPM) values. A TPM > 0 indicated the presence of a transcript in a sample. If a transcript occurred in at least one sample, it was considered as validated, indicating the expression of the predicted gene.

Global comparison of the Sscrofa11.1 and JH-T2T genome

To assess variation in chromosome-scale synteny, we compared the JH-T2T and Sscrofa11.1[5] assemblies. We began by aligning the two genomes using NUCmer [52] with parameters -l 100 -c 1000, refining the results with Delta-filter using parameters -i 95 -l 100 -1. Additionally, Minimap2 [34] with parameters -cx asm5 -t8 --cs was used to align Sscrofa11.1 to JH-T2T. The optimal alignments were used for SNPs and indels calling with paftools.js [34]. For detecting structural variants (SVs), we used Minimap2 with parameters -a -x asm5 --cs -r2k to to get the best alignments, followed by SV calling with svim-asm using the haploid parameter [53]. To explore the functional implications of deleterious variants, we selected genes with such variants for enrichment analysis using KOBAS [54]. Next, we employed Liftoff (version 1.6.2) [55] to map genes between Sscrofa11.1 and JH-T2T, assessing their consistency. Since Sscrofa 11.1 is an ensemble from Duroc pigs, we aligned WGS clean reads including JH and Duroc pigs to both assemblies using BWA-MEM tool with default parameters [56] to examine the coverage and depth of detected SVs. These analyses allowed us to assess variation in chromosome-scale synteny, identify

genetic variants, investigate missing genes, and validate SVs in the JH-T2T and Sscrofa11.1.

Selection signatures between JH and Duroc pigs in large SV regions

To examine selection signatures in large SV regions between Jinhua and Duroc pigs, we analyzed genotypes from 289 Jinhua and 616 Duroc pigs **(Supplementary Table S7)**. We used three approaches to detect selection signals: fixation index (FST), nucleotide diversity ratio ($\theta\pi$), and cross-population extended haplotype homozygosity (XP-EHH). The FST and $\theta\pi$ were calculated across the genome using 10 Kb non-overlapping sliding windows with VCFtools (v0.1.16) [57]. The XP-EHH was conducted with selscan (v1.2.0) [58], averaging XP-EHH scores over 10 Kb non-overlapping sliding windows. Genomic regions in the top 5% values for at least one selection signature were identified as selective sweeps. Genes in these selective sweep regions were considered candidate high-related genes.

Results

T2T assembly of JH pig genome

We generated a total of 51.10× sequence coverage of raw PacBio HiFi data (135.95 Gb, read N50 18.32 Kb), 136.65× sequence coverage of ultralong ONT data (363.55 Gb, read N50 52.17 Kb), 94× sequence coverage of Hi-C data, and 50× sequence coverage of WGS data for assembling the JH pig genome (**Figure S2A-D** and **Supplementary Table S1**). Using the HiFi reads, we assembled the initial PacBio HiFi assembly, which had a total length of 2.72 Gb and consisting 187 contigs (contig N50 84.83 Mb, **Figure S2E**). The initial ONT assembly had a total length of 2.28 Gb

and consisting 93 contigs (contig N50 64.34 Mb, **Figure 1A, Figure S2F,** and **Supplementary Table S2**). A second ONT assembly using only the longest ONT reads (87.23G, read N50 100 Kb) had a total length of 2.31 Gb and consisting 112 contigs (contig N50 72.01 Mb, **Figure S2G**), which were used to fill the gaps. Since the PacBio HiFi assembly showed a higher quality and contiguity compared to the ONT assembly, we selected it for the backbone of the genome assembly. We used Hi-C data to order and orient these PacBio HiFi contigs, resulting in 20 chromosomes (with six gaps, scaffold N50 142.74 Mb) representing chromosomes 1-18, X, Y, and 66 unplaced contigs containing an additional 62.32 Mb (**Figure 1A-B, Figure S2E,** and **Supplementary Table S3)**. The PacBio HiFi assembly was further iteratively polished by PacBio HiFi reads, ONT reads, Hi-C data (for mapping error correction), and the second ONT assembly, resulting in a near-T2T assembly with a total length of 2.68 Gb (2.61 Gb mounted on the chromosome, mounting rate of 97.67%, contig N50 142.75 Mb) and only six gaps remaining in chromosomes 2, 3, 8, and 10 (**Figure S2E** and **Supplementary Table S4**).

The initial hifiasm haplotype assemblies had total length of 2.68 Gb (275 contigs and contig N50 106.25 Mb), 2.33 Gb (137 contigs and contig N50 80.18 Mb), respectively. The initial verkko haplotype assemblies had total length of 2.36 Gb (276 contigs and contig N50 30.78 Mb), 2.17 Gb (252 contigs and contig N50 25.26 Mb), respectively **(Supplementary Table S2)**. The more continuous contigs were selected from the two assemblies to represent. This results in a maternal assembly with 157 gaps and paternal assembly with 99 gaps. Subsequently, gap closing was performed using TGS-Gapcloser [59] with the verkko assembly, resulting 116 and 42 gaps,

respectively. The final diploid JH reference genome has NG50 of 147.60 Mb and 143.06 Mb for maternal and paternal genomes, respectively.

Quality assessment of the final JH-T2T assembly

We conducted a comprehensive assessment of the JH-T2T assembly's quality and completeness in multiple ways. First, the estimated genome size was determined to be 2.69 Gb, with a heterozygosity rate of 0.38%, consistent with the Sscrofa11.1 genome size (**Figure S5A** and **Supplementary Table S4**). Second, 16 of the 20 chromosomes were each represented by a single contig (**Supplementary Table S4**), indicating superior sequence integrity compared to the current pig reference genome, Sscrofa11.1 (1,117 contigs), and other published pig genomes (**Figure 1C**, **Table 1**, and **Supplementary Table S5)**. Third, the JH-T2T assembly showed high overall base accuracy, estimated at 99.997% (an average QV score of 55) using mapped K-mers from trio Illumina PCR-free reads data. QV scores ranged from 48 to 62 for each chromosome, with five chromosomes (chr4, chr9, chr11, chr15 and chr16) having high QV scores greater than 60 (**Figure S5C-D** and **Supplementary Table S4**). Forth, compared to the other three genomes, BUSCO analysis revealed that the JH-T2T assembly exhibited the highest percentage of completeness, with approximately 96.4% of the core conserved mammalian genes being fully represented (**Figure 1F, Figure S5B** and **Supplementary Table S6**). This indicates a near-complete genome assembly. Fifth, the chromosomal interaction maps generated using Hi-C data provided further evidence of the accuracy and reliability of the JH-T2T assembly. Hi-C data revealed that all chromosomes displayed clear intra-chromosomal diagonal signals, with no significant inter-chromosomal signals, confirming the correct order

and orientation of all pseudomolecules (**Figure 1B**). Sixth, the remapping rates for HiFi reads, ONT reads, and Illumina short reads on JH-T2T assembly were impressively high at 99.90%, 99.99%, and 99.99%, respectively.

For alignment-based comparison with other reported genomes, we firstly utilized WGS data from 30 individuals (depth ranging from 10.00 to 27.14×, **Supplementary Table S7**), which were mapped to the Sscrofa11.1 (GCA_000003025.6) [5], MS (ASM1795798v1) [7], NX (ASM2056790v1) [6], and JH-T2T genomes. The JH-T2T showed significantly higher mapping rates ranging (98.65% to 99.87%, **Figure 2A**), properly paired mapped rate (92.16% to 98.51%, **Figure 2B**) and lower Base error rates (0.64% to 1.62%, **Figure 2C**) compared to other genomes. The average mapping rate for Asian pigs was 99.53% on JH-T2T versus 97.98% on Sscrofa11.1, and for European pigs, 99.48% versus 98.44% (**Figure 2A**). The average properly mapped rate for Asian pigs was 97.84% on JH-T2T versus 94.68% on Sscrofa11.1, and for European pigs, 94.78% versus 93.04% (**Figure 2B**). Next, mapping 111 RNA-seq data (**Supplementary Table S8**) from Asian (n=61) and European (n=50) pig breeds showed that JH-T2T was more suitable for analyzing RNA-seq data from Asian pig breeds, with higher mapping rates (88.70%) compared to Sscrofa11.1 (87.67%, **Figure 2D**). The average mapping rate of European pigs on JH-T2T and Sscrofa11.1 was similar (89.45% versus 89.58%, **Figure 2D**). The above results suggest that JH-T2T will be advantageous for both DNA and RNA sequencing data mapping analysis.

Additionally, 49 out of 63 gaps were successfully closed and 8 out of 63 gaps were corrected in our final JH assembly, with filled gaps ranged from 81 to 35,183 bp, totaling around 268 Kb (**Figure S3, S6** and **Supplementary Table S9**). We remapped

ONT and HiFi reads to the post-gap filled genome to confirm the reliability for each filled gap. Most filled gaps were identifiable through ONT or HiFi alignments, and assembly errors in low-coverage regions (LCRs) were corrected via ONT alignments (**Figure 2E** and **Figure S7**). Specifically, the largest two gaps (35 and 24 Kb) on chromosome 8 were successfully confirmed by coverage with multiple ONT or HiFi reads (**Figure 2I** and **Figure S6-7**). The gaps on chromosomes 1, 2 and 10 were also successfully filled, evidenced by fully coverage with both ONT and HiFi reads (**Figure 2F-H** and **Supplementary Table S9**). These findings confirmed the accuracy and reliability of our JH-T2T assembly.

The quality values (QV) of JH.mat and JH.pat are 56.73 and 60.07. The even coverage distribution of ONT and PacBio HiFi reads suggest ed three reliable and continuous assemblies **(Figure S4A-B)**. Further, by comparing the linear genomes of two complete haplotypes, we detected ~7.23 million single nucleotide variants (SNVs), 1,165,610 small insertions or deletions (indels) (< 50 bp), and 26,701 SVs (⩾ 50 bp).

Overall, our assembly quality metrics indicate a gapless and near-gapless assembly of the pig genome, and provides a set of diploid JH reference genome. To the best of our knowledge, this assembly is the first T2T and the most complete pig genome assembly published.

Genome annotation

The JH-T2T genome assembly provides a gapless T2T sequence for all 16 out of 20 chromosomes, marking significant progress over previous incomplete pig genome

assemblies [5–7]. About 46.90% of the JH-T2T genome consists of repetitive sequences elements: 24.63% LINEs (long interspersed nuclear elements), 3.40% SINEs (short interspersed nuclear elements), 5.23% LTR (long terminal repeat), 2.44% DNA transposons, 1.13% simple repeats, and 5.21% satellites (Supplemental Table **9** and **Figure 1E**).

Using the six-base telomere repeats (TTAGGG/CCCTAA) as a query, 33 out of the anticipated 40 telomeres were identified, with a single telomere detected on chromosomes 4, 11, 13, 15, 18, and X. The average telomere length is 17.95 Kb, with approximately 2,039 repeat copies per telomere. The longest telomere spanned 19.99 Kb (**Figure 1G** and **Supplementary Table S4**). Putative centromeres were identified in expected locations on chromosomes 1–12, 14, and 16-17 (**Figure 3A, Figure S8** and **Supplementary Table S4**). We observed that a few chromosomes exhibit a high copy number of telomere repeats. These interstitial or pericentromeric telomeric sequences (ITS) have been evidenced as relics of genome rearrangements in some vertebrates species at early research[60].

Gene annotation of the masked JH-T2T genome was performed using MAKER [40] with evidence from protein homologies and RNA-seq data. A total of 23,924 high-confidence protein-coding genes were predicted (**Figure 1F**), which includes 799 newly anchored genes (**Supplementary Table S11**). To validate these predictions, RNA-seq data from 111 samples showed that 20,110 (90.50%) of the high-confidence genes were expressed in at least one sample (**Figure 1F**). Gene and repeat distribution across chromosomes follow the typical pattern observed in vertebrate genomes, with higher gene concentrations in GC-rich regions and decreased gene density in repeat-

rich distal regions (**Figure S2K**). Also, the density of genes at telomeres is lower (**Figure 1G**).

Global comparison between the Sscrofa11.1 and JH-T2T genome

The JH-T2T genome assembly showcases greater completeness and accuracy compared to the Sscrofa11.1 assembly. First, the JH-T2T assembly added approximately 171 Mb (6.8%) to the Sscrofa11.1 assembly. Second, the completeness measured by BUSCOs, the JH-T2T achieved 96.4% of 9,226 BUSCOs, surpassing Sscrofa11.1's 94.1% (**Figure S5B** and **Supplementary Table S6**). Third, the JH-T2T assembly identified 35 telomeres (out of an expected 40, **Supplementary Table S4**), whereas Sscrofa11.1 captured telomere only at the proximal ends of Sscrofa11.1 chromosome assemblies of SSC2, SSC3, SSC6, SSC8, SSC9, SSC14, SSC15, SSC18, and SSCX. The JH-T2T assembly 17 centromeres on chromosomes 1–12, 14, and 16-17 **(Supplementary Table S4**). Putative centromeres were identified in the expected locations in the Sscrofa11.1 chromosome assemblies for SSC1–7, SSC9, SSC13, and SSC18. Two regions harboring centromeric repeats were identified in the chromosome assemblies of each of SSC8, SSC11, and SSC15. Compared to Sscrofa11.1, JH-T2T predicted a greater number of more intact telomeres and centromeres[5].These enhancements highlight the JH-T2T assembly superior quality and utility for genomic research.

By lifting over genes between JH-T2T and Sscrofa11.1, JH-T2T includes 799 newly anchored genes (**Supplementary Table S11**) involved in 96 KEGG entries, enriching two KEGG pathways and 19 GO terms (**Figure S9B** and **Supplementary Table S12**), notably in olfactory (e.g., olfactory transduction) and immunity-related

pathways (e.g., cytokine-cytokine receptor interaction, Fc gamma R-mediated phagocytosis, and allergies and autoimmune diseases pathway). Moreover, JH-T2T lost 114 genes (**Supplementary Table 11**), significantly enriching five KEGG pathways and 14 GO terms, including steroid hormone biosynthesis and linoleic acid metabolism (**Supplementary Table S12**).

A comprehensive comparison between the JH-T2T and Sscrofa11.1 has identified 58,200 SVs (28,843 deletions and 29,357 insertions, total genome size of 41.5 Mb, ranged from 50 to 144,010 bp) using Sscrofa11.1 as a reference, with 57,796 medium (50–10,000 bp) and 404 large SVs (≥10 Kb) **(Figure S9C and Supplementary Table S13**). More structural variants were identified in Jinhua pigs than in Ningxiang and Meishan pigs **(Figure S9D**). SVs distribution across chromosomes follow the pattern with higher SVs concentrations in repeat-rich regions (**Figure 3B**). The majority of the SVs (71.23%) is located in repeat regions, suggesting that repeat sequences are an important source of genetic diversity in pigs. These repeats effectively filled nearly all genome gaps, including the telomeres (**Figure 3A** and **Figure S8**). The majority of these SVs located in intergenic regions (24.08%) and introns (74.65%), with a minority located within coding sequences (CDS) regions (0.24%) (**Figure 3C)**. Moreover, 12,129 genes were overlapped with these SVs (**Supplementary Table S13**). Using the pig QTL database, we found SVs enriched in 65 QTLs associated with six economic traits, such as basophil number, drip loss, and head weight (*P-value* <0.01, **Figure S9A** and **Supplementary Table S14**), suggesting SVs potentially impact on important economic traits.

Additionally, we simply validated the detected SVs by examining their sequence coverage using the WGS data from five JH pigs and five Duroc pigs. Employing the JH-T2T and Sscrofa11.1 as refence and applying a validation criterion that required SV mapping sequence coverage is 1.00 in one sample and less than 0.90 in another sample, we confirmed a total of 38,021 SVs (approximal 65.32%), comprising 16,240 DELs and 21,781 INSs, which are associated with 13,967 genes (**Supplementary Table 15**). Among these, SVs with a length larger than 500 bp were the least frequent (**Figure 3D)**, highlighting the limitations of SV detection through next-generation sequencing data.

Large-scale genomic differences in JH-T2T genome

In our study, we identified 386 large SVs (≥ 10 Kb) in the JH-T2T genome compared to Sscrofa11.1, including 236 DELs and 150 INSs **(Supplementary Tables S17)**. These SVs affected the presence or absence of 212 genes between the two genomes. Notably, 101 insertions in the Sscrofa11.1 genome contained an additional 100 genes (**Supplementary Table S16**). The majority of these genes are olfactory receptor genes, being significantly enriched in olfactory transduction (including previously reported pig olfactory transduction genes such as *OR8S1* and novel genes like *OR8B3, OR2V2,* and *OR7A17* (**Supplementary Table S17**).

The large SVs also harbored genes related to important economic traits. For example, the *CYP2C18* gene, linked to elevated backfat skatole levels in commercial pig populations [61], was located in the largest SV (~144.0 Kb) on chromosome 14 of JH-T2T, which was located in the selective sweep (**Figure S10A-C** and **Supplementary Table S17**). Similarly, an insertion (~22.2 Kb) in the *GPAM* gene, a marker for

intramuscular fat content (IMF) content in musculus longissimus dorsi (MLD)[62] were observed (**Figure S10A** and **B** and **Supplementary Table S18**). The large SVs also contained genes related to immune response, such as *LY9*, *ITLN2*, and *CHIA* (**Supplementary Table S17**). The *LY9* gene region indicated positive selection in DU pigs (**Figure S11B**), associated with immune response regulation [63]. The *ITLN2* and *CHIA* gene reported to link to asthma susceptibility in humans[64]. Those findings may be linked to asthma susceptibility of JH pig. An insertion (~15.03 Kb) in the *SLA-DOB* gene (**Supplementary Table S16**), which is serve to immune system's response and relevant to transplant rejection [65].

Discussion

In this study, we built the first T2T pig genome assembly, marking a significant milestone in pig genomics. Our JH-T2T genome assembly demonstrated remarkable improvements over existing assemblies [5–8], both in terms of completeness and quality. Notably, this T2T genome assembly left only six gaps in chromosomes 2, 3, 8, and 10, exceeding the minimum quality standards set by the Vertebrate Genomes Project (VGP) consortium [19].

The high quality of the JH-T2T assembly is evident in its ability to capture complex genomic regions, including repetitive sequences, and telomeres, which were previously inaccessible. This comprehensive coverage addresses the limitations of earlier reference genomes, such as Sscrofa11.1, which contained 544 of gaps and lacked repetitive regions, centromeres, and telomeres. By incorporating these regions, the JH-T2T genome provides a more complete and accurate pig reference genome, essential for detailed genetic studies and breeding programs. Similarly, in human, the

use of the T2T-CHM13 genome assembly yields a more comprehensive view of SVs genome-wide, with a greatly improved balance of insertions and deletions [66].

Advancements in sequencing technology, especially the ONT ultra-long sequencing method, have greatly facilitated the complete assembly of genome. The ONT data played a crucial role in filling gaps, particularly in difficult genomic regions such as repetitive regions, centromeres, and telomeres. Many reference genomes have been successively assembled using ONT reads in farm animals, such as cattle [67], chicken [68] and sheep [69]. In our JH-T2T assembly, Gaps in the assembly were reduced from 63 to 14 using ONT contigs.

A key advantage of the T2T genome is its superior performance in improving reference genome mapping. The JH-T2T assembly outperforms Sscrofa11.1 in mapping reads from both Western and Chinese pig populations, minimizing gaps and enhancing read alignment accuracy for both DNA and RNA sequencing data. This improvement is crucial for large-scale variant calling from second- and third-generation sequencing data and functional genomics studies, enabling more precise identification of genetic variants and their associated traits. For example, in human, the T2T-CHM13 assembly was shown to improve the analysis of global genetic diversity based on 3,202 short read-length samples from the 1KGP dataset [66].

Compared to Sscrofa11.1, the JH-T2T genome captures a more comprehensive set of genetic elements. This includes the identification of 799 newly anchored genes not present in Sscrofa11.1, as well as the recognition of 114 genes that were lost in the JH-T2T. This comprehensive capture is made possible by the JH-T2T genome's ability to fill in gaps and cover repetitive regions, centromeres, and telomeres, which

were previously inaccessible. The identification of these novel and lost genes has significant implications for understanding key biological functions, particularly in olfactory function, metabolism, and immune response. Olfactory genes play a critical role in the sensory perception of smell, which is important for behaviors related to feeding, mating, and environmental interaction [70,71].

The comprehensive comparison between the JH-T2T and Sscrofa11.1 has identified 58,200 SVs. Considering that some of the SVs may be due to incomplete genome assembly of Sscrofa11.1, we validated them with WGS data. SVs with lengths (>500 bp) were the least frequent (approximal 65.32%) of validated SVs which may be due to the limited sample size of the WGS data, the validation methodology, or variations in assembly integrity. The JH-T2T genome assembly enables more precise characterization of SVs. This precision is crucial, as incomplete assemblies or technological limitations can result in incorrect assemblies or omissions of important SVs.

One of the most critical improvements offered by the T2T assembly is its superior ability to capture SVs that affect important genes. This enhanced coverage allows for the accurate identification and characterization of SVs, which are crucial for understanding genetic variation and its influence on phenotypic traits. For example, among the SVs accurately captured by the T2T genome, we identified notable examples such as the largest SV located in the left telomere region of chromosome 14, which includes important genes like *CYP2C42* and *CYP2C18*. In this study, we systematically characterized large SVs between the two pig genome assemblies, identifying 204 large SVs with gene-model differences. Most of these large SVs

overlapped with candidate regions for selection signatures, underscoring the importance of these SVs for pig population differentiation. Some genes likes *LGALS12*, *GPAM*, *CACNB2*,are implicated in essential metabolic pathways and can influence important economically traits [61]. The large SVs also contained genes related to immune response, such as *LY9*, *ITLN2*, and *CHIA*, which may be linked to asthma susceptibility of JH pig [63,64]. The insertion found in the *SLA-DOB* gene, which serves to enhance the immune system's response and is relevant to transplant rejection [72].

Conclusions

In conclusion, the JH-T2T genome assembly represents a major leap forward in pig genomics. Its high quality and near-complete coverage significantly enhance our ability to capture and characterize SVs, particularly those harboring important genes. This improvement not only refines the reference genome but also serves as a powerful tool for genetic studies and breeding strategies aimed at improving livestock traits.

Availability of data and materials

The datasets supporting the results of this article are available at http://alphaindex.zju.edu.cn/ALPHADB/download.html. The genotype datasets generated and/or analyzed during the current study are available at PHARP (http://alphaindex.zju.edu.cn/PHARP/index.php) and at the SRA repository (https://www.ncbi.nlm.nih.gov/sra). See the ' MATERIALS AND METHODS ' section above for their availability. Computer code for data processing is available from the authors upon request. The scripts used to process our datasets have been upload in github (https://github.com/CCyeah/JH-T2T).

List of abbreviations

HiFi: High Fidelity; ONT: Oxford Nanopore Technologies; Hi-C: high-throughput chromosome conformation capture; T2T: telomere-to-telomere; CTAB: cetyltrimethylammonium bromide; SDS: Sodium Dodecyl Sulfate; CCS: Circular Consensus Sequencing; TEs: transposable elements; QV: Quality value; BUSCO: Benchmarking Universal Single-Copy Orthologs; NCBI: National Center for Biotechnology Information; VGP: Vertebrate Genomes Project; KEGG: Kyoto Encyclopedia of Genes and Genomes; GO: Gene Ontology ; TPM: transcripts per million; SV: structure variation; WGS: Whole Genome Sequencing; LCRs: low-coverage regions; LINEs: long interspersed nuclear elements; SINEs: short interspersed nuclear elements; LTR: long terminal repeat; CDS: coding sequences; QTL: quantitative trait locus; IMF: intramuscular fat content; MLD: musculus longissimus dorsi; DELs: deletions; INSs: insertions.

Declarations

The pigs used in this study were raised in standard commercial breeding facilities and adhered to routine animal husbandry practices and welfare guidelines. All procedures were conducted in accordance with local animal welfare regulations. No additional ethical approval was required as data collection involved routine farm management practices without invasive procedures.

Consent for publication

Not applicable.

Competing interests

The authors declare that they have no competing interests.

Funding

This work was supported by the National Key Research and Development Program of China (grant no. 2021YFD1200802, 2022YFF1000500, and 2023YFD1300404), National Natural Science Foundation of China (grant nos. 32372831 and 32172691), Key Research and Development Program of Zhejiang Province (grant nos. 2021C02068-2 and 2021C02068-1), the Young Scientists Fund of the National Natural Science Foundation of China (Grant No.32402713).

Authors' contributions

Z.W. and YC.P. conceived and supervised the study. CY.C. and Z.W. wrote the manuscript. CY.C. performed the majority of the analyses. J.M., JB.S., QQ.X., H.C., ZY.Z., F.W., S.L. and XW.Y. prepared the DNA sampling and experiments. H.G., Z.Z. and QS.W. participated in the discussion of the results. All authors read and approved the final manuscript.

Acknowledgments

We thank all the researchers worldwide that made their sequencing data publicly available.

References

1. Lunney JK, Van Goor A, Walker KE, Hailstock T, Franklin J, Dai C. Importance of the pig as a human biomedical model. *Sci Transl Med*. 2021; doi: 10.1126/scitranslmed.abd5758.

2. Niu D, Ma X, Yuan T, Niu Y, Xu Y, Sun Z, et al.. Porcine genome engineering for xenotransplantation. *Advanced Drug Delivery Reviews*. 2021; doi: 10.1016/j.addr.2020.04.001.

3. Klymiuk N, Seeliger F, Bohlooly-Y M, Blutke A, Rudmann DG, Wolf E. Tailored Pig Models for Preclinical Efficacy and Safety Testing of Targeted Therapies. *Toxicol Pathol*. 2016; doi: 10.1177/0192623315609688.

4. Wells KD, Prather RS. Genome-editing technologies to improve research, reproduction, and production in pigs. *Molecular Reproduction Devel*. 2017; doi: 10.1002/mrd.22812.

5. Warr A, Affara N, Aken B, Beiki H, Bickhart DM, Billis K, et al.. An improved pig reference genome sequence to enable pig genetics and genomics research. *GigaScience*. 2020; doi: 10.1093/gigascience/giaa051.

6. Ma H, Jiang J, He J, Liu H, Han L, Gong Y, et al.. Long-read assembly of the Chinese indigenous Ningxiang pig genome and identification of genetic variations in fat metabolism among different breeds. *Molecular Ecology Resources*. 2022; doi: 10.1111/1755-0998.13550.

7. Zhou R, Li S, Yao W, Xie C, Chen Z, Zeng Z, et al.. The Meishan pig genome reveals structural variation-mediated gene expression and phenotypic divergence underlying Asian pig domestication. *Mol Ecol Resour*. 2021; doi: 10.1111/1755-0998.13396.

8. Jiang Y-F, Wang S, Wang C-L, Xu R-H, Wang W-W, Jiang Y, et al.. Pangenome obtained by long-read sequencing of 11 genomes reveal hidden functional structural variants in pigs. *iScience*. 2023; doi: 10.1016/j.isci.2023.106119.

9. Tian X, Li R, Fu W, Li Y, Wang X, Li M, et al.. Building a sequence map of the pig pan-genome from multiple de novo assemblies and Hi-C data. *Sci China Life Sci*. 2020; doi: 10.1007/s11427-019-9551-7.

10. Aganezov S, Yan SM, Soto DC, Kirsche M, Zarate S, Avdeyev P, et al.. A complete reference genome improves analysis of human genetic variation. *Science*. 2022; doi: 10.1126/science.abl3533.

11. Song J-M, Xie W-Z, Wang S, Guo Y-X, Koo D-H, Kudrna D, et al.. Two gap-free reference genomes and a global view of the centromere architecture in rice. *Molecular Plant*. 2021; doi: 10.1016/j.molp.2021.06.018.

12. Li K, Jiang W, Hui Y, Kong M, Feng L-Y, Gao L-Z, et al.. Gapless indica rice genome reveals synergistic contributions of active transposable elements and segmental duplications to rice genome evolution. *Molecular Plant*. 2021; doi: 10.1016/j.molp.2021.06.017.

13. Kille B, Balaji A, Sedlazeck FJ, Nute M, Treangen TJ. Multiple genome alignment in the telomere-to-telomere assembly era. *Genome Biol*. 2022; doi: 10.1186/s13059-022-02735-6.

14. Ardui S, Ameur A, Vermeesch JR, Hestand MS. Single molecule real-time (SMRT) sequencing comes of age: applications and utilities for medical diagnostics. *Nucleic Acids Research*. 2018; doi: 10.1093/nar/gky066.

15. Cheng H, Concepcion GT, Feng X, Zhang H, Li H. Haplotype-resolved de novo assembly using phased assembly graphs with hifiasm. *Nat Methods*. 2021; doi: 10.1038/s41592-020-01056-5.

16. Jain M, Koren S, Miga KH, Quick J, Rand AC, Sasani TA, et al.. Nanopore sequencing and assembly of a human genome with ultra-long reads. *Nat Biotechnol*. 2018; doi: 10.1038/nbt.4060.

17. Jain M, Olsen HE, Turner DJ, Stoddart D, Bulazel KV, Paten B, et al.. Linear assembly of a human centromere on the Y chromosome. *Nat Biotechnol*. 2018; doi: 10.1038/nbt.4109.

18. Vollger MR, Guitart X, Dishuck PC, Mercuri L, Harvey WT, Gershman A, et al.. Segmental duplications and their variation in a complete human genome. *Science*. 2022; doi: 10.1126/science.abj6965.

19. Rhie A, McCarthy SA, Fedrigo O, Damas J, Formenti G, Koren S, et al.. Towards complete and error-free genome assemblies of all vertebrate species. *Nature*. 2021; doi: 10.1038/s41586-021-03451-0.

20. Wu F, Chen Z, Zhang Z, Wang Z, Zhang Z, Wang Q, et al.. The Role of SOCS3 in Regulating Meat Quality in Jinhua Pigs. *IJMS*. 2023; doi: 10.3390/ijms241310593.

21. Chen H, Rangasamy M, Tan SY, Wang H, Siegfried BD. Evaluation of Five Methods for Total DNA Extraction from Western Corn Rootworm Beetles. Lalueza-Fox C, editor. *PLoS ONE*. 2010; doi: 10.1371/journal.pone.0011963.

22. Marçais G, Kingsford C. A fast, lock-free approach for efficient parallel counting of occurrences of *k* -mers. *Bioinformatics*. 2011; doi: 10.1093/bioinformatics/btr011.

23. Rhie A, McCarthy SA, Fedrigo O, Damas J, Formenti G, Koren S, et al.. Towards complete and error-free genome assemblies of all vertebrate species. *Nature*. 2021; doi: 10.1038/s41586-021-03451-0.

24. Cheng H, Jarvis ED, Fedrigo O, Koepfli K-P, Urban L, Gemmell NJ, et al.. Haplotype-resolved assembly of diploid genomes without parental data. *Nat Biotechnol*. 2022; doi: 10.1038/s41587-022-01261-x.

25. Hu J, Wang Z, Sun Z, Hu B, Ayoola AO, Liang F, et al.. An efficient error correction and accurate assembly tool for noisy long reads. Bioinformatics; 2023 Mar.

26. Zhang X, Zhang S, Zhao Q, Ming R, Tang H. Assembly of allele-aware, chromosomal-scale autopolyploid genomes based on Hi-C data. *Nat Plants*. 2019; doi: 10.1038/s41477-019-0487-8.

27. Durand NC, Robinson JT, Shamim MS, Machol I, Mesirov JP, Lander ES, et al.. Juicebox Provides a Visualization System for Hi-C Contact Maps with Unlimited Zoom. *Cell Systems*. 2016; doi: 10.1016/j.cels.2015.07.012.

28. Hu J, Wang Z, Liang F, Liu S-L, Ye K, Wang D-P. NextPolish2: A Repeat-aware Polishing Tool for Genomes Assembled Using HiFi Long Reads. Zhao F, editor. *Genomics, Proteomics & Bioinformatics*. 2024; doi: 10.1093/gpbjnl/qzad009.

29. Rautiainen M, Nurk S, Walenz BP, Logsdon GA, Porubsky D, Rhie A, et al.. Telomere-to-telomere assembly of diploid chromosomes with Verkko. *Nat Biotechnol*. 2023; doi: 10.1038/s41587-023-01662-6.

30. Wang Z, Zhang Z, Chen Z, Sun J, Cao C, Wu F, et al.. PHARP: a pig haplotype reference panel for genotype imputation. *Sci Rep*. 2022; doi: 10.1038/s41598-022-15851-x.

31. Manni M, Berkeley MR, Seppey M, Simão FA, Zdobnov EM. BUSCO Update: Novel and Streamlined Workflows along with Broader and Deeper Phylogenetic Coverage for Scoring of Eukaryotic, Prokaryotic, and Viral Genomes. Kelley J, editor. *Molecular Biology and Evolution*. 2021; doi: 10.1093/molbev/msab199.

32. Mikheenko A, Prjibelski A, Saveliev V, Antipov D, Gurevich A. Versatile genome assembly evaluation with QUAST-LG. *Bioinformatics*. 2018; doi: 10.1093/bioinformatics/bty266.

33. Rhie A, Walenz BP, Koren S, Phillippy AM. Merqury: reference-free quality, completeness, and phasing assessment for genome assemblies. *Genome Biol*. 2020; doi: 10.1186/s13059-020-02134-9.

34. Li H. Minimap2: pairwise alignment for nucleotide sequences. Birol I, editor. *Bioinformatics*. 2018; doi: 10.1093/bioinformatics/bty191.

35. Brown M, González De la Rosa PM, Mark B. A Telomere Identification Toolkit. Zenodo;

36. Jurka J, Kapitonov VV, Pavlicek A, Klonowski P, Kohany O, Walichiewicz J. Repbase Update, a database of eukaryotic repetitive elements. *Cytogenet Genome Res*. 2005; doi: 10.1159/000084979.

37. Storer J, Hubley R, Rosen J, Wheeler TJ, Smit AF. The Dfam community resource of transposable element families, sequence models, and genome annotations. *Mobile DNA*. 2021; doi: 10.1186/s13100-020-00230-y.

38. Flynn JM, Hubley R, Goubert C, Rosen J, Clark AG, Feschotte C, et al.. RepeatModeler2 for automated genomic discovery of transposable element families. *Proc Natl Acad Sci USA*. 2020; doi: 10.1073/pnas.1921046117.

39. Tempel S. Using and Understanding RepeatMasker. In: Bigot Y, editor. *Mobile Genetic Elements*. Totowa, NJ: Humana Press;

40. Holt C, Yandell M. MAKER2: an annotation pipeline and genome-database management tool for second-generation genome projects. *BMC Bioinformatics*. 2011; doi: 10.1186/1471-2105-12-491.

41. Kim D, Paggi JM, Park C, Bennett C, Salzberg SL. Graph-based genome alignment and genotyping with HISAT2 and HISAT-genotype. *Nat Biotechnol*. 2019; doi: 10.1038/s41587-019-0201-4.

42. Pertea M, Pertea GM, Antonescu CM, Chang T-C, Mendell JT, Salzberg SL. StringTie enables improved reconstruction of a transcriptome from RNA-seq reads. *Nat Biotechnol*. 2015; doi: 10.1038/nbt.3122.

43. Camacho C, Coulouris G, Avagyan V, Ma N, Papadopoulos J, Bealer K, et al.. BLAST+: architecture and applications. *BMC Bioinformatics*. 2009; doi: 10.1186/1471-2105-10-421.

44. Slater G, Birney E. Automated generation of heuristics for biological sequence comparison. *BMC Bioinformatics*. 2005; doi: 10.1186/1471-2105-6-31.

45. Korf I. Gene finding in novel genomes. *BMC Bioinformatics*. 2004; doi: 10.1186/1471-2105-5-59.

46. Stanke M, Keller O, Gunduz I, Hayes A, Waack S, Morgenstern B. AUGUSTUS: ab initio prediction of alternative transcripts. *Nucleic Acids Research*. 2006; doi: 10.1093/nar/gkl200.

47. Altschul S. Gapped BLAST and PSI-BLAST: a new generation of protein database search programs. *Nucleic Acids Research*. 1997; doi: 10.1093/nar/25.17.3389.

48. Bairoch A, Apweiler R. The SWISS-PROT protein sequence data bank and its supplement TrEMBL in 1999. *Nucleic Acids Research*. 1999; doi: 10.1093/nar/27.1.49.

49. Quevillon E, Silventoinen V, Pillai S, Harte N, Mulder N, Apweiler R, et al.. InterProScan: protein domains identifier. *Nucleic Acids Research*. 2005; doi: 10.1093/nar/gki442.

50. Moriya Y, Itoh M, Okuda S, Yoshizawa AC, Kanehisa M. KAAS: an automatic genome annotation and pathway reconstruction server. *Nucleic Acids Research*. 2007; doi: 10.1093/nar/gkm321.

51. Chen S, Zhou Y, Chen Y, Gu J. fastp: an ultra-fast all-in-one FASTQ preprocessor. *Bioinformatics*. 2018; doi: 10.1093/bioinformatics/bty560.

52. Marçais G, Delcher AL, Phillippy AM, Coston R, Salzberg SL, Zimin A. MUMmer4: A fast and versatile genome alignment system. Darling AE, editor. *PLoS Comput Biol*. 2018; doi: 10.1371/journal.pcbi.1005944.

53. Heller D, Vingron M. SVIM-asm: structural variant detection from haploid and diploid genome assemblies. Robinson P, editor. *Bioinformatics*. 2021; doi: 10.1093/bioinformatics/btaa1034.

54. Bu D, Luo H, Huo P, Wang Z, Zhang S, He Z, et al.. KOBAS-i: intelligent prioritization and exploratory visualization of biological functions for gene enrichment analysis. *Nucleic Acids Research*. 2021; doi: 10.1093/nar/gkab447.

55. Shumate A, Salzberg SL. Liftoff: accurate mapping of gene annotations. Valencia A, editor. *Bioinformatics*. 2021; doi: 10.1093/bioinformatics/btaa1016.

56. Vasimuddin Md, Misra S, Li H, Aluru S. Efficient Architecture-Aware Acceleration of BWA-MEM for Multicore Systems. *2019 IEEE International Parallel and Distributed Processing Symposium (IPDPS)*. Rio de Janeiro, Brazil: IEEE;

57. Danecek P, Auton A, Abecasis G, Albers CA, Banks E, DePristo MA, et al.. The variant call format and VCFtools. *Bioinformatics*. 2011; doi: 10.1093/bioinformatics/btr330.

58. Szpiech ZA, Hernandez RD. selscan: An Efficient Multithreaded Program to Perform EHH-Based Scans for Positive Selection. *Molecular Biology and Evolution*. 2014; doi: 10.1093/molbev/msu211.

59. Xu M, Guo L, Gu S, Wang O, Zhang R, Peters BA, et al.. TGS-GapCloser: A fast and accurate gap closer for large genomes with low coverage of error-prone long reads. *GigaScience*. 2020; doi: 10.1093/gigascience/giaa094.

60. Meyne J, Baker RJ, Hobart HH, Hsu TC, Ryder OA, Ward OG, et al.. Distribution of non-telomeric sites of the (TTAGGG)n telomeric sequence in vertebrate chromosomes. *Chromosoma*. 1990; doi: 10.1007/BF01737283.

61. Skinner TM, Anderson JA, Haley CS, Archibald AL. Assessment of *SULT1A1* , *CYP2A6* and *CYP2C18* as candidate genes for elevated backfat skatole levels in commercial and experimental pig populations. *Animal Genetics*. 2006; doi: 10.1111/j.1365-2052.2006.01502.x.

62. Mitka I, Ropka-Molik K, Tyra M. Functional Analysis of Genes Involved in Glycerolipids Biosynthesis (GPAT1 and GPAT2) in Pigs. *Animals*. 2019; doi: 10.3390/ani9060308.

63. Cuenca M, Puñet-Ortiz J, Ruart M, Terhorst C, Engel P. Ly9 (SLAMF3) receptor differentially regulates iNKT cell development and activation in mice. *Eur J Immunol*. 2018; doi: 10.1002/eji.201746925.

64. Louten J, Mattson JD, Malinao M-C, Li Y, Emson C, Vega F, et al.. Biomarkers of Disease and Treatment in Murine and Cynomolgus Models of Chronic Asthma. *Biomark Insights*. 2012; doi: 10.4137/BMI.S9776.

65. Ladowski JM, Hara H, Cooper DKC. The Role of SLAs in Xenotransplantation. *Transplantation*. 2021; doi: 10.1097/TP.0000000000003303.

66. Aganezov S, Yan SM, Soto DC, Kirsche M, Zarate S, Avdeyev P, et al.. A complete reference genome improves analysis of human genetic variation. *Science*. 2022; doi: 10.1126/science.abl3533.

67. Li T-T, Xia T, Wu J-Q, Hong H, Sun Z-L, Wang M, et al.. De novo genome assembly depicts the immune genomic characteristics of cattle. *Nat Commun*. 2023; doi: 10.1038/s41467-023-42161-1.

68. Huang Z, Xu Z, Bai H, Huang Y, Kang N, Ding X, et al.. Evolutionary analysis of a complete chicken genome. *Proc Natl Acad Sci USA*. 2023; doi: 10.1073/pnas.2216641120.

69. You X, Fang Q, Chen C, Cao J, Fu S, Zhang T, et al.. A near complete genome assembly of the East Friesian sheep genome. *Sci Data*. 2024; doi: 10.1038/s41597-024-03581-w.

70. Li M, Tian S, Jin L, Zhou G, Li Y, Zhang Y, et al.. Genomic analyses identify distinct patterns of selection in domesticated pigs and Tibetan wild boars. *Nat Genet*. 2013; doi: 10.1038/ng.2811.

71. Kang M, Ahn B, Youk S, Jeon H, Soundarajan N, Cho E-S, et al.. Individual and population diversity of 20 representative olfactory receptor genes in pigs. *Sci Rep*. 2023; doi: 10.1038/s41598-023-45784-y.

72. Lunney JK, Ho C-S, Wysocki M, Smith DM. Molecular genetics of the swine major histocompatibility complex, the SLA complex. *Developmental & Comparative Immunology*. 2009; doi: 10.1016/j.dci.2008.07.002.

Tables

Table 1. Summary information of JH-T2T, Sscrofa11.1, Ningxiang and MSCAAS v1 assemblies.

**Terms**	**JH-T2T**	**Sscrofa11.1**[5]	**Ningxiang**[6]	**MSCAAS v1**[7]
Contig N50 (Mb)	100.5	48.2	26.1	48.1
Contig number	26	1,117	305	152
Scaffold N50 (Mb)	142.7	88.2	139.0	139.0
Scaffold number	20	20	19	19
Gaps	6	103	286	133
Assembly size (Gb)	2.61	2.50	2.44	2.50
Average length of CDS (bp)	1,593	1,668	1,601	1,379
Protein-coding genes	23,924	20,661	20,914	22,855

Figures and legends

Figure 1. Summary of JH-T2T pig genome assembly. (A) Schematic diagram illustrating the pipeline for genome assembly and annotation. (B) Hi-C chromatin interactions of the assembled JH-T2T genome. (C) Comparison of the contiguity between released assemblies and JH-T2T assembly. (D) Landscape of the assembled JH-T2T genome, showing chromosomes, GC contents, gene, repeat and TE density,

SNPs, and InDels in different tracks from outer to inner. (E) Composition ratio of repeat elements in JH-T2T. (F) Gene annotations of JH-T2T. (G) Genome-wide telomere portrait of JH-T2T. The black boxes indicate chromosomal loci of the tandemly repeated telomeric motif in the primary assembly. The heatmap shows the chromosome-wide gene density in non-overlapping 1 Mb windows.

Figure 2. Sequencing coverage, mapping stats and filling gaps in JH-T2T assembly. (A-C) Comparison of DNA sequencing read mapping rates when whole genome resequencing reads from Asian (Left) and European (Right) pig mapped to MSCAAS v1, Ningxiang (NX), Sscrofa11.1 and JH-T2T genome assemblies, respectively. (D) Comparison of RNA sequencing read mapping rates for Asian (Left) and European (Right) mapped to the Duroc (Sscrofa11.1) and the JH-T2T genome assembly, respectively. (E) Whole-genome sequence coverage of mapped WGS, HiFi and ONT reads. Gaps distribution across chromosomes in JH-T2T. Black indicates filled gaps, while red indicates unfilled gaps. (F-I) Whole-genome sequence coverage of mapped WGS (green), HiFi (blue) and ONT (pink) reads specifically in gap regions (1:153402833−153420736, 2:163627346−163640938, 10:34859081−34861523, 8:57448504-57483687 8:57507305-57519256 8:57568078-57569395 8:57569985-57594383).

Figure 3. Global comparison of Sscrofa11.1 and JH-T2T genomes. (A) Collinearity between the JH-T2T and Sscrofa11.1 genomes. Collinear regions are shown by gray lines. Black triangles indicate the presence of telomere sequence repeats. (B) Density distribution of SVs across the JH-T2T genome. (C) Proportions

of SVs in 5'UTR, 3'UTR, CDS, introns, and intergenic regions. (D) Percentage of validated SVs categorized by length.

Supplementary Tables

Supplementary Table 1. Summary of the sequence data used for JH-T2T assembly.

Supplementary Table 2. Statistics of draft assembly.

Supplementary Table 3. Statistics on the number and length of clusters of individual chromosomes and Genomic mount rate.

Supplementary Table 4. Genome statistics, predicted telomeres and centromeres.

Supplementary Table 5. Scaffold and contig length of four assemblies.

Supplementary Table 6. BUSCOs analysis of JH-T2T, Sscrofa11.1, MS, NX.

Supplementary Table 7. Information of the 939 WGS pigs.

Supplementary Table 8. Information of the 111 RNA pigs.

Supplementary Table 9. Gap positions of JH-T2T.

Supplementary Table 10. Summary of repeat content of JH-T2T.

Supplementary Table 11. Lost and gain genes between JH-T2T and Sscrofa11.1.

Supplementary Table 12. Lost and gain genes enriched KEGG PATHWAY and Gene Ontology.

Supplementary Table 13. List of SVs between JH-T2T and Sscrofa11.1.

Supplementary Table 14. Statistics of SV-related QTL.

Supplementary Table 15. SV with WGS validation.

Supplementary Table 16. Large SVs localized on Sscrofa11.1 reference and overlapping gene ID on Large SVs.

Supplementary Table 17. Selected large SVs localized on Sscrofa11.1 reference and overlapping gene ID on Large SVs.

Supplementary Table 18. Large SV genes enriched KEGG PATHWAY and Gene Ontology.

Supplementary Figures

Figure S1. Overview of the data processing pipeline used for the assembly and genomic analysis of JH-T2T genome.

Figure S2. Summary of data used for JH-T2T assembly.

Figure S3. Misassembly correction using Hi-C data.

Figure S4: Haplotype assemblies of paternal and maternal.

Figure S5. Evaluation of the JH-T2T assembly.

Figure S6. Replaced gap regions.

Figure S7. Coverage of WGS, HiFi, and ONT read on 47 filled gaps region of the JH-T2T assembly.

Figure S8. Predicted centromeres' locations in the JH-T2T assembly.

Figure S9. SVs between the Sscrofa11.1 and JH-T2T.

Figure S10. Selective regions on Large SVs between the Sscrofa11.1 and the JH-T2T genome assembly.

Figure S11. Selective regions on Large SVs between the Sscrofa11.1 and JH-T2T genome assemblies.

Table Click here to access/download;Table;table.xls

Table 1. Summary information of JH-T2T, Sscrofa11.1, Ningxiang and MSCAAS

Terms	JH-T2T	Sscrofa11.1[5]	Ningxiang[6]	MSCAAS v1[7]
Contig N50 (Mb)	100.5	48.2	26.1	48.1
Contig number	26	1,117	305	152
Scaffold N50 (Mb)	142.7	88.2	139	139
Scaffold number	20	20	19	19
Gaps	6	103	286	133
Assembly size (Gb)	2.61	2.5	2.44	2.5
Average length of CDS (bp)	1,593	1,668	1,601	1,379
Protein-coding genes	23,924	20,661	20,914	22,855

Click here to access/download;Figure;fig3.pdf

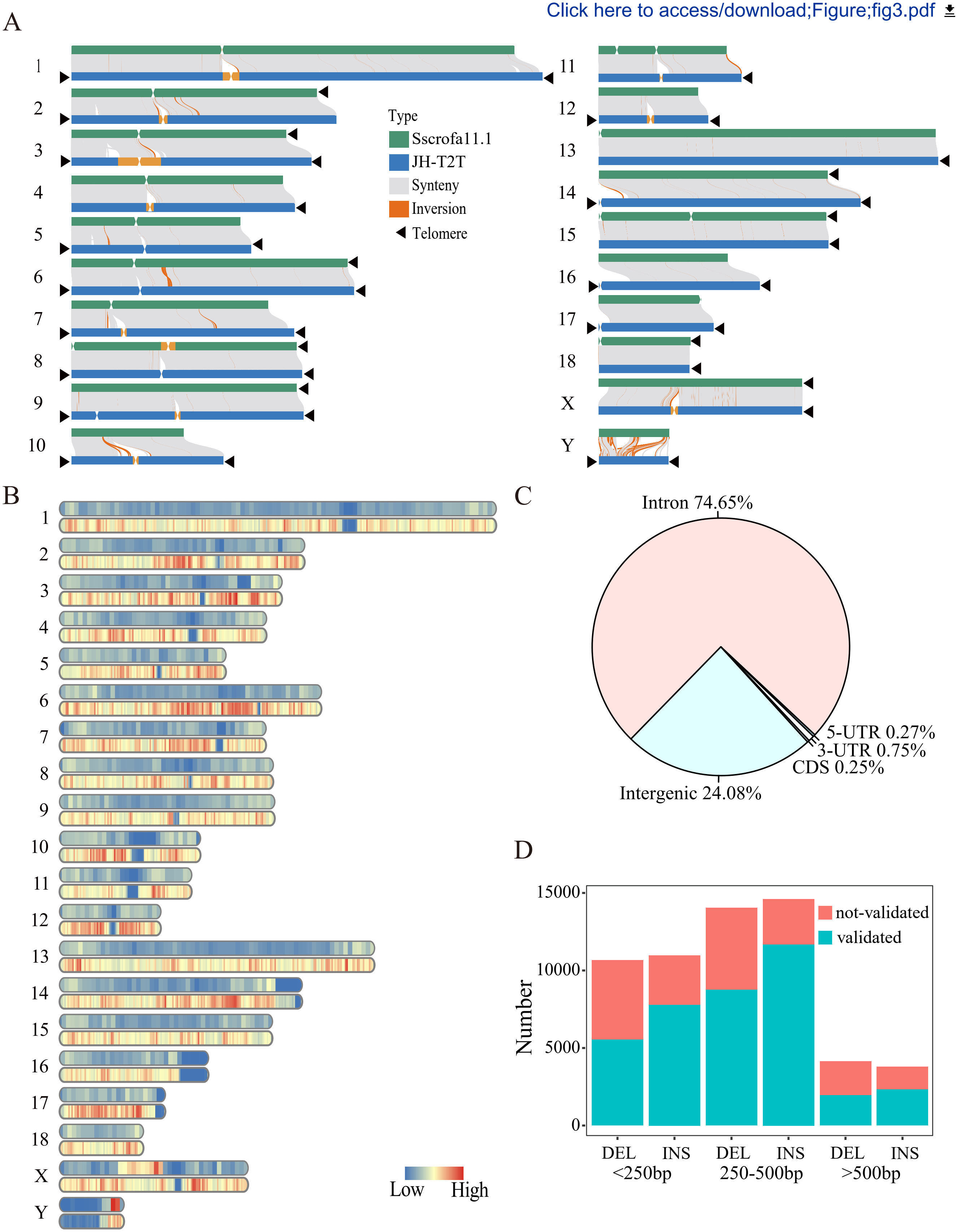

Click here to access/download;Figure;fig2.pdf

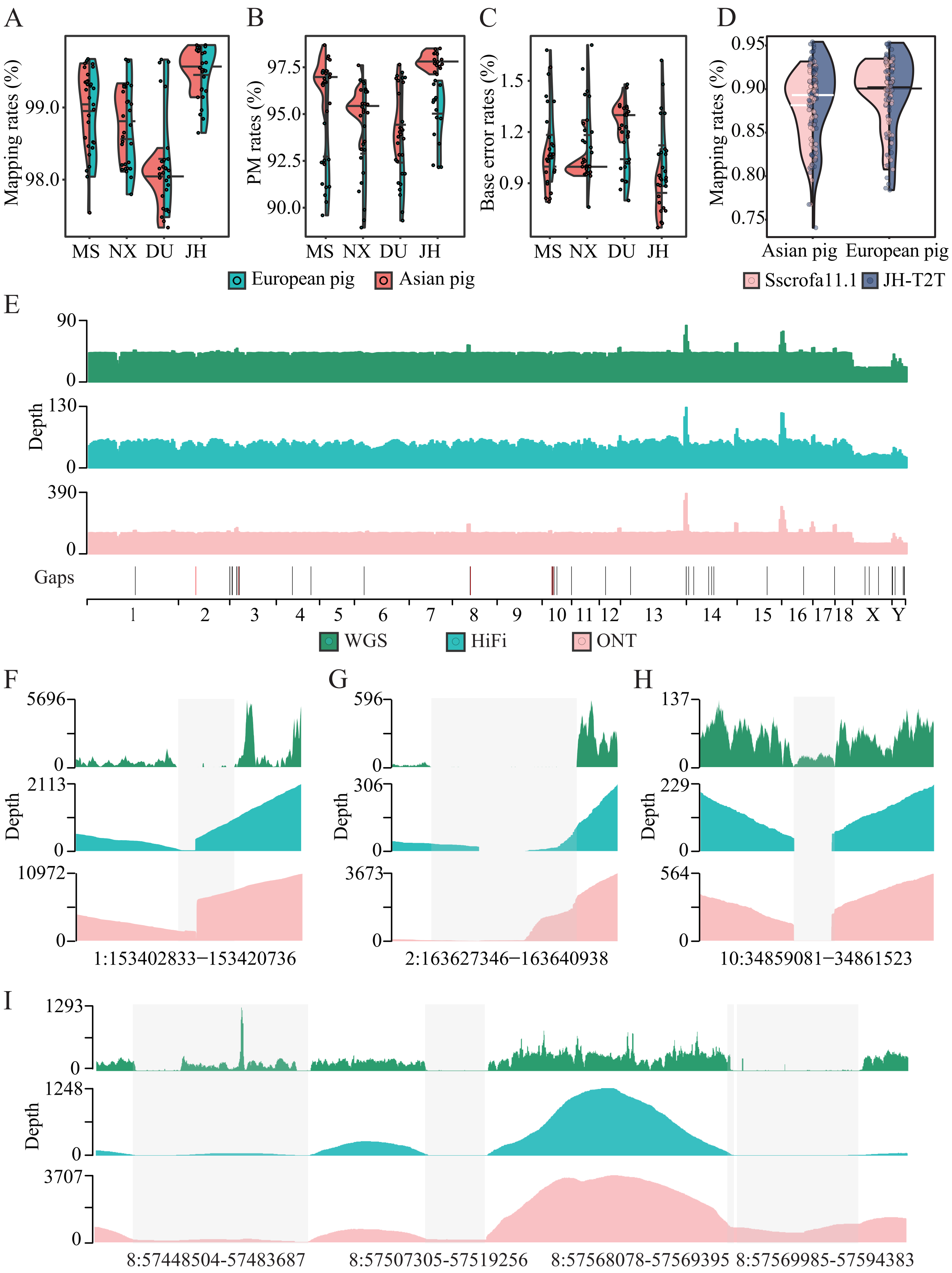

Click here to access/download;Figure;fig1.pdf

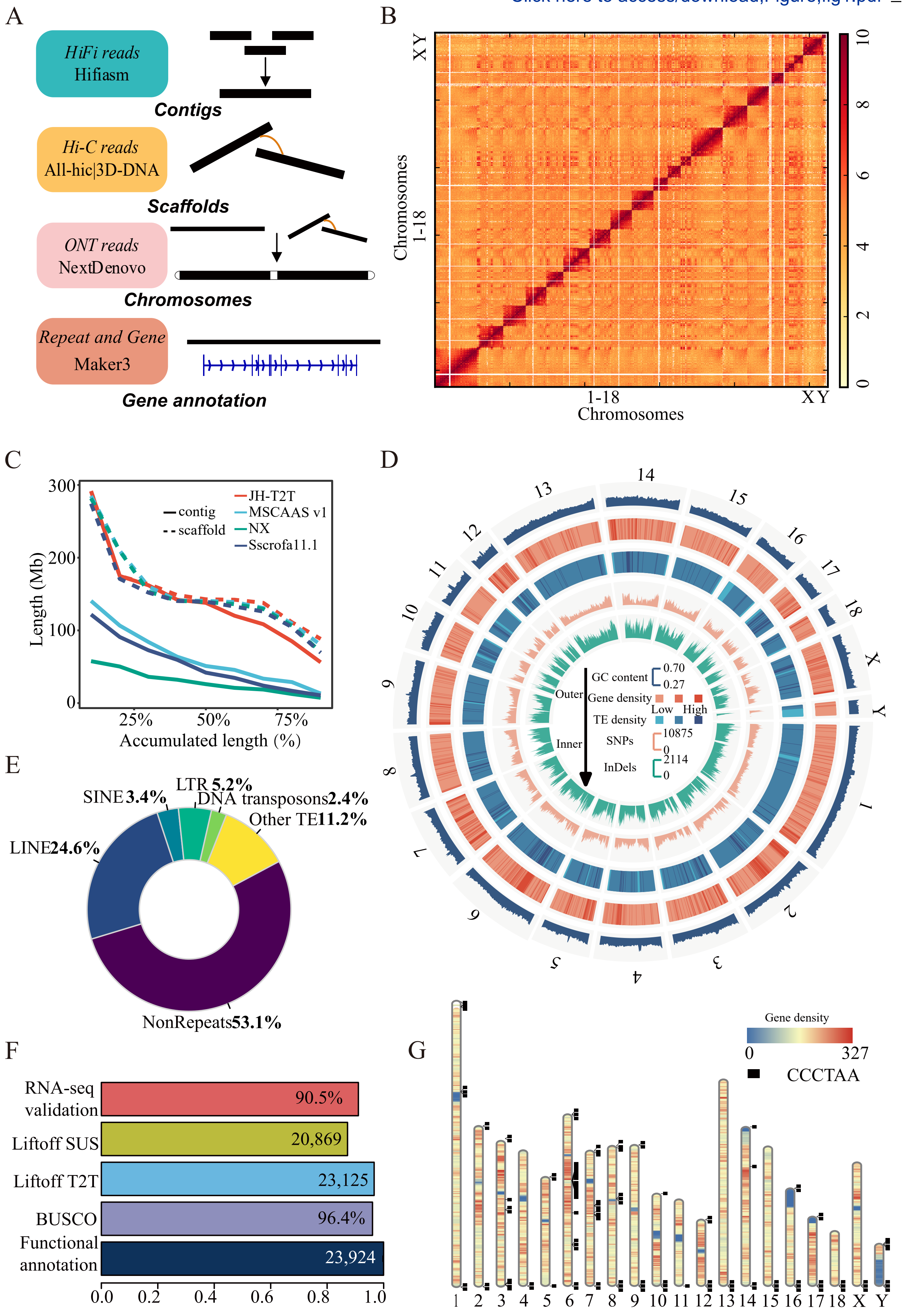

Click here to access/download
Supplementary Material
Supplementary Materials-v2.docx

Click here to access/download
Supplementary Material
Supplementary Tables-v2.xlsx
