# Supplementary material for: A near telomere-to-telomere genome assembly of the Jinhua pig: enabling more accurate genetic research
Source: Gigascience. 2025 May 15;14:giaf048. doi: 10.1093/gigascience/giaf048 (PMC12080228; doi:10.1093/gigascience/giaf048)
Supplement: giaf048_Authors_Response_To_Reviewer_Comments_Original_Submission [file giaf048_authors_response_to_reviewer_comments_original_submission.pdf]

Author's Response To Reviewer Comments

RESPONSE LETTER TO THE REVIEWERS
Response to Editor's Comments

Thank you for your email and for providing the reviewers' comments on our manuscript, "The first near-complete genome assembly of pig: enabling more accurate genetic research" (GIGA-D-24-00462). We appreciate the time and effort the reviewers have taken to evaluate our work and provide constructive feedback. We are pleased to learn that the manuscript is of interest to GigaScience and are committed to addressing the reviewers' concerns to improve the quality and impact of our study.
We have carefully considered all the comments and will address them in a revised version of the manuscript. Below, we provide a brief outline of our revisions:

Haplotype-resolved assemblies (Reviewers 1):
We agree with the reviewer that haplotype-resolved assemblies would add significant value to the manuscript. In our revision, we included more results of employing a trio-binning approach to generate haplotype resolved assemblies from the two sequenced parental genomes.

Methodological accuracy and result interpretation (All Reviewers):
In response to the other points raised by the reviewers, we are committed to providing a more accurate description of our methods and a careful interpretation of our results in our revision. We believe that addressing these concerns will significantly enhance the quality of our manuscript.

Adaptation to the "Data Note" format:
We acknowledge the editor's suggestion to consider the manuscript for the Data Note section. We have revised the manuscript, ensuring that the structure and content align with the journal's formatting guidelines for this article type.

We have submitted a point-by-point response to the reviewers' comments along with the revised manuscript, detailing all changes made and providing a rebuttal for any points where we may have a differing perspective. We will also ensure that all data (http://alphaindex.zju.edu.cn/ALPHADB/download.html) and code (https://github.com/CCyeah/JH-T2T) are updated and publicly accessible as required.

Thank you again for the opportunity to improve our work. Please do not hesitate to contact us if you have any further questions or require additional information.

Response to Reviewer 1's Comments

Reviewer #1: The manuscript describes the T2T genome assembly for the Chinese pig breed Jinhua, which presents a vast improvement compared to the current reference genome of the Duroc pig TJTabasco (build11.1). The results and methodology use for the assembly are described clearly and the authors show the improvement of this assembly by a detailed comparison with the current reference 11.1. While clearly of interest to be published, several aspects of the manuscript should be improved. Most of these changes are minor modifications or inaccuracies in the presentation of the results. However, there are two major aspects that need further attention.

Response: Thank you for your valuable comments and insightful suggestions on our manuscript. We greatly appreciate your insights, particularly regarding the T2T genome assembly of the Jinhua pig and the identification of selective sweep regions. We have carefully revised the manuscript in line with your recommendations. Below, you will find point-by-point responses addressing the specific modifications made. Thank you for your detailed review and constructive feedback

1. The T2T assembly presented, represents a combination of the two haplotypes of the pig sequenced. I am surprised why the authors did not also develop two haplotype resolved assemblies of this genome.

Haplotype resolved assemblies will be the assemblies of choice for future developments of a reference pan-genome for pigs. The authors describe that they have sequenced the two parents of the sequenced F1 individual, so why did they not use the trio-binning approach to also develop haplotype resolved assemblies. I, think adding these to the manuscript would be a vast improvement for this important resource.
Response: We appreciate your suggestion regarding the development of haplotype resolved assemblies. We agree that these assemblies would be invaluable for establishing a more comprehensive reference pan-genome for pigs. In our revision, we included more results of employing a trio-binning approach to generate haplotype resolved assemblies from the two sequenced parental genomes. The initial assembly for the autosomes of the haplotype-resolved genomes was performed by using hifiasm(v0.16.1)[1] and verkko(v1.1)[2] based on the trio mode with HiFi reads, ultra-long ONT reads and the parents' short reads. The more continuous contigs were selected from the two assemblies to represent. This results in a maternal assembly with 157 gaps and paternal assembly with 99 gaps (Supplementary Table S2). Subsequently, gap closing was performed using TGS-Gapcloser [3] with the verkko assembly, resulting 116 and 42 gaps, respectively. Since our HiFi reads and ultra-long ONT reads are limited, the final diploid JH reference genome has NG50 of 147.60 Mb and 143.06 Mb for maternal and paternal genomes, respectively. And we further evaluated JH.mat and JH.pat with quality values (QV) and coverage distribution of ONT and PacBio HiFi reads. By comparing the linear genomes of two complete haplotypes, we detected ~7.23 million single nucleotide variants (SNVs), 1,165,610 small insertions or deletions (indels) (< 50 bp), and 26,701 SVs (≥ 50 bp).We believe this addition will significantly enhance the value of our manuscript and provide a critical resource for future research in swine genomics.

1. Cheng H, Concepcion GT, Feng X, Zhang H, Li H. Haplotype-resolved de novo assembly using phased assembly graphs with hifiasm. Nat Methods. 2021; doi: 10.1038/s41592-020-01056-5.
2. Rautiainen M, Nurk S, Walenz BP, Logsdon GA, Porubsky D, Rhie A, et al.. Telomere-to-telomere assembly of diploid chromosomes with Verkko. Nat Biotechnol. 2023; doi: 10.1038/s41587-023-01662-6.
3. Xu M, Guo L, Gu S, Wang O, Zhang R, Peters BA, et al.. TGS-GapCloser: A fast and accurate gap closer for large genomes with low coverage of error-prone long reads. GigaScience. 2020; doi: 10.1093/gigascience/giaa094.

2. The results described for the identification of selective sweep regions is not very convincing. This analysis shows differences in the genomes of two breeds: Duroc and Jinhua. However, these breeds have a very different origin of domestication of wild boars that diverged 1 million years ago, followed by the development of a wide range of different breeds selected for different traits. Therefore, the comparison made by the authors cannot distinguish between differences in evolution of Chinese and European Wild Boar, more recent selection after breed formation and even drift. To be able to do so, these analyses would need the inclusion of additional breeds and wild boars from China and Europe. Alternatively, the authors can decide to tone down this part of the manuscript or even delete it altogether, as it does not add to the major message of the manuscript.

Response: Thank you for your insightful comments regarding the analysis of selective sweep regions. We acknowledge that the significant evolutionary divergence between the Duroc and Jinhua breeds, along with their distinct domestication histories, complicates the interpretation of our results. Alternatively, we will revise the manuscript to tone down the discussion on selective sweeps, ensuring it aligns more closely with the main message of our study. Your feedback is invaluable for enhancing the clarity and impact of our work.

Minor comments
Line 34: Change the sentence to: "with thousands of segments and centromeres and telomeres missing"
Response: Thank you for your suggestion. We have revised the sentence as suggested.

Line 37: Insert "and Hi-C" after "long reads"
Response: Thank you for your reminder. The phrase "and Hi-C" has been added as recommended.

Line 46: Delete " such as GPAM, CYP2C18, LY9, ITLN2, and CHIA"
Response: Thank you for your suggestion. We have removed the specific gene names from this sentence.

Line 54: Insert "potential" before "xenotransplantation"
Response: Thank you for your suggestion. The word "potential" has been added before "xenotransplantation".

Line 82: Delete "in response to the gap of a T2T-level pig genome" as this does not add anything and the use of "gap" in this context is confusing.
Response: Thank you for your suggestion. We have deleted the phrase "in response to the gap of a T2T-level pig genome" to improve clarity.

Line 93: Change "The fresh blood" to "Fresh blood"
Response: Thank you for your suggestion. The text has been changed to "Fresh blood" as suggested.

Line 100: The authors need to provide a reference for the SDS method
Response: We have included a reference for the SDS method in the revised manuscript, which provides insights into library preparation methods. And the SDS method for Ultra-long DNA was extracted was performed at Wuhan Benagen Technology Co., Ltd for your information. If you need more specific information or additional references, please let me know.
1. Chen H, Rangasamy M, Tan SY, Wang H, Siegfried BD. Evaluation of Five Methods for Total DNA Extraction from Western Corn Rootworm Beetles. Lalueza-Fox C, editor. PLoS ONE. 2010; doi: 10.1371/journal.pone.0011963.

Lines 152-153, line 444, and table S6: This is confusing. The authors mention Genotypes from 939 individuals, but in the table it is shown that they have used WGS data. You need to describe how the WGS data was used to call the genotypes for these individuals. Furthermore, in line 444 you mention 289 JH pigs and 616 DU pigs which together is 905. What about the other 34 individuals shown in table S6?
Response: Thank you for your observations regarding Lines 152-153, line 444, and Table S6. We have revised the relevant tables and content to ensure consistency. There is a total of 925 individuals included. The genotype dataset for 905 individual pigs were extracted from the PHARP database as SNP annotations available on the download site. The remaining 20 individuals and 5 JH pigs and 5 DU pigs were used in the form of raw data rather than genotype data. The methods for utilizing this raw data are described in the "Genome Assembly Quality Assessment" section. Thank you for your question, which will help improve the clarity of our study.

Line 244: Replace "were" by "was" and delete "the" before "fastp"
Response: Thank you for your suggestion. We have changed "were" to "was" and removed "the" before "fastp". Your feedback is greatly appreciated and will help improve the clarity of our work.

Lines 287292: Here you use several times "length of xx Gb and yy contigs". This is not correct as the value for the contigs refers to a number and not a length. Rephase e.g. like "length of xx Gb and consisting of yy contigs"
Response: Thank you for your suggestion. We have rephrased this section (a total length of 2.72 Gb and consisting 187 contigs, a total length of 2.28 Gb and consisting 93 contigs) to accurately reflect the meaning of contig lengths and numbers. Your feedback is greatly appreciated and will help improve the clarity of our work.

Line 294: The use of "bone" sems strange. Either use "backbone" or "core"
Response: Thank you for your suggestion. This have been amended to read "selected it for the backbone of the genome assembly," clarifying the intended meaning. Your feedback is greatly appreciated and will help improve the clarity of our work.

Line 306: Replace "chromosome" by "genome"
Response: Thank you for your suggestion. We have replaced "chromosome" with "genome". Your feedback is greatly appreciated and will help improve the clarity of our work.

Lines 308-309: For the comment "Second, 16 of the 20 chromosomes were each represented by a single contig" you refer to figure 1D however from this figure it cannot be seen if the different chromosomes consist of a single or multiple contigs.

Response: Thank you for your comment regarding the representation of the chromosomes in our manuscript. We initially intended to illustrate the individual chromosomes; however, we mistakenly referred to Figure 1D in this context. We have decided to remove this reference to avoid any confusion, as the figure does not clearly indicate whether the chromosomes are represented by single or multiple contigs. We will ensure that the revised manuscript accurately reflects our findings and provides clearer visual representations where needed. Thank you for bringing this to our attention.

Line 346: Do you mean build 11.1 with "historical genome version". If so, please use that instead.
Response: Thank you for your observation regarding Line 346. We recognize that the term could lead to misunderstandings regarding draft assemblies. To avoid any confusion, we will remove this phrase from the manuscript. Your feedback is greatly appreciated and will help improve the clarity of our work.

Line 349: "post-gap filled"
Response: Thank you for your suggestion. We have corrected this to "post-gap filled". Your feedback is greatly appreciated and will help improve the clarity of our work.

Line 353: The largest gap is 35 kb not 36 kb.
Response: Thank you for your observation regarding Line 353. The largest gap has been accurately reported as 35 kb. Your feedback is greatly appreciated and will help improve the clarity of our work.

Figures 2F-I should be better explained in the legends and the main text (lines 353-358).
Response: We appreciate your feedback regarding the clarity of Figures 2F-I. To enhance the understanding of these figures, we have revised the legends to provide more detailed descriptions of the data presented. Additionally, we have incorporated further explanations in the legends and main text to contextualize the findings and clarify their significance. We believe these changes will improve the overall comprehension of the figures. Thank you for your valuable suggestions.

Lines 378: For the 23,924 genes you refer to supp table S13. However, that table shows a list of SV enriched QTL not these genes. Furthermore, I checked all tables but a table with all the protein coding genes is missing.
Response: Thank you for pointing out the discrepancy regarding Line 378. We mistakenly referenced Supplementary Table S13, which actually contains a list of SV-enriched QTL rather than the 23,924 genes. We will correct this reference in the revised manuscript. Additionally, we plan to publicly share the annotation file that includes detailed information about all the protein-coding genes. Your feedback is greatly appreciated and will help us ensure the accuracy of our manuscript. And we have proceeded to upload the necessary files directly to our public server using the provided credentials so that reviewer can see it when they need (https://alphaindex.zju.edu.cn/ALPHADB/download.html).

Line 380: For the 799 newly anchored genes, refer to table S10. Now you refer to table S17 which shows genes enriched KEGG pathways.
Response: Thank you for your comment regarding Line 380. We apologize for the incorrect reference to Table S17. We meant to refer to Table S11 for the 799 newly anchored genes. We have corrected this reference to table S11 in the revised manuscript to ensure clarity and accuracy. Your feedback is invaluable in helping us refine our work.

Lines 383-386: For the higher gene density in GC rich regions, you refer to figure 1D, but it is impossible to see this correlation from figure 1D. For the density of genes and telomeres, you refer to figure 1G. However, that figure does not show gene densities only repeat densities.
Response: Thank you for your feedback regarding lines 383-386. I appreciate your observations:
λ We have added a picture (Figure S2) to support it. The green bars show the percentage of each partition and the red line shows the number of genes in the 1000kb partition. We can see that the density of genes in GC-rich regions is higher in 1000k bins, especially when the GC content is below 50%.

λ Thank you for your observation regarding figure 1G. The figure legend incorrectly refers to repeat densities when it actually displays gene densities, as mentioned in the main text. We will revise the figure legend to accurately represent the content of the figure. Your feedback is appreciated and will help improve the clarity of our manuscript.

Thank you for bringing these issues to my attention. Your insights will help improve the clarity and accuracy of the manuscript.

Line 406-407. This should be table S11.
Response: Thank you for your observation regarding Lines 406-407. We have made the necessary correction and now correctly reference Table S11 in the revised manuscript. We appreciate your attention to detail, which helps improve the accuracy of our work.

Lines 409-412: For this result you refer to table S11. However, that table only shows data for the gained genes, not the lost genes.
Response: Thank you for highlighting this issue regarding our reference to Table S11. We acknowledge that the table currently only presents data for the gained genes and does not include information on the lost genes. We have referred to the New Table S11 to clarify this and ensure that we provide a comprehensive table that includes both gained and lost genes. Your feedback is greatly appreciated and will help enhance the completeness of our results.

Lines 419-420: You refer to table S12 and figure 3B, but the information is only shown in figure 3B and not in table S12.
Response: Thank you for pointing out the discrepancy regarding our reference to Table S12 and Figure 3B. We will correct the reference to ensure it accurately cites Figure 3B, and we will delete Table S12, as it does not provide relevant information. Your feedback is invaluable in helping us improve the clarity and accuracy of our manuscript.

Line 420: Replace "were" by "is"
Response: Thank you for your suggestion regarding Line 420. We have revised the text to replace "were" with "is" to ensure clarity and accuracy. We appreciate your attention to detail.

Line 422: Better to use "repeats" instead of "they"
Response: Thank you for your suggestion regarding Line 422. We have updated the text to replace "they" with "repeats" for improved clarity. Your feedback is appreciated.

Line 425: "Moreover, 12,129 genes located in these SVs". Unclear to what "these" refers to and I assume that you mean genes that (partially) overlap with SVs? Also, this is an incomplete sentence (verb missing). Likewise, this number is not very meaningful as many of these SVs are within introns. It is much more informative to mention for how many genes SVs affect the CDS.
Response: Thank you for your valuable feedback regarding Line 425. We recognize that the reference to "these" is vague and will clarify that it refers to genes that (partially) overlap with the SVs. We will also revise the sentence to include a verb for completeness.

Line 433 and table S14: This validation is not clear at all. What exactly are these numbers that are shown? You also mention "greater than 1.00" but the table does not contain any number that is greater than 1.00.
Response: Thank you for your comment regarding Line 433 and Table S14. We will clarify the validation process by specifying that we used JH-T2T and Sscrofa11.1 as references, applying a validation criterion that required SV mapping sequence coverage to be 1.00 in one sample and less than 0.90 in another sample. We will ensure that this explanation is clearly presented in the revised manuscript to eliminate any confusion regarding the numbers shown in the table. Your feedback is greatly appreciated.

Line 435: "Table" not "Tables"
Response: Thank you for your observation regarding Line 435. We have corrected "Tables" to "Table" in the revised manuscript. Your attention to detail is appreciated.

Line 436: Change to " SVs with a length larger than 500 bp"
Response: Thank you for your suggestion regarding Line 436. We have made the change to "SVs with a length larger than 500 bp". Your feedback is appreciated.

The term "invalidate" in figure 3D is rather awkward. Better to use "not-validated" and "validated" in this figure.

Response: Thank you for your suggestion regarding the terminology in Figure 3D. We have revised the terms to "not-validated" and "validated" to enhance clarity. Your feedback is appreciated.

Line 449: This should be Table S16.
Response: Thank you for your comment regarding Line 449. We have corrected the reference. Your attention to detail is appreciated.

Line 452: There is not Table S18
Response: Thank you for your observation regarding Line 452. We have corrected the reference.Your feedback is appreciated.

Lines 484-486: Change to "Similarly, in human, the use of the T2T-CHM13 genome assembly yields a more comprehensive view of SVs genome-wide, with a greatly improved balance of insertions and deletions [61]."
Response: Thank you for your suggestion regarding Lines 484-486. We have rephrased the section to read: "Similarly, in human, the use of the T2T-CHM13 genome assembly yields a more comprehensive view of SVs genome-wide, with a greatly improved balance of insertions and deletions [66]." Your feedback has helped enhance the clarity and coherence of our manuscript.

Lines 500-501: Change to "For example, in human, the T2T-CHM13 assembly was shown to improve the analysis of global"
Response: We have updated Lines 500-501 to read: "For example, in humans, the T2T-CHM13 assembly was shown to improve the analysis of global genetic diversity based on 3,202 short read-length samples from the 1KGP dataset." Thank you for your suggestion, which has improved the clarity of this section.

Lines 517-528: This paragraph should be deleted as these genes have already been annotated and described in previous genome builds including 11.1. Why discuss these genes here? Following that line of thinking, almost every gene of the 20,000 can be discussed.
Response: Thank you for your feedback. We appreciate your perspective on the discussion of previously annotated genes. Our intention was to highlight specific genes that may have new insights or relevance in the context of our current findings. However, we understand the importance of maintaining focus and will revise the manuscript to minimize redundancy and ensure that we emphasize novel contributions. Thank you for helping us improve the clarity and relevance of our work.

Line 532: "%" instead of "%%" and insert "which" after "SVs"
Response: Thank you for your valuable feedback. We will make the necessary changes to replace "%%" with "%" and insert "which" after "SVs" in the relevant sections of the manuscript. Your suggestions help enhance the clarity of our work, and we appreciate your attention to detail.

Lines 537-542: These sentences should be deleted. It is common knowledge that second generation sequencing is not very sensitive to identify SVs. The authors also do not provide any results about dPCR.
Response: Thank you for your insightful comments. We acknowledge that the limitations of second-generation sequencing in detecting structural variants (SVs) are widely understood. We will remove the specified sentences to streamline the discussion. We apologize for the oversight regarding the lack of dPCR results. These sentences will be deleted to clarify this point and ensure that it accurately reflects the scope of our study. Your input helps us improve the clarity and integrity of our work. Thank you for your understanding.

Line 544: "affect" rather than "harbor"
Response: We will revise the text to replace "harbor" with "affect" in the relevant section. Your feedback helps enhance the clarity and accuracy of our manuscript, and we appreciate your attention to detail.

Lines 544-547: This is repetitive and has been stated multiple times so better to delete.
Response: Thank you for your comment. We appreciate your observation regarding the repetition in the text. We will review the relevant sections and remove any redundant statements to improve the clarity and conciseness of the manuscript. Your feedback is invaluable in refining our work.

Line 561: "which is serve to immune system's response and relevant to transplant rejection" This is an incorrect sentence and should rephrased.
Response: Thank you for pointing out the error. A possible rephrasing could be: "which serves to enhance the immune system's response and is relevant to transplant rejection." We appreciate your attention to detail and will ensure this correction is made in the manuscript.

Lines 562-568: I don't agree with is statement and suggest to remove it from the discussion.
Response: Thank you for your feedback. We respect your perspective and will remove the statement from the discussion. Your insights are valuable in ensuring the clarity and focus of our manuscript.

Reviewer #2: The first near-complete genome assembly of pig: enabling more accurate genetic research

The authors describe the telomere-to-telomere assembly of a Jinhua breed pig. They sequenced genomic DNA from whole blood with PacBio HiFi and Oxford Nanopore (ONT) long-read technologies as well as Illumina for short reads. They generated HiC data for scaffolding from blood and extracted RNA from 19 tissues for short read RNAseq for gene annotation. A hifiasm assembly was generated with the HiFi data and scaffolded with HiC to chromosome level with 63 gaps. The scaffolded assembly was gap filled with contigs from a NextDenovo assembly of the ONT data bringing the gaps down to 14. Finally, the assembly was manually curated with juicebox somehow closing a further 8 gaps. This needs to be clarified. Standard assembly assessments were performed as well as genome annotation. The authors compared their assembly to the current reference, Sscrofa11.1, and called SVs between the assemblies. The SVs were validated with additional Jinhua and Duroc animals. They then identifiedsignatures of selection present in some of the largest SVs.

General comments:
The manuscript is mostly easy to read but would benefit from further editing for language throughout. The described assembly appears to be high quality and quite contiguous. Although the authors do mention obtaining parental samples and claim the assembly is fully phased, there is no mention of how this was done. There are many additional places where the methods could be described more fully including the addition of parameters used.
Response: We are glad to hear that you found the manuscript mostly easy to read and that the assembly appears to be of high quality. Thank you for your valuable comments and insightful suggestions.
λ We will ensure to perform a thorough language edit throughout the manuscript to enhance clarity. Regarding the phasing of the assembly, we will provide a clear explanation of the methodology used to achieve the fully phased assembly, including the specific techniques and strategies employed. And we have made sure the bioinformatics pipeline scripts uploaded to our GitHub repository at https://github.com/CCyeah/JH-T2T. Thank you for your detailed assessment of our manuscript. We appreciate your summary of our methods and findings.
λ We have rephrased the process of manual curation with Juicebox and how it contributed to closing the remaining gaps in our assembly. Additionally, we will ensure that all components of the assembly and validation processes are clearly described to enhance the reader's understanding. And we have made our code available through GitHub (https://github.com/CCyeah/JH-T2T). This repository includes detailed descriptions of the assembly process and validation methods, providing additional context for our research. Your feedback is crucial in improving the clarity and rigor of our work, and we are grateful for your insights.
λ In our revision, we also included more results of employing a trio-binning approach to generate haplotype resolved assemblies from the two sequenced parental genomes. The initial assembly for the autosomes of the haplotype-resolved genomes was performed by using hifiasm(v0.16.1) [1] and verkko(v1.1) [2] based on the trio mode with HiFi reads, ultra-long ONT reads and and the parents’ short reads. The more continuous contigs were selected from the two assemblies to represent. This results in a maternal assembly with 157 gaps and paternal assembly with 99 gaps (Supplementary Table S2). Subsequently, gap closing was performed using TGS-Gapcloser [3] with the verkko assembly, resulting 116 and 42 gaps, respectively. Since our HiFi reads and ultra-long ONT reads are limited, the final diploid JH reference genome has NG50 of 147.60 Mb and 143.06 Mb for maternal and paternal genomes, respectively. And we further evaluated JH.mat and JH.pat with quality values (QV) and coverage distribution of ONT and PacBio HiFi reads. By comparing the linear genomes of two complete haplotypes, we detected ~7.23 million single

nucleotide variants (SNVs), 1,165,610 small insertions or deletions (indels) (< 50 bp), and 26,701 SVs (≥ 50 bp).We believe this addition will significantly enhance the value of our manuscript and provide a critical resource for future research in swine genomics.
1. Cheng H, Concepcion GT, Feng X, Zhang H, Li H. Haplotype-resolved de novo assembly using phased assembly graphs with hifiasm. Nat Methods. 2021; doi: 10.1038/s41592-020-01056-5.
2. Rautiainen M, Nurk S, Walenz BP, Logsdon GA, Porubsky D, Rhie A, et al.. Telomere-to-telomere assembly of diploid chromosomes with Verkko. Nat Biotechnol. 2023; doi: 10.1038/s41587-023-01662-6.
3. Xu M, Guo L, Gu S, Wang O, Zhang R, Peters BA, et al.. TGS-GapCloser: A fast and accurate gap closer for large genomes with low coverage of error-prone long reads. GigaScience. 2020; doi: 10.1093/gigascience/giaa094.

Specific comments:
Line 39 - Figure 1 only displays 34 telomeres, not 35. Additionally, I was only able to detect 33 telomeres using seqtk telo. Seqtk only reports telomeres at the beginning and end of sequences, digging further, the telomere on chr2 is ~59kb from the end of the chromosome, perhaps indicating a misassembly.
Response: Thank you for your careful review and observations regarding Figure 1. We utilized the vertebrate telomeric repeat (6-mer TTAGGG/CCCTAA) to identify telomeres using the Tidk (v0.2.0) tool. This software detected telomeric repeat sequences throughout all the sequences, as displayed in Figure 1. In response to your suggestion, we have re-identified the telomeres using seqtk telo. We will correct the telomere counts to accurately reflect the presence of 33 telomeres in Table S4 and the relevant sections, particularly regarding the telomere on chromosome 2. Thank you for your valuable guidance on this matter.

Lines 79-81 - there are not hundreds of species with gap free genome assemblies and reference 19 does not claim that there are.
Response: We appreciate your insight and will modify the statement to accurately represent that "the application of third-generation sequencing and assembly technologies to high-fidelity long reads will be contributed to the creation of gap-free genome assemblies across hundreds of species".

Line 82 - the assembly is not gap-free, replace with "nearly gap-free"
Response: We have revised the text to describe the assembly as "nearly gap-free" to better reflect its status.

Line 95 - were these parental tissue samples ever used?
Response: Thank you for your insightful comment regarding the parental tissue samples. We will clarify whether the parental tissue samples were utilized in the assembly process and detail how they were incorporated. In our revision, we also included more results of employing a trio-binning approach to generate haplotype resolved assemblies from the two sequenced parental genomes. The initial assembly for the autosomes of the haplotype-resolved genomes was performed by using hifiasm (v0.16.1)[1] and verkko(v1.1)[2] based on the trio mode with HiFi reads, ultra-long ONT reads and and the parents' short reads. The more continuous contigs were selected from the two assemblies to represent. This results in a maternal assembly with 157 gaps and paternal assembly with 99 gaps (Supplementary Table S1). Subsequently, gap closing was performed using TGS-Gapcloser [3] with the verkko assembly, resulting 116 and 42 gaps, respectively. Since our HiFi reads and ultra-long ONT reads are limited, the final diploid JH reference genome has NG50 of 147.60 Mb and 143.06 Mb for maternal and paternal genomes, respectively. And we further evaluated JH.mat and JH.pat with quality values (QV) and coverage distribution of ONT and PacBio HiFi reads. By comparing the linear genomes of two complete haplotypes, we detected ~7.23 million single nucleotide variants (SNVs), 1,165,610 small insertions or deletions (indels) (< 50 bp), and 26,701 SVs (≥ 50 bp).We believe this addition will significantly enhance the value of our manuscript and provide a critical resource for future research in swine genomics.
1. Cheng H, Concepcion GT, Feng X, Zhang H, Li H. Haplotype-resolved de novo assembly using phased assembly graphs with hifiasm. Nat Methods. 2021; doi: 10.1038/s41592-020-01056-5.
2. Rautiainen M, Nurk S, Walenz BP, Logsdon GA, Porubsky D, Rhie A, et al.. Telomere-to-telomere assembly of diploid chromosomes with Verkko. Nat Biotechnol. 2023; doi: 10.1038/s41587-023-01662-6.
3. Xu M, Guo L, Gu S, Wang O, Zhang R, Peters BA, et al.. TGS-GapCloser: A fast and accurate gap closer for large genomes with low coverage of error-prone long reads. GigaScience. 2020; doi:

10.1093/gigascience/giaa094.

Lines 151-156 - this section would be better located below the assembly methods. Please number supplementary tables in order of their appearance in the text.
Response: Thank you for your suggestion regarding Lines 151-156. We agree that this section would be better positioned below the assembly methods. Additionally, we will ensure that the supplementary tables are numbered in the order they appear in the text. Your feedback is greatly appreciated and will help improve the organization of our manuscript.

Line 171 - please provide parameters used here and for all analyses.
Response: Thank you for your request for additional details on the parameters used. We have added the relevant parameters used for all analyses in the manuscript as follows:
λ HiFi reads were assembled using Hifiasm (version 0.16.1-r375) with default parameters.
λ ONT reads were assembled using NextDenovo (version 2.5.0) with default parameters, including genome_size = 2660.49M.
Additionally, more detailed analysis processes and the associated code are publicly available through our GitHub repository: https://github.com/CCyeah/JH-T2T. Thank you for your feedback, which has helped improve the clarity of our manuscript.

Lines 187-188 - how did rearranging contigs decrease the gaps? Was the same gap filling procedure used after HiC manual adjustments?
Response: Thank you for your questions regarding the gap reduction process. Rearranging contigs helped decrease the gaps by optimizing the scaffold structure, allowing for better alignment and integration of contigs. This rearrangement often leads to more accurate placement of sequences, which can effectively close gaps. You can see the detailed process for this step in Figure S3. This figure illustrates how we reduced the gaps by rearranging contigs, we will ensure this is clearly explained in the revised manuscript.

Line 188 - Figure S3 - I don't understand the relationship between the panels nor what the authors are attempting to show. If panels A-C display chromosomes 2, 8, and 13, Why does D display chr3? Both panels C and E are labeled chr13 but they look nothing alike. Are D-E whole chromosomes or zoomed in views? Missing description of panel F.
Response: Thank you for your valuable feedback regarding Figure S3. We have provided a detailed explanation of the relationships between the panels as follows:
λ (A-C) Zoomed-in views Hi-C map of Chromosomes 2, 8, and 13 before correction. Illustrates gaps in Chromosomes 2, 8, and 13 of the JH-T2T assembly that was identified through scaffolding without subsequent editing. (D-E) Hi-C map of Chromosome 3 before correction. Displays Chromosome 3 following the correction of misjoins, with the previously erroneous joins highlighted by red ellipses. Presents Chromosome13 before the misjoins have been rectified, with corrected areas indicated by red ellipses. (F) Hi-C map of Chromosome 13 before correction.

Lines 222-224 - why weren't pig proteins used? Ensembl rapid release has annotated protein datasets for 9 pig assemblies.
Response: Thank you for your question regarding Lines 222-224. We did utilize pig proteins along with those from eleven other closely related species; however, this detail was inadvertently omitted from the text. We will revise the manuscript to clarify our approach and include this important information. Your feedback is greatly appreciated.

Line 264 - although most will know this, make it clear that Sscrofa11.1 is an assembly of a Duroc pig.
Response: Thank you for your suggestion regarding Line 264. We have made it clear in our new main text that Sscrofa11.1 is an assembly derived from a Duroc pig to ensure clarity for all readers. Your feedback is appreciated.

Line 292 - how was polishing performed? This is missing from the methods.
Response: Thank you for your observation regarding Line 292. The term "polishing" has caused some confusion, and we have deleted it to enhance clarity. Your feedback is valuable for improving the completeness of our manuscript.

Line 294 - should this read "selected it for the backbone of the genome assembly."?
Response: Thank you for your suggestion regarding Line 294. This has been amended to read "selected it for the backbone of the genome assembly," which clarifies the intended meaning. Thank you for your suggestion.

Lines 298-299 - methods?
Response: Thank you for your inquiry regarding Lines 298-299. We have added it to methods that NextPolish2 [1] was used to polish the assembly with default parameters. This clarification will ensure a comprehensive understanding of our methods. Your feedback is appreciated and will help enhance the manuscript.
1. Hu J, Wang Z, Liang F, Liu S-L, Ye K, Wang D-P. NextPolish2: A Repeat-aware Polishing Tool for Genomes Assembled Using HiFi Long Reads. Zhao F, editor. Genomics, Proteomics & Bioinformatics. 2024; doi: 10.1093/gpbjnl/qzad009.

Line 314 - what is meant by "using mapped K-mers from trio Illumina PCR-free reads data"?
Response: In our revision, we included employing a trio-binning approach to generate haplotype resolved assemblies from the two sequenced parental genomes. And we used trio Illumina PCR-free reads data to evaluating assembly quality.

Line 331 - accession numbers for assemblies would be useful.
Response: Thank you for your suggestion regarding Line 331. We have included the accession numbers for the assemblies to enhance the clarity and accessibility of our data. Your feedback is appreciated.

Line 333 - what is "properly mapped rate"? Do you mean properly paired mapping rate?
Response: We will clarify what is meant by "properly mapped rate," using terminology such as "properly paired mapping rate" for precision.

Line 346 - what is the historical genome version?
Response: We recognize that the term could lead to misunderstandings regarding draft assemblies. To avoid any confusion, we will remove this phrase from the manuscript. Your feedback is greatly appreciated and will help improve the clarity of our work.

Line 349 - Supplemental Table S8 only has 55 entries including the 6 remaining gaps. Where are the other filled 8 gaps located?
Response: The other filled 8 gaps were resolved through adjustments made to the Hi-C map to correct misassembles. As a result, these gaps cannot be precisely located within the existing order of the assembly. Thank you for your insightful feedback.

Lines 350-358 - read depth displays wouldn't show the presence of clipped reads which would indicate an improperly closed gap. It would be more convincing to display IGV windows containing these alignments showing that there are no clipped reads.
Response: Thank you for your suggestion. We showed the IGV window containing the results of these comparisons using IGV, which showed some clipped reads, but still some reads in the same region that were not clipped, e.g. at 1:153402833-153420736. We understand that structural differences between individual genomes and references can lead to shear mapping [4]. We prefer not to provide IGV images for all gap regions as it is too much, but we provide the raw data that can be viewed in the corresponding window. Thank you for your suggestion.

4. Yang C, Zhou Y, Song Y, Wu D, Zeng Y, Nie L, et al.. The complete and fully-phased diploid genome of a male Han Chinese. Cell Res. 2023; doi: 10.1038/s41422-023-00849-5.

Line 354 - Figure S5 needs a better legend. What is ref and what is own?
Response: Thank you for your feedback regarding Line 354. We will improve the legend for Figure S5 to clearly define "ref" as Sscrofa11.1 and "own" as JH-T2T, providing a more comprehensive explanation of the data presented. Your suggestion is appreciated and will enhance the clarity of the figure.

Line 359 - the assembly is near-gapless.
Response: Thank you for your suggestion. We have state that the assembly is "near-gapless" and provide data supporting the claim of phasing.

Line 359 - where is the data regarding assembly phasing? How was this determined to be fully phased?
Response: Thank you for your question regarding Line 359. We will include the specific data related to assembly phasing and clarify the methods used to determine that the assembly is fully phased. This information will be added to the revised manuscript to enhance transparency and understanding. Your feedback is greatly appreciated.

Line 363 - 16 of 20 chromosomes are gapless.
Response: We have addressed the finding that 16 out of 20 chromosomes are gapless. Thank you for your valuable feedback.

Line 370 - only 33 telomeres were found at the expected location (end of the chromosome), if you count the telomere on chr2 59kb from the end, then 34 telomeres were identified.
Response: Thank you for your observation regarding Line 370. We will clarify that only 33 telomeres were found at the expected locations at the ends of the chromosomes. This clarification will be included in the revised manuscript. Your feedback is appreciated.

Line 372 - chr13 also only has a single telomere. It does not have a telomere at the beginning.
Response: Thank you for your clarification regarding Line 372. We will correct the text to specify that chromosome 13 also has only a single telomere located at the end, and does not have a telomere at the beginning. This revision will ensure accurate representation in the manuscript. Your feedback is appreciated.

Line 372 - chr19 is chrX correct?
Response: Thank you for your question regarding Line 372. Yes, chromosome 19 refers to chromosome X in this context. We will clarify this in the revised manuscript to avoid any confusion. Your feedback is greatly appreciated.

Line 374 - Figure 1G - It would be nice to have the centromeres marked on this plot (or in Figure 3A). Are the long blocks of telomeric repeats internal to the chromosomes expected?
Response: Thank you for your suggestions regarding Line 374. We have marked the centromeres on Figure 1G and/or Figure 3A for better clarity. Additionally, we will address whether the long blocks of telomeric repeats found internally within the chromosomes are expected, providing an explanation in the revised manuscript. These interstitials or pericentromeric telomeric sequences (ITS) have been evidenced as relics of genome rearrangements in some vertebrate’s species. Your feedback is valuable and will help enhance the figures.

Line 423 - Figure 3A - there is no telomeric repeat at the beginning of chr4 or chrX
Response: Thank you for your observation regarding Line 423. We will update the manuscript to clarify that there is no telomeric repeat at the beginning of chromosome 4 or chromosome X as shown in Figure 3A. Your feedback is valuable and will help improve the accuracy of our work.

Line 431 - why were only 5 pigs of each breed used to validate SVs when 100's of WGS datasets from the two breeds had been aligned? How were these 5 selected?
Response: We provided the genotype dataset for the 905 individual pigs extracted from the PHARP database as SNP annotations on the download site. The remaining 5 pigs of each breed were used in the form of raw data, rather than genotype data. The selection of the 5 pigs from each breed for SV validation was based on this available data. Thank you for your question, which will help improve the clarity of our study.

Line 481 - Sscrofa11.1 only has 544 gaps.
Response: We will clarify that Sscrofa11.1 has 544 gaps in the context of our comparison. This detail will be included in the revised manuscript to ensure clarity. Thank you for your feedback.

Line 492 - ONT data was used to fill more than 6 gaps. Gaps in the assembly were reduced from 63 to 14 using ONT contigs.
Response: We have amended that ONT data was used to fill more than 6 gaps, reducing the total number of gaps in the assembly from 63 to 14 using ONT contigs. This information will be included in the revised manuscript for better clarity. Thank you for your suggestion.

Lines 588-589 - please make your code publicly available through zenodo, github, figshare, or something similar.
Response: We appreciate your suggestion for sharing our code publicly, and we have made our code publicly available through github(https://github.com/CCyeah/JH-T2T).

Line 815-824 - Figure 2 - legend description needs to be improved. Only A is mapping rates, B and C are PM rates and base error rates. The color switch from A-C having European pigs in blue to D having JH-T2T in blue might confuse readers.
Response: Thank you for your feedback regarding Lines 815-824 and the legend for Figure 2. We have improved the legend description to clearly indicate that only panel A represents mapping rates, while panels B and C show PM rates and base error rates, respectively. Additionally, we will address the color switch issue, ensuring consistency in color representation throughout the figure to avoid confusing readers. Your suggestions are greatly appreciated and will enhance the clarity of the figure.

Reviewer #3: A telomere-to-telomere or near gapless genome assembly of the domestic pig (Sus scrofa) will be a valuable resource for researchers working on pigs both in an agricultural context and as a biomedical model. The authors report the production of such a pig genome sequence assembly.
Response: Thank you for your thorough review and constructive feedback. We appreciate your insights and will address each of your comments in the revised manuscript.

Abstract
The abstract states that some of the genes highlighted in the study are associated with domestication. No such evidence is presented in the manuscript and nor is domestication mentioned in the results or discusssion section of the manuscript.
Response: We have revised the abstract to remove related section with domestication.

Introduction
The introductory text cites the relevant literature and sets out the need for, and potential benefits of, the new genome assembly.
Response: We appreciate your positive feedback regarding the literature cited. We will ensure clarity in the need for the new genome assembly.

Methods
The laboratory methods are described briefly, but in sufficient detail to facilitate replication.
Response: Thank you for your comment regarding the Methods section. We aimed to provide a brief overview of the laboratory methods while ensuring there is sufficient detail to facilitate replication of our work. We appreciate your feedback and will consider any specific areas for improvement you may suggest.

Lines 138-139: the tissues sampled need further clarification. For example, surcerebellum and incerebellum are not terms in common usage.
Response: Thank you for your comment regarding Lines 138-139. We have clarified the terminology used for sampled tissues, specifically replacing "surcerebellum" and "incerebellum" with standard terms "cerebellar cortex" and "cerebellar medulla". Your feedback is valuable and will help improve the accuracy of our work.

Lines 249-251: thresholds of TPM > 0 and expression in one sample are very lax. In effect no filter has been applied to the expression data and the authors risk reporting expression artefacts.
Response: Thank you for your observation regarding Lines 249-251. We used the thresholds of TPM > 0 and expression in one sample to identify expressed genes, as referenced [5]. While we recognize that these thresholds may seem lax, they were chosen based on standard practices in the field. However, we

appreciate your concern about the potential for reporting expression artefacts.
5. Miao J, Wei X, Cao C, Sun J, Xu Y, Zhang Z, et al.. Pig pangenome graph reveals functional features of non-reference sequences. J Anim Sci Biotechnol. 2024; doi: 10.1186/s40104-023-00984-4.

Results
line 293-4: rephrase "we selected it for the bone genome assembly" - this makes no sense in english.
Response: This has been amended to read " selected it for the backbone of the genome assembly," clarifying the intended meaning. Thank you for your suggestion.

Table S1: in the title "assembly" rather than "assemblying"
Response: In Table S1, we have corrected the title to "assembly". Thank you for your advice.

Table S2: what does "mount rate" mean
What is meant by the term "clusters"? The description of each chromosome in this table appears to suggest that "clusters" are contigs that have been scaffolded to assemble chromosomes comprising n clusters/contigs with n-1 gaps and the gaps spanned using the Hi-C data.
Response:
Mount Rate: the percentage of number of contig to form chromosomes on all contigs. We will added the term "mount rate" in Table S2(new Table S3) to ensure that its meaning is clearly defined.
Clusters: The term "clusters" refers to groups of contigs that have been scaffolded together to form chromosomes. In the context of the table, it indicates that each chromosome comprises n clusters/contigs, with n-1 gaps that were spanned using the Hi-C data. We will enhance the description in the table to make this definition clear.

Table S3 requires a legend as it is not self-explanatory. There are also anomalies in this table. Apparently the telomere (up) (presumably the telomere on the p-arm) has no length, yet occupies the position from 3-19990 that suggests it has a length of 19987 bp.
Response: Thank you for your observations regarding Table S3. We have added a legend to explain the table's contents more clearly, as it is currently not self-explanatory. Additionally, we will address the anomaly you mentioned, where the telomere (up) appears to have no length while occupying a position that suggests a length of 19987 bp. We will correct this inconsistency and ensure that the table accurately reflects the data. Your feedback is greatly appreciated.

There are several places where the links between the text and the supplementary tables appear to be wrong.
Lines 356-357: the citation of Table S11 here does not appear to be correct.
Lines 379 - 380: the citation of Table S13 here does not appear to be correct.
Line 380: the citation of Table S17 here does not appear to be correct.
Lines 406 - 407: the citation of Table S12 here does not appear to be correct.
Response: Thank you for pointing out the discrepancies in the citations to the supplementary tables. We will review and correct the following:
Lines 356-357: Correct the citation.
Lines 379-380: Thank you for pointing out the discrepancy regarding Line 378. We mistakenly referenced Supplementary Table S13, which actually contains a list of SV-enriched QTL rather than the 23,924 genes. We will correct this reference in the revised manuscript. Additionally, we plan to publicly share the annotation file that includes detailed information about all the protein-coding genes. Your feedback is greatly appreciated and will help us ensure the accuracy of our manuscript. And we have proceeded to upload the necessary files directly to our public server using the provided credentials so that reviewer can see it when they need (https://alphaindex.zju.edu.cn/ALPHADB/download.html).
Line 380: Correct the citation.
Lines 406-407: Correct the citation.
We appreciate your careful review, and these corrections will enhance the accuracy of our manuscript.

Table S10 needs a legend and an indication of which genes are gained and which genes are lost. The legend needs to include a statement on which genome's coordinates are being used.
Response: Thank you for your feedback regarding Table S10. We have added a legend to the table(new

Table S11) that clearly indicates which genes are gained and which are lost. Additionally, the legend will specify which genome's coordinates are being used. Your suggestions will enhance the clarity and usability of the table.

Lines 451 - 452: Supplementary Table S18 is missing from the materials submitted with the manuscript.
Response: Thank you for bringing this to our attention. We mistakenly referenced Supplementary Table S18; it should actually refer to new Table S18. We have corrected this in the manuscript. Thank you for your attention to this detail.

Discussion
The authors highlight the main findings of their study and do not overinterpret the results. However, the discussion text is somewhat repetitive and could be shortened.
Response: Thank you for your feedback on the Discussion section. We appreciate your observation regarding the repetitiveness of the text. We will work on shortening the discussion while maintaining clarity and ensuring that the main findings are effectively highlighted. Your input is valuable for improving the manuscript.

Figures
The figures could be improved with more detailed legends. In Figure 2, NGS is used in the figure, but WGS in the legend. WGS would be better, but with an explanation to indicate that WGS refers to short read WGS. Figure 3D - validated and not validated rather than validate and invalidate.
Figure 3A. Annotating the centromeres in JH-T2T would be a useful guide as to whether the large gaps between JH-T2T and Sscrofa11.1 are largely around these structures.
Response: Thank you for your suggestions regarding the figures. We will make the following improvements: Legends: We have enhanced the legends for all figures to provide more detailed explanations.
Figure 2: We have used WGS to replace NGS in the figure, in the meanwhile we clarified that "WGS" refers to short read whole genome sequencing on legends and ensure consistency in terminology.

Figure 3D: We have revised the terms to use "validated" and "not validated" instead of "validate" and "invalidate."

Figure 3A: We have annotated the centromeres in the JH-T2T assembly to provide a clearer understanding of the large gaps in relation to these structures.

Your feedback is greatly appreciated and will help improve the clarity and utility of the figures.

Editorial Manager®

Cookie Preference Center

We use cookies which are necessary to make our site work. We may also use additional cookies to analyze, improve and personalize our content and your digital experience. For

more information, see our Cookie Policy.

You may choose not to allow some types of cookies. However, blocking some types may impact your experience of our site and the services we are able to offer. See the different category headings below to find out more or change your settings.

Allow all

Manage Consent Preferences

Strictly Necessary Cookies

Always active

These cookies are necessary for the website to function and cannot be switched off in our systems. They are usually only set in response to actions made by you which amount to a request for services, such as setting your privacy preferences, logging in or filling in forms. You can set your browser to block or alert you about these cookies, but some parts of the site will not then work. These cookies do not store any personally identifiable information.

Functional Cookies

These cookies enable the website to provide enhanced functionality and personalisation. They may be set by us or by third party providers whose services we have added to our pages. If you do not allow these cookies then some or all of these services may not function properly.

Performance Cookies

These cookies allow us to count visits and traffic sources so we can measure and improve the performance of our site. They help us to know which pages are the most and least popular and see how visitors move around the site.

Targeting Cookies

These cookies may be set through our site by our advertising partners. They may be used by those companies to build a profile of your interests and show you relevant adverts on other sites. If you do not allow these cookies, you will experience less targeted advertising.

Cookie List

Clear

Apply Cancel

Consent Leg.Interest

Confirm my choices

Powered by onetrust
