# Supplementary material for: A near telomere-to-telomere genome assembly of the Jinhua pig: enabling more accurate genetic research
Source: Gigascience. 2025 May 15;14:giaf048. doi: 10.1093/gigascience/giaf048 (PMC12080228; doi:10.1093/gigascience/giaf048)
Supplement: giaf048_Authors_Response_To_Reviewer_Comments_Revision_1 [file giaf048_authors_response_to_reviewer_comments_revision_1.pdf]

Author's Response To Reviewer Comments

Response Letter to the Reviewers
Response to Editor's Comments
Thank you for providing the reviewers'feedback on our revised manuscript, we are pleased to resubmit our manuscript entitled "A Telomere-to-Telomere Genome Assembly of the Jinhua Pig: enabling more accurate genetic research" (GIGA-D-24-00462R1). We appreciate the time and effort the reviewers have taken to evaluate our work. We have carefully addressed their comments and made the necessary revisions to the manuscript. Below, we provide a detailed response to each reviewer's concerns.
We have revised the title to better align with the focus of the manuscript. The new title is:"A Telomere-to-Telomere Genome Assembly of the Jinhua Pig: enabling more accurate genetic research".We believe this title more accurately represents the contributions of our work.
We sincerely appreciate the reviewer's careful evaluation of our methods. To address these concerns, we have:Clarified the Scaffolding and gapfilling Process:
Corrected HiC Data Usage: We acknowledge the reviewer's point that HiC interaction maps do not provide information for gap filling. We have revised the manuscript to clarify that HiC data was used solely for scaffolding and not for gap filling. After re-examining the Hi-C interaction map, we identified and removed specific contigs that were causing misassemblies or lacked sufficient interaction signals. Three small upstream contig on chr3 and several downstream contigs on chr13 were removed. Since these contigs were located at the ends of the chromosomes, their removal eliminated the corresponding gaps without disrupting the overall assembly. Meanwhile, it was observed that after removing the small upstream contig on chr3 and several downstream contigs on chr13, the number of identified telomeres significantly increased. We have rewrote it as "After re-examining the Hi-C interaction map, we removed 3 upstream contigs on chr3 and several downstream contigs on chr13, resulting in 2 gaps and 0 gaps on chr3 and chr13. The final JH-T2T genome has only 6 gaps." at line 190-193. We hope these revisions address the reviewer's concerns and provide a more accurate and transparent description of our methods.
We have further polished the language and formatting of the manuscript to ensure clarity and adherence to journal guidelines.
We believe that the revised manuscript addresses all the reviewers' concerns and provides a more accurate and comprehensive account of our work. We are grateful for the constructive feedback, which has significantly improved the quality of our manuscript. Should there be any further questions or additional revisions required, please do not hesitate to contact us.
Thank you for considering our revised manuscript for publication in GigaScience.

Reviewer #1: In their revised version of the manuscript, the authors have addressed all my major concerns raised in my earlier review and have made the many editorial edits as suggested. I only have a few (mostly editorial) comments for the revised version. The most important one is the title of the manuscript. I realize I did not mention this in my earlier review, but I think the title is not very appropriate and could be more informative. I suggest something like "A telomere-to-telomere genome assembly of the Jinhua pig"
Response: Thank you for your positive feedback on our revised manuscript and for your valuable suggestion regarding the title. We appreciate your thoughtful input and have carefully considered your recommendation. We agree with your suggestion that the title could be more informative and better reflect the completeness and specificity of our work. We have revised the title to:"A Telomere-to-Telomere Genome Assembly of the Jinhua Pig: enabling more accurate genetic research." This new title emphasizes the completeness of the assembly (telomere-to-telomere), specifies the pig breed (Jinhua pig), and highlights the broader impact of the work on genetic research. We have carefully reviewed the manuscript and made additional editorial improvements, including:
λCorrecting minor grammatical errors and enhancing sentence clarity.
λEnsuring consistency in terminology and formatting throughout the manuscript.
λUpdating figure and table captions for better readability.
We believe these changes have further improved the quality and clarity of the manuscript. Thank you again for your valuable feedback and for helping us enhance our work.

Minor editorial comments-
Line 40: Replace "provides" by "provide"; "genome" to "genomes" and "JH" to "Jinhua"
Response: Thank you for your suggestion. We have changed "provides" to " provide" ; "genome" to "genomes" and "JH" to "Jinhua" at Lines 40. Your feedback is greatly appreciated and will help improve the clarity of our work.

Lines 50-51: "This study produced a gapless and near-gapless assembly of the pig genome, and provides a set of diploid JH reference genome." Should be changes to something like "This study produced a near-gapless assembly of the pig genome and provides a set of haploid Jinhua reference genomes."
Response: Thank you for your suggestion. We have changed " This study produced a gapless and near-gapless assembly of the pig genome, and provides a set of diploid JH reference genome." to " This study produced a near-gapless assembly of the pig genome and provides a set of haploid Jinhua reference genomes." at Lines 50-51. Your feedback is greatly appreciated and will help improve the clarity of our work.

Line 177: Change "with with" to "with"
Response: Thank you for your suggestion. We have changed "with with" to "with" at Lines 177. Your feedback is greatly appreciated and will help improve the clarity of our work.

Line 194: Replace "population" by "populations"
Response: Thank you for your suggestion. We have changed "population" by "populations" at Lines 196. Your feedback is greatly appreciated and will help improve the clarity of our work.

Lines 232-233: Referring to human as a "closely related species" is rather awkward and not correct. I suggest replacing this with "eleven other mammals"
Response: Thank you for your suggestion. We have changed "closely related species" by "eleven other mammals" at Lines 233-234. Your feedback is greatly appreciated and will help improve the clarity of our work.

Lines 299, 301 and 303: Insert "of" after "consisting"
Response: Thank you for your suggestion. We have insert "of" after "consisting" at line 298, 300 and 302. Your feedback is greatly appreciated and will help improve the clarity of our work.

Line 317: Insert "and" before "2.33 Gb"
Response: Thank you for your suggestion. We have insert "and" before "2.33 Gb" at line 316. Your feedback is greatly appreciated and will help improve the clarity of our work.

Line 319: Insert "and" before "2.17 Gb"
Response: Thank you for your suggestion. We have insert "and" before "2.17 Gb" at line 318. Your feedback is greatly appreciated and will help improve the clarity of our work.

Line 320-321: Change to "The more continuous contigs of the two assemblies were selected to construct the final haploid assemblies".
Response: Thank you for your suggestion. We changed the sentence from lines 319-320 to "The more continuous contigs of the two assemblies were selected to construct the final haploid assemblies". Your feedback is greatly appreciated and will help improve the clarity of our work.

Line 323: Replace "assembly" by "assembler"
Response: Thank you for your observation regarding Line 323. We recognize that the term could lead to misunderstandings regarding the verkko haplotype assemblies. To avoid any confusion, we have replaced "assembly" by " haplotype assemblies ". Your feedback is greatly appreciated and will help improve the clarity of our work.

Line 354: Delete "ranging"
Response: Thank you for your suggestion. We have deleted "ranging" at line 354. Your feedback is greatly

appreciated and will help improve the clarity of our work.

Lines 358-359: Change "The average properly mapped rate" to "The average rate of properly mapped reads"
Response: Thank you for your suggestion. We have changed "The average properly mapped rate" to "The average rate of properly mapped reads" at line 358-359. Your feedback is greatly appreciated and will help improve the clarity of our work.

Line 379: Insert "respectively" after "60.07"
Response: Thank you for your suggestion. We have insert "respectively" after "60.07" at line 381. Your feedback is greatly appreciated and will help improve the clarity of our work.

Line 380: "suggested" (remove space)
Response: Thank you for your suggestion. We have removed space at line 382. Your feedback is greatly appreciated and will help improve the clarity of our work.

Line 385: Change "indicate a gapless and near-gapless" to "indicate a near-gapless"
Response: Thank you for your suggestion. We have changed "indicate a gapless and near-gapless" to "indicate a near-gapless" at line 387. Your feedback is greatly appreciated and will help improve the clarity of our work.

Line 455: Change "were overlapped with" to "were overlapping with"
Response: Thank you for your suggestion. We have changed "were overlapped with" to "were overlapping with" at line 457. Your feedback is greatly appreciated and will help improve the clarity of our work.

Lines 557-559" The sentence "The insertion found in the SLA-DOB gene, which serves to enhance the immune system's response and is relevant to transplant rejection" seems incomplete and sound awkward. Perhaps you mean something like "The insertion found in SLA-DOB, a gene involved in enhancing the immune system's response to infection, might be relevant in relation to transplant rejection"
Response: Thank you for your suggestion. We have changed the sentence at line 560-562 to "The insertion found in SLA-DOB, a gene involved in enhancing the immune system's response to infection, might be relevant in relation to transplant rejection". Your feedback is greatly appreciated and will help improve the clarity of our work.

Reviewer #2: The first near-complete genome assembly of pig: enabling more accurate genetic research

General comments:
I thank the authors for addressing most of my points and providing more details on the parameters they have used. Unfortunately, I still have some unanswered questions regarding the methodology. My current understanding from the authors responses to my previous comments leads me to believe that the assembly has been scaffolded incorrectly. If the authors did indeed use HiC data to place 8 contigs into gaps and then joined those contigs without placing gaps at the joins or doing any further gap filling, that calls into question the validity of the assembly. Finally, the language needs further improvement for readability.
Response: Thank you for your continued engagement with our manuscript and for raising important concerns regarding the methodology and language. We sincerely appreciate your feedback and have carefully addressed your comments to ensure the accuracy and clarity of our work. Thank you for raising this critical concern regarding the use of Hi-C interaction maps and the gap-filling process. We appreciate your feedback and have carefully revisited our methodology to ensure accuracy and clarity. We acknowledge that Hi-C interaction maps are not for gap filling and should only be used for scaffolding (ordering and orienting contigs). After re-examining the Hi-C interaction map, we identified and removed specific contigs that were causing misassemblies or lacked sufficient interaction signals. Three small upstream contig on chr3 and several downstream contigs on chr13 were removed. Since these contigs were located at the ends of the chromosomes, their removal eliminated the corresponding gaps without disrupting the overall assembly. Meanwhile, it was observed that after removing the small upstream contig on chr3 and several downstream contigs on chr13, the number of identified telomeres significantly increased. We have rewrote it as "After re-examining the Hi-C interaction map, we removed 3 upstream

contigs on chr3 and several downstream contigs on chr13, resulting in 2 gaps and 0 gaps on chr3 and chr13. The final JH-T2T genome has only 6 gaps." at line 190-193.Thank you again for your time and consideration. We hope these revisions have addressed all the concern. Thank you again for your time and constructive feedback. If you have any further questions or require additional information about this part, please do not hesitate to contact us.

Below is our point-by-point response to your concerns.

Specific comments:
Line 85 - *will contribute to.
Response: Thank you for your suggestion. We have changed "will contributed to" to "will contribute to" at line 85. Your feedback is greatly appreciated and will help improve the clarity of our work.

Lines 187-191 - HiC interaction maps do not provide information for gap filling. Either this has been explained insufficiently, or it has been done incorrectly. Placing assembled sequences in the correct order does not mean that it is okay to join them without a gap. It is necessary to return to the gap filling procedure now that the contigs are in the correct order and attempt to fill them as done previously.
Response:Thank you for raising this critical concern regarding the use of Hi-C interaction maps and the gap-filling process. We appreciate your feedback and have carefully revisited our methodology to ensure accuracy and clarity. We acknowledge that Hi-C interaction maps are not for gap filling and should only be used for scaffolding (ordering and orienting contigs). During the process, we identified and removed specific contigs that were causing misassemblies or lacked sufficient interaction signals. Three small upstream contig on chr3 and several downstream contigs on chr13 were removed. Since these contigs were located at the ends of the chromosomes, their removal eliminated the corresponding gaps without disrupting the overall assembly. Meanwhile, it was observed that after removing the small upstream contig on chr3 and several downstream contigs on chr13, the number of identified telomeres significantly increased. We have rewrote it as "After re-examining the Hi-C interaction map, we removed 3 upstream contigs on chr3 and several downstream contigs on chr13, resulting in 2 gaps and 0 gaps on chr3 and chr13. The final JH-T2T genome has only 6 gaps." at line 190-193.Thank you again for your time and consideration. Your feedback is greatly appreciated and will help improve the clarity of our work.

Line 191 - Figure S3 - These HiC contact maps are not very informative they need to be labeled and have a scale bar. Additionally, contact maps can have a lack of signal due to a gap in the sequence or due to multimapping reads in repetitive regions being filtered so it's not clear what they are trying to show in A-C. The authors reply to my previous concern regarding the labeling of this figure does not help, furthermore, the figure legend in the supplemental materials is still insufficient. I think I understand that panels D and E are chr3 before and after misassembly correction, it would be helpful if the two panels were at the same scale. I still don't know why panel F is shown, how is this related to panel C and I don't see any red ellipses indicated by the legend.
Response:Thank you for raising this critical concern regarding Figure S3. We agree that the figure did not effectively clarify the process and may have caused confusion and unnecessarily complicated the intended message. As a result, we have revised the text in the main manuscript to provide a clearer and more detailed explanation and deleted Figure S3 to avoid misunderstanding. Three small upstream contig on chr3 and several downstream contigs on chr13 were removed. Since these contigs were located at the ends of the chromosomes, their removal eliminated the corresponding gaps without disrupting the overall assembly. Meanwhile, it was observed that after removing the small upstream contig on chr3 and several downstream contigs on chr13, the number of identified telomeres significantly increased. Your feedback is greatly appreciated and will help improve the clarity of our work.

Line 275 - "ensemble from Duroc pigs" is incorrect. It is an "assembly of a Duroc pig".
Response: Thank you for your suggestion. We have changed "ensemble from Duroc pigs " to "assembly of a Duroc pig" at line 275. Your feedback is greatly appreciated and will help improve the clarity of our work.

Lines 299, 301, 303 - "containing" not "consisting"
Response: Thank you for your suggestion. We have changed "consisting" to "consisting of " at line 298, 300, 302. Your feedback is greatly appreciated and will help improve the clarity of our work.

Lines 306-308 - Again, HiC data orders and orients contigs, but it does not fill gaps. Please clarify how the assembly was reduced from 14 gaps to 6 gaps with HiC data. Was an additional round of gap filling performed?
Response: Thank you for your comment regarding the use of Hi-C data and the reduction of gaps from 14 to 6. We appreciate your feedback and have revised the text to clarify the process in Materials and methods. Three small upstream contig on chr3 and several downstream contigs on chr13 were removed. Since these contigs were located at the ends of the chromosomes, their removal eliminated the corresponding gaps without disrupting the overall assembly. And we have replaced "for mapping error correction" by "for scaffolding" to avoid misunderstanding at line 311. Your feedback is greatly appreciated and will help improve the clarity of our work.

Lines 313-314 - How is the contig N50 larger than the scaffold N50 above?
Response: Thank you for your observation regarding Line 313-314. We recognize that the term could lead to misunderstandings regarding the scaffold N50. To avoid any confusion, we have replaced "contig N50" by "scaffold N50". Your feedback is greatly appreciated and will help improve the clarity of our work.

Lines 335-336 - Does this refer to the Merqury analysis? I don't think "using mapped K-mers" is correct here, please reword.
Response: Thank you for your observation regarding Line 335-336. We recognize that the term is not correct here, we have reward this as "The estimated QV score is 55, which corresponds to 99.997% accuracy.". Your feedback is greatly appreciated and will help improve the clarity of our work.

Lines 367-368 - what does it mean that "8 out of 63 gaps were corrected" is this from the HiC ordering of contigs?
Response:Thank you for your question regarding the statement "8 out of 63 gaps were corrected" in Lines 368. Three small upstream contig on chr3 and several downstream contigs on chr13 were removed. Since these contigs were located at the ends of the chromosomes, their removal eliminated the corresponding gaps without disrupting the overall assembly. We have rewritten the text as "only 6 gaps left in our final JH assembly" to avoid misunderstanding at line 369. And we still kown how the number of gaps was reduced from 63 to 6 by reading materials and methods. Your feedback is greatly appreciated and will help improve the clarity of our work.

Line 369 - what does the mapping between Sscrofa11.1 and JH-T2T shown in figure S6 have to do with the JH-T2T gap filling being described here?
Response: Thank you for your question regarding the relevance of Figure S6 to the description of JH-T2T gap filling in Line 369. Figure S6 illustrates the mapping between the JH assembly before gap filling and the JH-T2T assembly. We have carefully revised both the figure and the accompanying text to ensure clarity and accuracy. Your feedback is greatly appreciated and will help improve the clarity of our work.

Line 369 - I previously asked about this supplemental table only containing 55 entries. The authors response "The other filled 8 gaps were resolved through adjustments made to the Hi-C map to correct misassembles. As a result, these gaps cannot be precisely located within the existing order of the assembly." indicates that contigs must have been incorrectly joined solely based on the HiC signal between contigs. The authors must know what contigs were added or joined to form the final assembly. It would be trivial to align the two assembly versions and identify the positions of the old contigs in the new assembly. I believe that these incorrectly joined contigs should be broken and put through the same gap filling procedure as performed earlier.
Response:Thank you for your critical feedback regarding the supplementary table containing only 55 entries and for raising concerns about the use of Hi-C interaction maps. We appreciate your attention to detail and have carefully addressed your comments. Below is our response and the actions taken:
We acknowledge that Hi-C interaction maps are not suitable for gap filling and should only be used for scaffolding (ordering and orienting contigs). In our revised manuscript, we have explicitly clarified that Hi-C data was used exclusively for scaffolding and not for gap filling.
During the Hi-C scaffolding process, we identified and removed specific contigs that were causing misassemblies or lacked sufficient interaction signals. Specifically:Three small upstream contigs on chr3 and several downstream contigs on chr13 were removed. Since these contigs were located at the ends of the chromosomes, their removal eliminated the corresponding gaps without disrupting the overall

assembly.No gaps were retained in these regions after the removal of the contigs.
We believe these revisions address your concern and improve the accuracy and clarity of the manuscript. Thank you again for your valuable feedback, which has helped enhance the quality of our work.

Lines 375-378 - Dramatic coverage changes in read mappings as found in these figures are usually indicative of assembly errors. I do not agree that "These findings confirmed the accuracy and reliability" of the assembly. I suggest replacing the last sentence with something more measured such as "Although supported by some read data, the inconsistency of coverage across these gap filled regions suggests that caution should be used when interpreting findings in these regions, cross-referencing results with the gap positions (Supplementary Table S9) is advised."
Response: Thank you for your insightful comment regarding the interpretation of coverage changes in the read mappings. We agree that dramatic coverage changes can sometimes indicate assembly errors, and we appreciate your suggestion to adopt a more measured tone in describing these findings. Below is our response and the revised text: Although supported by some read data, the inconsistency of coverage across these gaps filled regions suggests that caution should be used when interpreting findings in these regions, cross-referencing results with the gap positions (Supplementary Table S9) is advised. Thank you again for your valuable feedback, which has helped enhance the quality of our work.

Line 375 - "evidenced by fully coverage" remove "fully", it isn't proper usage of the word and I wouldn't interpret the low coverage in many of these regions as "full coverage".
Response: Thank you for catching this oversight and for your suggestion to improve the wording. We agree that the term "fully coverage" is improper and does not accurately reflect the observed low coverage in some regions. We have removed the word "fully" at line 376 and rephrased the sentence for clarity and accuracy. Your feedback is greatly appreciated and will help improve the clarity of our work.

Line 385 - should read "Overall, our assembly quality metrics indicate a near-gapless assembly of the pig genome"
Response: Thank you for your suggestion. We have changed "indicate a gapless and near-gapless" to "indicate a near-gapless" at line 387. Your feedback is greatly appreciated and will help improve the clarity of our work.

Line 390 - should read "a gapless T2T sequence for 16 out of 20"
Response: Thank you for your suggestion. We have changed "a gapless T2T sequence for all 16 out of 20" to "a gapless T2T sequence for 16 out of 20" at line 392. Your feedback is greatly appreciated and will help improve the clarity of our work.

Line 396 - Supplemental table 10 not 9.
Response: Thank you for your suggestion. We have changed " Supplemental table 19" to "Supplemental table 10" at line 398. Your feedback is greatly appreciated and will help improve the clarity of our work.

Lines 398-399 - according to supplemental table S4 and figure 3A, chromosome 2 also has a single telomere.
Response: Thank you for your suggestion. We have added chromosome 2 at lines 401. Your feedback is greatly appreciated and will help improve the clarity of our work.

Line 402 - the centromeres are not marked in Figure 3A.
Response: Thank you for pointing out this oversight. We appreciate your attention to detail and have addressed the issue regarding the centromeres in Figure 3A. We have updated Figure 3A to clearly mark the centromeres. The centromeric regions are now indicated with appropriate labels or annotations to ensure they are easily identifiable. Your feedback is greatly appreciated and will help improve the clarity of our work.

Line 402 - Figure S8 - please rename chr19 and chr20, chrX and chrY.
Response: Thank you for pointing out this inconsistency in Figure S8. We appreciate your attention to detail and have addressed the issue by renaming the chromosomes as suggested. We have updated Figure to correctly label the chromosomes as chrX and chrY instead of chr19 and chr20. Thank you again for your valuable feedback, which has helped enhance the quality of our manuscript.

Line 406 - "at early research" unclear what is meant by this. please reword.
Response: Thank you for pointing out the unclear phrasing "at early research" in Line 408. We have removed this phrase and strengthening the connection to relevant literature would improve the clarity and academic rigor of the manuscript. We appreciate your feedback and have revised the text to improve clarity and precision.

Line 423 - as indicated on line 397, 33 telomeres were identified, not 35.
Response: Thank you for pointing out this oversight. We have changed "35 telomeres " to "33 telomeres" at line 425. Your feedback is greatly appreciated and will help improve the clarity of our work.

Line 426 - "The JH-T2T assembly IDENTIFIED 17 centromeres"
Response: Thank you for your suggestion. We have changed "The JH-T2T assembly 17 centromeres" to "The JH-T2T assembly identified 17 centromeres" at line 428. Your feedback is greatly appreciated and will help improve the clarity of our work.

Line 450 - "are located in"
Response: Thank you for your suggestion. We have changed "is located in" to "are located in" at line 452. Your feedback is greatly appreciated and will help improve the clarity of our work.

Line 453 - "these SVs are located in"
Response: Thank you for your comment regarding the sentence on Line 453. We have changed " these SVs located in " to " these SVs are located in " at line 455. Your feedback is greatly appreciated and will help improve the clarity of our work.

Line 455 - Moreover, 12,129 genes overlap these SVs"
Response: Thank you for your comment regarding the sentence on Line 455. We have changed "were overlapped with " to "were overlapping with " at line 457 for better understanding. Your feedback is greatly appreciated and will help improve the clarity of our work.

Line 502 - "which contained 544 gaps"
Response: Thank you for your suggestion. We have changed "which contained of 544 gaps " to "which contained 544 gaps" at line 504. Your feedback is greatly appreciated and will help improve the clarity of our work.

Line 841 - Figure 2 legend description is still incorrect. Only A is mapping rates, B and C are PM rates and base error rates.
Response: Thank you for pointing out the inaccuracy in the legend description for Figure 2. We appreciate your attention to detail and have corrected the legend to accurately reflect the content of each panel. The legend has been updated to correctly describe each panel of Figure 2. The revised legend now reads: "(A-C) Comparison of DNA sequencing read mapping rates, PM rates and base error rates when whole genome resequencing reads from Asian (Left) and European (Right) pig mapped to MSCAAS v1, Ningxiang (NX), Sscrofa11.1 and JH-T2T genome assemblies, respectively."

Cookie Preference Center

We use cookies which are necessary to make our site work. We may also use additional cookies to analyze, improve and personalize our content and your digital experience. For more information, see our Cookie Policy.

You may choose not to allow some types of cookies. However, blocking some types may impact your experience of our site and the services we are able to offer. See the different category headings below to find out more or change your settings.

Allow all

Manage Consent Preferences

Strictly Necessary Cookies

Always active

These cookies are necessary for the website to function and cannot be switched off in our systems. They are usually only set in response to actions made by you which amount to a request for services, such as setting your privacy preferences, logging in or filling in forms. You can set your browser to block or alert you about these cookies, but some parts of the site will not then work. These cookies do not store any personally identifiable information.

Functional Cookies

These cookies enable the website to provide enhanced functionality and personalisation. They may be set by us or by third party providers whose services we have added to our pages. If you do not allow these cookies then some or all of these services may not function properly.

Performance Cookies

These cookies allow us to count visits and traffic sources so we can measure and improve the performance of our site. They help us to know which pages are the most and least popular and see how visitors move around the site.

Targeting Cookies

These cookies may be set through our site by our advertising partners. They may be used by those companies to build a profile of your interests and show you relevant adverts on other sites. If you do not allow these cookies, you will experience less targeted advertising.

Cookie List

Clear

Apply Cancel
Consent Leg.Interest

Confirm my choices

Powered by onetrust
