# Supplementary material for: A near telomere-to-telomere genome assembly of the Jinhua pig: enabling more accurate genetic research
Source: Gigascience. 2025 May 15;14:giaf048. doi: 10.1093/gigascience/giaf048 (PMC12080228; doi:10.1093/gigascience/giaf048)
Supplement: giaf048_Authors_Response_To_Reviewer_Comments_Revision_2 [file giaf048_authors_response_to_reviewer_comments_revision_2.pdf]

Author's Response To Reviewer Comments

Response Letter to the Reviewers
Response to Editor's Comments

Thank you for your email and for providing the final comments from the reviewer. We appreciate the opportunity to address these minor points and ensure our manuscript meets the high standards of GigaScience.

We have carefully revised the manuscript according to the reviewer's suggestions:

The title has been updated to reflect the reviewer's advice, and it now reads: "A near Telomere-to-Telomere Genome Assembly of the Jinhua Pig: Enabling More Accurate Genetic Research."

We have ensured that all URLs and internet sources are included in the bibliography and cited by reference number, as per the journal's style guidelines.All accession numbers have been explicitly mentioned in the Data Availability section.

We have also conducted a thorough review of the manuscript to ensure full compliance with the GigaScience style and formatting requirements. Please let us know if any additional adjustments are needed.

We look forward to working with your data curators to prepare the supporting data for release via GigaDB. Please do not hesitate to contact us if further information or clarification is required.

Thank you again for your guidance and support throughout the review process.

Reviewer #2: The first near-complete genome assembly of pig: enabling more accurate genetic research

General comments:
The authors have clarified how their HiC manual curation efforts were able to remove gaps from the assembly. This was my only remaining major issue. I only have a few minor comments remaining.
Response:Thank you for your thoughtful feedback and for acknowledging our efforts in clarifying the Hi-C manual curation process. We are pleased that the explanation regarding the removal of gaps from the assembly addressed your major concern.

Minor comments:
Line 1 - Title: "A Near Telomere-to-Telomere Genome Assembly of the Jinhua Pig"
Response:We agree with your suggestion that the title could be more informative and better reflect the completeness and specificity of our work. We have revised the title to:"A Near Telomere-to-Telomere Genome Assembly of the Jinhua Pig: enabling more accurate genetic research." Your feedback is greatly appreciated and will help improve the clarity of our work.

Line 369 - replace "only 6 gaps left in our final JH assembly" with "only 6 gaps remain in our final JH assembly"
Response:Thank you for pointing out this language issue. We have revised the sentence on line 369 as suggested. The updated sentence now reads:"only 6 gaps remain in our final JH assembly."We appreciate your attention to detail, which has helped improve the clarity and readability of our manuscript.

Line 370 - Figure S5 needs a more detailed legend
Response:Thank you for your suggestion to improve the legend for Figure S5. We agree that a more detailed legend will help readers better understand the figure and its significance. We have revised the legend to provide additional context and clarity. The updated legend for Figure S5 now reads:Figure S5. Comparison of gap regions between the reference genome (Ref) and the JH T2T genome. The figure shows the syntenic relationship between the JH assembly before gap filling (Ref) and the JH T2T genome,

particularly focusing on the gap regions.

Line 405 - I just noticed this, but are the authors proposing that chr9 has 2 centromeres? Given the know pig karyotype (metacentric chr9), it seems more likely that they have identified some other form of tandem repeat at the beginning of chr9.
Response:Thank you for your keen observation regarding the potential interpretation of chr9 in our assembly. We appreciate your insight and agree that the presence of two centromeres on chr9 would be unusual, given the known metacentric karyotype of the pig. Upon re-examining the data, we believe it is indeed more likely that the observed tandem repeat at the beginning of chr9 represents a different type of repetitive sequence rather than a second centromere. The revised text now reads:"For the chromosome assemblies of chr9, 2 regions harbouring centromeric repeats were identified. The tandem repeat identified at the beginning of chr9 is unlikely to represent a second centromere, given the known metacentric karyotype of the pig. Instead, it may represent another form of tandem repeat, which warrants further investigation."We thank you for catching this important detail, and we believe this revision strengthens the accuracy and clarity of our manuscript. Please let us know if further clarification or adjustments are needed.

Editorial Manager®

Cookie Preference Center

We use cookies which are necessary to make our site work. We may also use additional cookies to analyze, improve and personalize our content and your digital experience. For more information, see our Cookie Policy.

You may choose not to allow some types of cookies. However, blocking some types may impact your experience of our site and the services we are able to offer. See the different category headings below to find out more or change your settings.

Allow all

Manage Consent Preferences

Strictly Necessary Cookies

Always active

These cookies are necessary for the website to function and cannot be switched off in our systems. They are usually only set in response to actions made by you which amount to a request for services, such as setting your privacy preferences, logging in or filling in forms. You can set your browser to block or alert you about these cookies, but

some parts of the site will not then work. These cookies do not store any personally identifiable information.

Functional Cookies

These cookies enable the website to provide enhanced functionality and personalisation. They may be set by us or by third party providers whose services we have added to our pages. If you do not allow these cookies then some or all of these services may not function properly.

Performance Cookies

These cookies allow us to count visits and traffic sources so we can measure and improve the performance of our site. They help us to know which pages are the most and least popular and see how visitors move around the site.

Targeting Cookies

These cookies may be set through our site by our advertising partners. They may be used by those companies to build a profile of your interests and show you relevant adverts on other sites. If you do not allow these cookies, you will experience less targeted advertising.

Cookie List

Clear

Apply Cancel
Consent Leg.Interest

Confirm my choices

Powered by onetrust
