# Supplementary material for: A near telomere-to-telomere genome assembly of the Jinhua pig: enabling more accurate genetic research
Source: Gigascience. 2025 May 15;14:giaf048. doi: 10.1093/gigascience/giaf048 (PMC12080228; doi:10.1093/gigascience/giaf048)
Supplement: giaf048_Authors_Response_To_Reviewer_Comments_Revision_3 [file giaf048_authors_response_to_reviewer_comments_revision_3.pdf]

Author's Response To Reviewer Comments

Subject: Revised Manuscript Submission - GIGA-D-24-00462R3

Dear Dr Hans Zauner,

Thank you for your email and the positive decision regarding our manuscript, "A Near Telomere-to-Telomere Genome Assembly of the Jinhua Pig: enabling more accurate genetic research" (GIGA-D-24-00462R3). We appreciate the editorial team's thorough review and are pleased to submit the revised version addressing all points raised.

Below is a summary of the changes made:

1.GigaDB Dataset Citation: Added the provided citation (DOI: 10.5524/102692) to the reference list and updated the Data Availability section accordingly.
2.GitHub Repository: Cited the GitHub URL (https://github.com/CCyeah/JH-T2T) in the bibliography and referenced it in the Availability section.
3.PhARP URL: Moved to the bibliography and cited by reference number.
4.NCBI BioProject Accession: Added the accession number (PRJNA1238047) to the Data Availability section.
5.Additional URLs: Moved URLs (lines 214, 216, 578) to the bibliography and cited them numerically.
6.ORCIDs: Included all available ORCIDs on the title page.
7.RRIDs: Added RRIDs for key bioinformatics tools (e.g., MAKER: RRID:SCR_005309) in the Methods section.
8.Highlighting: Removed all red highlighting from the peer-review process.
9.Code Availability: Deleted the line "computer code is available on request" as it is now hosted on GitHub.
10.Journal Style: Ensured full compliance with GigaScience's Instructions for Authors.

The revised manuscript has been uploaded to the online submission system (EditorialManager). Please let us know if further adjustments are needed. We appreciate your time and support in bringing this work to publication.

Best regards,
Sincerely,Caiyun Cao
College of Animal Sciences, Zhejiang University, Hangzhou, China.
E-mail: ccyun@zju.edu.cn

Editorial Manager®

Cookie Preference Center

We use cookies which are necessary to make our site work. We may also use additional cookies to analyze, improve and personalize our content and your digital experience. For more information, see our Cookie Policy.

You may choose not to allow some types of cookies. However, blocking some types may impact your experience of our site and the services we are able to offer. See the different category headings below to find out more or change your settings.

Allow all

Manage Consent Preferences

Strictly Necessary Cookies

Always active

These cookies are necessary for the website to function and cannot be switched off in our systems. They are usually only set in response to actions made by you which amount to a request for services, such as setting your privacy preferences, logging in or filling in forms. You can set your browser to block or alert you about these cookies, but some parts of the site will not then work. These cookies do not store any personally identifiable information.

Functional Cookies

These cookies enable the website to provide enhanced functionality and personalisation. They may be set by us or by third party providers whose services we have added to our pages. If you do not allow these cookies then some or all of these services may not function properly.

Performance Cookies

These cookies allow us to count visits and traffic sources so we can measure and improve the performance of our site. They help us to know which pages are the most and least popular and see how visitors move around the site.

Targeting Cookies

These cookies may be set through our site by our advertising partners. They may be used by those companies to build a profile of your interests and show you relevant adverts on other sites. If you do not allow these cookies, you will experience less targeted advertising.

Cookie List

Clear

Apply Cancel
Consent Leg.Interest

Confirm my choices

Powered by onetrust
