# Supplementary material for: A near telomere-to-telomere genome assembly of the Jinhua pig: enabling more accurate genetic research
Source: Gigascience. 2025 May 15;14:giaf048. doi: 10.1093/gigascience/giaf048 (PMC12080228; doi:10.1093/gigascience/giaf048)
Supplement: giaf048_Reviewer_1_Report_Original_Submission — Martien Groenen, Ph.D. -- 11/20/2024 [file giaf048_reviewer_1_report_original_submission.pdf]

**Reviewer Report**

**Title: A Near Telomere-to-Telomere Genome Assembly of the Jinhua Pig: enabling more accurate genetic research**

**Version: Original Submission** **Date:** 11/20/2024

**Reviewer name: Martien Groenen, Ph.D.**

**Reviewer Comments to Author:**

The manuscript describes the T2T genome assembly for the Chinese pig breed Jinhua, which presents a vast improvement compared to the current reference genome of the Duroc pig TJTabasco (build11.1). The results and methodology use for the assembly are described clearly and the authors show the improvement of this assembly by a detailed comparison with the current reference 11.1. While clearly of interest to be published, several aspects of the manuscript should be improved. Most of these changes are minor modifications or inaccuracies in the presentation of the results. However, there are two major aspects that need further attention.1. The T2T assembly presented, represents a combination of the two haplotypes of the pig sequenced. I am surprised why the authors did not also develop two haplotype resolved assemblies of this genome. Haplotype resolved assemblies will be the assemblies of choice for future developments of a reference pan-genome for pigs. The authors describe that they have sequenced the two parents of the sequenced F1 individual, so why did they not use the trio-binning approach to also develop haplotype resolved assemblies. I, think adding these to the manuscript would be a vast improvement for this important resource.2. The results described for the identification of selective sweep regions is not very convincing. This analysis shows differences in the genomes of two breeds: Duroc and Jinhua. However, these breeds have a very different origin of domestication of wild boars that diverged 1 million years ago, followed by the development of a wide range of different breeds selected for different traits. Therefore, the comparison made by the authors cannot distinguish between differences in evolution of Chinese and European Wild Boar, more recent selection after breed formation and even drift. To be able to do so, these analyses would need the inclusion of additional breeds and wild boars from China and Europe. Alternatively, the authors can decide to tone down this part of the manuscript or even delete it altogether, as it does not add to the major message of the manuscript.Minor commentsLine 34: Change the sentence to: "with thousands of segments and centromeres and telomeres missing"Line 37: Insert "and Hi-C" after "long reads"Line 46: Delete " such as GPAM, CYP2C18, LY9, ITLN2, and CHIA"Line 54: Insert "potential" before "xenotransplantation"Line 82: Delete "in response to the gap of a T2T-level pig genome" as this does not add anything and the use of "gap" in this context is confusing.Line 93: Change "The fresh blood" to "Fresh blood"Line 100: The authors need to provide a reference for the SDS methodLines 152-153, line 444, and table S6: This is confusing. The authors mention Genotypes from 939 individuals, but in the table it is shown that they have used WGS data. You need to describe how the WGS data was used to call the genotypes for these individuals. Furthermore, in line 444 you mention 289 JH pigs and 616 DU pigs which together is 905. What about the other 34 individuals shown in table S6?Line 244: Replace "were" by "was" and delete "the" before "fastp"Lines 287292: Here you use several times "length of xx Gb and yy contigs". This is

not correct as the value for the contigs refers to a number and not a length. Rephase e.g. like "length of xx Gb and consisting of yy contigs"Line 294: The use of "bone" sems strange. Either use "backbone" or "core"Line 306: Replace "chromosome" by "genome"Lines 308-309: For the comment "Second, 16 of the 20 chromosomes were each represented by a single contig" you refer to figure 1D however from this figure it cannot be seen if the different chromosomes consist of a single or multiple contigs.Line 346: Do you mean build 11.1 with "historical genome version". If so, please use that instead.Line 349: "post-gap filled"Line 353: The largest gap is 35 kb not 36 kb.Figures 2F-I should be better explained in the legends and the main text (lines 353-358).Lines 378: For the 23,924 genes you refer to supp table S13. However, that table shows a list of SV enriched QTL not these genes. Furthermore, I checked all tables but a table with all the protein coding genes is missing.Line 380: For the 799 newly anchored genes, refer to table S10. Now you refer to table S17 which shows genes enriched KEGG pathways.Lines 383-386: For the higher gene density in GC rich regions, you refer to figure 1D, but it is impossible to see this correlation from figure 1D. For the density of genes and telomeres, you refer to figure 1G. However, that figure does not show gene densities only repeat densities.Line 406-407. This should be table S11.Lines 409-412: For this result you refer to table S11. However, that table only shows data for the gained genes, not the lost genes.Lines 419-420: You refer to table S12 and figure 3B, but the information is only shown in figure 3B and not in table S12.Line 420: Replace "were" by "is"Line 422: Better to use "repeats" instead of "they"Line 425: "Moreover, 12,129 genes located in these SVs". Unclear to what "these" refers to and I assume that you mean genes that (partially) overlap with SVs? Also, this is an incomplete sentence (verb missing). Likewise, this number is not very meaningful as many of these SVs are within introns. It is much more informative to mention for how many genes SVs affect the CDS.Line 433 and table S14: This validation is not clear at all. What exactly are these numbers that are shown? You also mention "greater than 1.00" but the table does not contain any number that is greater than 1.00.Line 435: "Table" not "Tables"Line 436: Change to " SVs with a length larger than 500 bp"The term "invalidate" in figure 3D is rather awkward. Better to use "not-validated" and "validated" in this figure.Line 449: This should be Table S16.Line 452: There is not Table S18Lines 484-486: Change to "Similarly, in human, the use of the T2T-CHM13 genome assembly yields a more comprehensive view of SVs genome-wide, with a greatly improved balance of insertions and deletions [61]."Lines 500-501: Change to "For example, in human, the T2T-CHM13 assembly was shown to improve the analysis of global"Lines 517-528: This paragraph should be deleted as these genes have already been annotated and described in previous genome builds including 11.1. Why discuss these genes here? Following that line of thinking, almost every gene of the 20,000 can be discussed.Line 532: "%" instead of "%%" and insert "which" after "SVs"Lines 537-542: These sentences should be deleted. It is common knowledge that second generation sequencing is not very sensitive to identify SVs. The authors also do not provide any results about dPCR.Line 544: "affect" rather than "harbor"Lines 544-547: This is repetitive and has been stated multiple times so better to delete.Line 561: "which is serve to immune system's response and relevant to transplant rejection" This is an incorrect sentence and should rephrased.Lines 562-568: I don't agree with is statement and suggest to remove it from the discussion.

**Level of Interest**

Please indicate how interesting you found the manuscript: Choose an item.

**Quality of Written English**

Please indicate the quality of language in the manuscript: Choose an item.

**Declaration of Competing Interests**

Please complete a declaration of competing interests, considering the following questions:

- Have you in the past five years received reimbursements, fees, funding, or salary from an organisation that may in any way gain or lose financially from the publication of this manuscript, either now or in the future?
- Do you hold any stocks or shares in an organisation that may in any way gain or lose financially from the publication of this manuscript, either now or in the future?
- Do you hold or are you currently applying for any patents relating to the content of the manuscript?
- Have you received reimbursements, fees, funding, or salary from an organization that holds or has applied for patents relating to the content of the manuscript?
- Do you have any other financial competing interests?
- Do you have any non-financial competing interests in relation to this paper?

If you can answer no to all of the above, write 'I declare that I have no competing interests' below. If your reply is yes to any, please give details below.

I declare that I have no competing interests

I agree to the open peer review policy of the journal. I understand that my name will be included on my report to the authors and, if the manuscript is accepted for publication, my named report including any attachments I upload will be posted on the website along with the authors' responses. I agree for my report to be made available under an Open Access Creative Commons CC-BY license (http://creativecommons.org/licenses/by/4.0/). I understand that any comments which I do not wish to be included in my named report can be included as confidential comments to the editors, which will not be published.

Choose an item.

To further support our reviewers, we have joined with Publons, where you can gain additional credit to further highlight your hard work (see: https://publons.com/journal/530/gigascience). On publication of this paper, your review will be automatically added to Publons, you can then choose whether or not to claim your Publons credit. I understand this statement.

Yes Choose an item.
