# Supplementary material for: A near telomere-to-telomere genome assembly of the Jinhua pig: enabling more accurate genetic research
Source: Gigascience. 2025 May 15;14:giaf048. doi: 10.1093/gigascience/giaf048 (PMC12080228; doi:10.1093/gigascience/giaf048)
Supplement: giaf048_Reviewer_1_Report_Revision_1 — Martien Groenen, Ph.D -- 2/5/2025 [file giaf048_reviewer_1_report_revision_1.pdf]

Reviewer Report

Title: A Near Telomere-to-Telomere Genome Assembly of the Jinhua Pig: enabling more accurate genetic research

Version: Revision 1 **Date:** 2/5/2025

Reviewer name: Martien Groenen, Ph.D.

Reviewer Comments to Author:

In their revised version of the manuscript, the authors have addressed all my major concerns raised in my earlier review and have made the many editorial edits as suggested. I only have a few (mostly editorial) comments for the revised version. The most important one is the title of the manuscript. I realize I did not mention this in my earlier review, but I think the title is not very appropriate and could be more informative. I suggest something like "A telomere-to-telomere genome assembly of the Jinhua pig"Minor editorial commentsLine 40: Replace "provides" by "provide"; "genome" to "genomes" and "JH" to "Jinhua"Lines 50-51: "This study produced a gapless and near-gapless assembly of the pig genome, and provides a set of diploid JH reference genome." Should be changes to something like "This study produced a near-gapless assembly of the pig genome and provides a set of haploid Jinhua reference genomes."Line 177: Change "with with" to "with"Line 194: Replace "population" by "populations"Lines 232-233: Referring to human as a "closely related species" is rather awkward and not correct. I suggest replacing this with "eleven other mammals"Lines 299, 301 and 303: Insert "of" after "consisting"Line 317: Insert "and" before "2.33 Gb"Line 319: Insert "and" before "2.17 Gb"Line 320-321: Change to "The more continuous contigs of the two assemblies were selected to construct the final haploid assemblies".Line 323: Replace "assembly" by "assembler"Line 354: Delete "ranging"Lines 358-359: Change "The average properly mapped rate" to "The average rate of properly mapped reads"Line 379: Insert "respectively" after "60.07"Line 380: "suggested" (remove space)Line 385: Change "indicate a gapless and near-gapless" to "indicate a near-gapless"Line 455: Change "were overlapped with" to "were overlapping with"Lines 557-559" The sentence "The insertion found in the SLA-DOB gene, which serves to enhance the immune system's response and is relevant to transplant rejection" seems incomplete and sound awkward. Perhaps you mean something like "The insertion found in SLA-DOB, a gene involved in enhancing the immune system's response to infection, might be relevant in relation to transplant rejection"

Level of Interest

Please indicate how interesting you found the manuscript: Choose an item.

Quality of Written English

Please indicate the quality of language in the manuscript: Choose an item.

Declaration of Competing Interests

Please complete a declaration of competing interests, considering the following questions:

- Have you in the past five years received reimbursements, fees, funding, or salary from an organisation that may in any way gain or lose financially from the publication of this manuscript, either now or in the future?
- Do you hold any stocks or shares in an organisation that may in any way gain or lose financially from the publication of this manuscript, either now or in the future?
- Do you hold or are you currently applying for any patents relating to the content of the manuscript?
- Have you received reimbursements, fees, funding, or salary from an organization that holds or has applied for patents relating to the content of the manuscript?
- Do you have any other financial competing interests?
- Do you have any non-financial competing interests in relation to this paper?

If you can answer no to all of the above, write 'I declare that I have no competing interests' below. If your reply is yes to any, please give details below.

I declare that I have no competing interests

I agree to the open peer review policy of the journal. I understand that my name will be included on my report to the authors and, if the manuscript is accepted for publication, my named report including any attachments I upload will be posted on the website along with the authors' responses. I agree for my report to be made available under an Open Access Creative Commons CC-BY license (http://creativecommons.org/licenses/by/4.0/). I understand that any comments which I do not wish to be included in my named report can be included as confidential comments to the editors, which will not be published.

Choose an item.

To further support our reviewers, we have joined with Publons, where you can gain additional credit to further highlight your hard work (see: https://publons.com/journal/530/gigascience). On publication of this paper, your review will be automatically added to Publons, you can then choose whether or not to claim your Publons credit. I understand this statement.

Yes Choose an item.
