# Supplementary material for: A near telomere-to-telomere genome assembly of the Jinhua pig: enabling more accurate genetic research
Source: Gigascience. 2025 May 15;14:giaf048. doi: 10.1093/gigascience/giaf048 (PMC12080228; doi:10.1093/gigascience/giaf048)
Supplement: giaf048_Reviewer_2_Report_Original_Submission — Benjamin D Rosen -- 11//27/2024 [file giaf048_reviewer_2_report_original_submission.pdf]

**Reviewer Report**

**Title: A Near Telomere-to-Telomere Genome Assembly of the Jinhua Pig: enabling more accurate genetic research**

**Version: Original Submission** **Date:** 11/27/2024

**Reviewer name: Benjamin D Rosen**

**Reviewer Comments to Author:**

The first near-complete genome assembly of pig: enabling more accurate genetic researchThe authors describe the telomere-to-telomere assembly of a Jinhua breed pig. They sequenced genomic DNA from whole blood with PacBio HiFi and Oxford Nanopore (ONT) long-read technologies as well as Illumina for short reads. They generated HiC data for scaffolding from blood and extracted RNA from 19 tissues for short read RNAseq for gene annotation. A hifiasm assembly was generated with the HiFi data and scaffolded with HiC to chromosome level with 63 gaps. The scaffolded assembly was gap filled with contigs from a NextDenovo assembly of the ONT data bringing the gaps down to 14. Finally, the assembly was manually curated with juicebox somehow closing a further 8 gaps. This needs to be clarified. Standard assembly assessments were performed as well as genome annotation. The authors compared their assembly to the current reference, Sscrofa11.1, and called SVs between the assemblies. The SVs were validated with additional Jinhua and Duroc animals. They then identified signatures of selection present in some of the largest SVs.General comments:The manuscript is mostly easy to read but would benefit from further editing for language throughout. The described assembly appears to be high quality and quite contiguous. Although the authors do mention obtaining parental samples and claim the assembly is fully phased, there is no mention of how this was done. There are many additional places where the methods could be described more fully including the addition of parameters used.Specific comments:Line 39 - Figure 1 only displays 34 telomeres, not 35. Additionally, I was only able to detect 33 telomeres using seqtk telo. Seqtk only reports telomeres at the beginning and end of sequences, digging further, the telomere on chr2 is ~59kb from the end of the chromosome, perhaps indicating a misassembly.Lines 79-81 - there are not hundreds of species with gap free genome assemblies and reference 19 does not claim that there are.Line 82 - the assembly is not gap-free, replace with "nearly gap-free"Line 95 - were these parental tissue samples ever used?Lines 151-156 - this section would be better located below the assembly methods. Please number supplementary tables in order of their appearance in the text.Line 171 - please provide parameters used here and for all analyses.Lines 187-188 - how did rearranging contigs decrease the gaps? Was the same gap filling procedure used after HiC manual adjustments?Line 188 - Figure S3 - I don't understand the relationship between the panels nor what the authors are attempting to show. If panels A-C display chromosomes 2, 8, and 13, Why does D display chr3? Both panels C and E are labeled chr13 but they look nothing alike. Are D-E whole chromosomes or zoomed in views? Missing description of panel F.Lines 222-224 - why weren't pig proteins used? Ensembl rapid release has annotated protein datasets for 9 pig assemblies.Line 264 - although most will know this, make it clear that Sscrofa11.1 is an assembly of a Duroc pig.Line 292 - how was polishing performed? This is missing from the methods.Line 294 - should

this read "selected it for the backbone of the genome assembly."?Lines 298-299 - methods?Line 314 - what is meant by "using mapped K-mers from trio Illumina PCR-free reads data"?Line 331 - accession numbers for assemblies would be useful.Line 333 - what is "properly mapped rate"? Do you mean properly paired mapping rate?Line 346 - what is the historical genome version?Line 349 - Supplemental Table S8 only has 55 entries including the 6 remaining gaps. Where are the other filled 8 gaps located?Lines 350-358 - read depth displays wouldn't show the presence of clipped reads which would indicate an improperly closed gap. It would be more convincing to display IGV windows containing these alignments showing that there are no clipped reads.Line 354 - Figure S5 needs a better legend. What is ref and what is own?Line 359 - the assembly is near-gapless.Line 359 - where is the data regarding assembly phasing? How was this determined to be fully phased?Line 363 - 16 of 20 chromosomes are gapless.Line 370 - only 33 telomeres were found at the expected location (end of the chromosome), if you count the telomere on chr2 59kb from the end, then 34 telomeres were identified.Line 372 - chr13 also only has a single telomere. It does not have a telomere at the beginning.Line 372 - chr19 is chrX correct?Line 374 - Figure 1G - It would be nice to have the centromeres marked on this plot (or in Figure 3A). Are the long blocks of telomeric repeats internal to the chromosomes expected?Line 423 - Figure 3A - there is no telomeric repeat at the beginning of chr4 or chrXLine 431 - why were only 5 pigs of each breed used to validate SVs when 100's of WGS datasets from the two breeds had been aligned? How were these 5 selected?Line 481 - Sscrofa11.1 only has 544 gaps.Line 492 - ONT data was used to fill more than 6 gaps. Gaps in the assembly were reduced from 63 to 14 using ONT contigs.Lines 588-589 - please make your code publicly available through zenodo, github, figshare, or something similar.Line 815-824 - Figure 2 - legend description needs to be improved. Only A is mapping rates, B and C are PM rates and base error rates. The color switch from A-C having European pigs in blue to D having JH-T2T in blue might confuse readers.

**Level of Interest**

Please indicate how interesting you found the manuscript: Choose an item.

**Quality of Written English**

Please indicate the quality of language in the manuscript: Choose an item.

**Declaration of Competing Interests**

Please complete a declaration of competing interests, considering the following questions:

- Have you in the past five years received reimbursements, fees, funding, or salary from an organisation that may in any way gain or lose financially from the publication of this manuscript, either now or in the future?
- Do you hold any stocks or shares in an organisation that may in any way gain or lose financially from the publication of this manuscript, either now or in the future?

- Do you hold or are you currently applying for any patents relating to the content of the manuscript?
- Have you received reimbursements, fees, funding, or salary from an organization that holds or has applied for patents relating to the content of the manuscript?
- Do you have any other financial competing interests?
- Do you have any non-financial competing interests in relation to this paper?

If you can answer no to all of the above, write 'I declare that I have no competing interests' below. If your reply is yes to any, please give details below.

I declare that I have no competing interests

I agree to the open peer review policy of the journal. I understand that my name will be included on my report to the authors and, if the manuscript is accepted for publication, my named report including any attachments I upload will be posted on the website along with the authors' responses. I agree for my report to be made available under an Open Access Creative Commons CC-BY license (http://creativecommons.org/licenses/by/4.0/). I understand that any comments which I do not wish to be included in my named report can be included as confidential comments to the editors, which will not be published.

Choose an item.

To further support our reviewers, we have joined with Publons, where you can gain additional credit to further highlight your hard work (see: https://publons.com/journal/530/gigascience). On publication of this paper, your review will be automatically added to Publons, you can then choose whether or not to claim your Publons credit. I understand this statement.

Yes Choose an item.
