# Supplementary material for: A near telomere-to-telomere genome assembly of the Jinhua pig: enabling more accurate genetic research
Source: Gigascience. 2025 May 15;14:giaf048. doi: 10.1093/gigascience/giaf048 (PMC12080228; doi:10.1093/gigascience/giaf048)
Supplement: giaf048_Reviewer_2_Report_Revision_1 — Benjamin D Rosen -- 2/27/2025 [file giaf048_reviewer_2_report_revision_1.pdf]

Reviewer Report

Title: A Near Telomere-to-Telomere Genome Assembly of the Jinhua Pig: enabling more accurate genetic research

Version: Revision 1 **Date:** 2/27/2025

Reviewer name: Benjamin D Rosen

Reviewer Comments to Author:

The first near-complete genome assembly of pig: enabling more accurate genetic researchGeneral comments:I thank the authors for addressing most of my points and providing more details on the parameters they have used. Unfortunately, I still have some unanswered questions regarding the methodology. My current understanding from the authors responses to my previous comments leads me to believe that the assembly has been scaffolded incorrectly. If the authors did indeed use HiC data to place 8 contigs into gaps and then joined those contigs without placing gaps at the joins or doing any further gap filling, that calls into question the validity of the assembly. Finally, the language needs further improvement for readability.Specific comments:Line 85 - *will contribute to.Lines 187-191 - HiC interaction maps do not provide information for gap filling. Either this has been explained insufficiently, or it has been done incorrectly. Placing assembled sequences in the correct order does not mean that it is okay to join them without a gap. It is necessary to return to the gap filling procedure now that the contigs are in the correct order and attempt to fill them as done previously.Line 191 - Figure S3 - These HiC contact maps are not very informative they need to be labeled and have a scale bar. Additionally, contact maps can have a lack of signal due to a gap in the sequence or due to multimapping reads in repetitive regions being filtered so it's not clear what they are trying to show in A-C. The authors reply to my previous concern regarding the labeling of this figure does not help, furthermore, the figure legend in the supplemental materials is still insufficient. I think I understand that panels D and E are chr3 before and after misassembly correction, it would be helpful if the two panels were at the same scale. I still don't know why panel F is shown, how is this related to panel C and I don't see any red ellipses indicated by the legend.Line 275 - "ensemble from Duroc pigs" is incorrect. It is an "assembly of a Duroc pig".Lines 299, 301, 303 - "containing" not "consisting"Lines 306-308 - Again, HiC data orders and orients contigs, but it does not fill gaps. Please clarify how the assembly was reduced from 14 gaps to 6 gaps with HiC data. Was an additional round of gap filling performed?Lines 313-314 - How is the contig N50 larger than the scaffold N50 above?Lines 335-336 - Does this refer to the Merqury analysis? I don't think "using mapped K-mers" is correct here, please reword.Lines 367-368 - what does it mean that "8 out of 63 gaps were corrected" is this from the HiC ordering of contigs?Line 369 - what does the mapping between Sscrofa11.1 and JH-T2T shown in figure S6 have to do with the JH-T2T gap filling being described here?Line 369 - I previously asked about this supplemental table only containing 55 entries. The authors response "The other filled 8 gaps were resolved through adjustments made to the Hi-C map to correct misassembles. As a result, these gaps cannot be precisely located within the existing order of the assembly." indicates that contigs must have been incorrectly joined solely based on the HiC signal between contigs. The authors must know what contigs were added or joined to form the final assembly.

It would be trivial to align the two assembly versions and identify the positions of the old contigs in the new assembly. I believe that these incorrectly joined contigs should be broken and put through the same gap filling procedure as performed earlier.Lines 375-378 - Dramatic coverage changes in read mappings as found in these figures are usually indicative of assembly errors. I do not agree that "These findings confirmed the accuracy and reliability" of the assembly. I suggest replacing the last sentence with something more measured such as "Although supported by some read data, the inconsistency of coverage across these gap filled regions suggests that caution should be used when interpreting findings in these regions, cross-referencing results with the gap positions (Supplementary Table S9) is advised."Line 375 - "evidenced by fully coverage" remove "fully", it isn't proper usage of the word and I wouldn't interpret the low coverage in many of these regions as "full coverage".Line 385 - should read "Overall, our assembly quality metrics indicate a near-gapless assembly of the pig genome"Line 390 - should read "a gapless T2T sequence for 16 out of 20"Line 396 - Supplemental table 10 not 9.Lines 398-399 - according to supplemental table S4 and figure 3A, chromosome 2 also has a single telomere.Line 402 - the centromeres are not marked in Figure 3A.Line 402 - Figure S8 - please rename chr19 and chr20, chrX and chrY.Line 406 - "at early research" unclear what is meant by this. please reword.Line 423 - as indicated on line 397, 33 telomeres were identified, not 35.Line 426 - "The JH-T2T assembly IDENTIFIED 17 centromeres"Line 450 - "are located in"Line 453 - "these SVs are located in"Line 455 - Moreover, 12,129 genes overlap these SVs"Line 502 - "which contained 544 gaps"Line 841 - Figure 2 legend description is still incorrect. Only A is mapping rates, B and C are PM rates and base error rates.

Level of Interest

Please indicate how interesting you found the manuscript: Choose an item.

Quality of Written English

Please indicate the quality of language in the manuscript: Choose an item.

Declaration of Competing Interests

Please complete a declaration of competing interests, considering the following questions:

- Have you in the past five years received reimbursements, fees, funding, or salary from an organisation that may in any way gain or lose financially from the publication of this manuscript, either now or in the future?
- Do you hold any stocks or shares in an organisation that may in any way gain or lose financially from the publication of this manuscript, either now or in the future?
- Do you hold or are you currently applying for any patents relating to the content of the manuscript?
- Have you received reimbursements, fees, funding, or salary from an organization that holds or has applied for patents relating to the content of the manuscript?
- Do you have any other financial competing interests?

- Do you have any non-financial competing interests in relation to this paper?

If you can answer no to all of the above, write 'I declare that I have no competing interests' below. If your reply is yes to any, please give details below.

I declare that I have no competing interests

I agree to the open peer review policy of the journal. I understand that my name will be included on my report to the authors and, if the manuscript is accepted for publication, my named report including any attachments I upload will be posted on the website along with the authors' responses. I agree for my report to be made available under an Open Access Creative Commons CC-BY license (http://creativecommons.org/licenses/by/4.0/). I understand that any comments which I do not wish to be included in my named report can be included as confidential comments to the editors, which will not be published.

Choose an item.

To further support our reviewers, we have joined with Publons, where you can gain additional credit to further highlight your hard work (see: https://publons.com/journal/530/gigascience). On publication of this paper, your review will be automatically added to Publons, you can then choose whether or not to claim your Publons credit. I understand this statement.

Yes Choose an item.
