# Supplementary material for: A near telomere-to-telomere genome assembly of the Jinhua pig: enabling more accurate genetic research
Source: Gigascience. 2025 May 15;14:giaf048. doi: 10.1093/gigascience/giaf048 (PMC12080228; doi:10.1093/gigascience/giaf048)
Supplement: giaf048_Reviewer_3_Report_Original_Submission — Alan L Archibald -- 12/5/2024 [file giaf048_reviewer_3_report_original_submission.pdf]

Reviewer Report

Title: A Near Telomere-to-Telomere Genome Assembly of the Jinhua Pig: enabling more accurate genetic research

Version: Original Submission **Date:** 12/5/2024

Reviewer name: Alan L Archibald

Reviewer Comments to Author:

A telomere-to-telomere or near gapless genome assembly of the domestic pig (Sus scrofa) will be a valuable resource for researchers working on pigs both in an agricultural context and as a biomedical model. The authors report the production of such a pig genome sequence assembly.AbstractThe abstract states that some of the genes highlighted in the study are associated with domestication. No such evidence is presented in the manuscript and nor is domestication mentioned in the results or discusssion section of the manuscript.IntroductionThe introductory text cites the relevant literature and sets out the need for, and potential benefits of, the new genome assembly.MethodsThe laboratory methods are described briefly, but in sufficient detail to facilitate replication.Lines 138-139: the tissues sampled need further clarification. For example, surcerebellum and incerebellum are not terms in common usage.Lines 249-251: thresholds of TPM > 0 and expression in one sample are very lax. In effect no filter has been applied to the expression data and the authors risk reporting expression artefacts.Resultsline 293-4: rephrase "we selected it for the bone genome assembly" - this makes no sense in english.Table S1: in the title "assembly" rather than "assemblying"Table S2: what does "mount rate" meanWhat is meant by the term "clusters"? The description of each chromosome in this table appears to suggest that "clusters" are contigs that have been scaffolded to assemble chromosomes comprising n clusters/contigs with n-1 gaps and the gaps spanned using the Hi-C data.Table S3 requires a legend as it is not self-explanatory. There are also anomalies in this table. Apparently the telomere (up) (presumably the telomere on the p-arm) has no length, yet occupies the position from 3-19990 that suggests it has a length of 19987 bp.There are several places where the links between the text and the supplementary tables appear to be wrong.Lines 356-357: the citation of Table S11 here does not appear to be correct.Lines 379 - 380: the citation of Table S13 here does not appear to be correct.Line 380: the citation of Table S17 here does not appear to be correct.Lines 406 - 407: the citation of Table S12 here does not appear to be correct.Table S10 needs a legend and an indication of which genes are gained and which genes are lost. The legend needs to include a statement on which genome's coordinates are being used.Lines 451 - 452: Supplementary Table S18 is missing from the materials submitted with the manuscript.DiscussionThe authors highlight the main findings of their study and do not overinterpret the results. However, the discussion text is somewhat repetitive and could be shortened.FiguresThe figures could be improved with more detailed legends. In Figure 2, NGS is used in the figure, but WGS in the legend. WGS would be better, but with an explanation to indicate that WGS refers to short read WGS. Figure 3D - validated and not validated rather than validate and invalidate.Figure 3A. Annotating the centromeres in JH-T2T would be a useful guide as to whether the large gaps between JH-T2T and Sscrofa11.1 are largely around these structures.

Level of Interest

Please indicate how interesting you found the manuscript: Choose an item.

Quality of Written English

Please indicate the quality of language in the manuscript: Choose an item.

Declaration of Competing Interests

Please complete a declaration of competing interests, considering the following questions:

- Have you in the past five years received reimbursements, fees, funding, or salary from an organisation that may in any way gain or lose financially from the publication of this manuscript, either now or in the future?
- Do you hold any stocks or shares in an organisation that may in any way gain or lose financially from the publication of this manuscript, either now or in the future?
- Do you hold or are you currently applying for any patents relating to the content of the manuscript?
- Have you received reimbursements, fees, funding, or salary from an organization that holds or has applied for patents relating to the content of the manuscript?
- Do you have any other financial competing interests?
- Do you have any non-financial competing interests in relation to this paper?

If you can answer no to all of the above, write 'I declare that I have no competing interests' below. If your reply is yes to any, please give details below.

'I declare that I have no competing interests'

I agree to the open peer review policy of the journal. I understand that my name will be included on my report to the authors and, if the manuscript is accepted for publication, my named report including any attachments I upload will be posted on the website along with the authors' responses. I agree for my report to be made available under an Open Access Creative Commons CC-BY license (http://creativecommons.org/licenses/by/4.0/). I understand that any comments which I do not wish to be included in my named report can be included as confidential comments to the editors, which will not be published.

Choose an item.

To further support our reviewers, we have joined with Publons, where you can gain additional credit to further highlight your hard work (see: https://publons.com/journal/530/gigascience). On publication of

this paper, your review will be automatically added to Publons, you can then choose whether or not to claim your Publons credit. I understand this statement.

Yes Choose an item.
